# An Experimental and Modeling Study on the Interaction of Cements with Varying C_3_A Ratios and Different Water-Reducing Admixtures Using the *op*-ANN and Various Machine Learning Methods

**DOI:** 10.3390/polym18050656

**Published:** 2026-03-07

**Authors:** Veysel Kobya, Hasan Tahsin Öztürk, Kemal Karakuzu, Ali Mardani, Naz Mardani

**Affiliations:** 1 Department of Civil Engineering, Bursa Uludag University, 16059 Bursa, Turkey; vkobya@uludag.edu.tr; 2 Department of Civil Engineering, Faculty of Technology, Karadeniz Technical University, 61080 Trabzon, Turkey; 3 Department of Civil Engineering, Ozyegin University, 34794 Istanbul, Turkey; 4 Department of Mathematics Education, Bursa Uludag University, 16059 Bursa, Turkey

**Keywords:** polycarboxylate-based admixtures, cement compatibility, CRA, MARS, *op*ANN

## Abstract

This study investigates the interaction between polycarboxylate-based water-reducing admixtures (WRAs) and various types of CEM I 42.5R Portland cements, focusing on optimizing input parameters in cementitious systems. Despite the widespread use of WRAs to enhance concrete’s workability, strength, and durability, their compatibility with cement remains a critical challenge, often leading to performance issues such as low initial flow, bleeding, and rapid slump loss. This research addresses two significant gaps in the literature: the unexplored use of input parameter reduction in cementitious systems and the application of novel metaheuristic algorithms in optimizing these systems. In this study, 25 WRA were first synthesized to enrich the inputs of machine learning (ML) models. Then, a dataset of 750 entries was generated, and advanced prediction models were developed. To ensure scientific rigor and eliminate data leakage, a triple-split dataset strategy (Training–Validation–Test) and 5-fold cross-validation were implemented. Among the machine learning techniques analyzed, the Optimized Artificial Neural Networks (*op*ANN) architecture decisively demonstrated the highest prediction performance on the isolated test dataset. In the *op*ANN process, 10 different metaheuristics were tested to evaluate their effectiveness in hyperparameter optimization. As a result, the Kepler Optimization (KOA) algorithm was determined as the algorithm with the highest performance in ANN hyperparameter optimization. Furthermore, Shapley Additive Explanations (SHAP) analysis was utilized to bridge the gap between empirical observations and algorithmic predictions, quantitatively corroborating the rheological roles of phosphate and sulfonate groups. The results offer new insights into WRA–cement compatibility and present advanced, interpretable modeling approaches that enhance predictive accuracy, contributing to more reliable and sustainable concrete practices.

## 1. Introduction

Polycarboxylate-based water-reducing admixtures (PCEs) are essential in modern concrete technology for improving workability, strength, and durability by lowering the water-to-cement (W/C) ratio, thereby reducing binder use [1]. However, PCEs can cause negative effects due to cement incompatibility, such as low initial flow, bleeding, rapid slump loss, rapid setting, and set retardation, which affect the performance of cementitious mixtures [2,3]. Key factors influencing PCE–cement compatibility include the cement’s chemical composition (e.g., C_3_A content), fineness, presence of pozzolans, gypsum type, clay content in aggregates, and sulfate levels [3,4,5,6]. For PCEs, important factors include pH, molecular weight, type of anionic monomer (e.g., carboxylate, phosphate, sulfonate), and the lengths and density of the main and side chains [7,8].

To determine cement–PCE compatibility, various tests are performed, including flowability, rheology, setting time, hydration kinetics, and compressive strength. Rheological tests are beneficial for evaluating the fresh properties of cementitious systems. However, finding the optimal W/C ratio and PCE dosage requires extensive preliminary testing [9,10], involving numerous mixtures and experiments to avoid mixtures that are too stiff or too fluid. This process is labor-intensive, time-consuming, and costly, which is why modeling studies are often employed to streamline it [11].

Regression and machine learning (ML) techniques have recently become popular for modeling cementitious systems. Key methods include Artificial Neural Networks (ANNs) [12,13,14,15,16], Classical Regression Analysis [17,18,19,20], Decision Trees [20], and Random Forest [20,21]. ANNs are effective, but optimizing their architecture requires time-consuming trial-and-error, which can be automated using heuristic search algorithms [15,21,22]. Metaheuristics, though less efficient than backpropagation for training, excel in optimizing network architecture for better predictive performance [14,23,24,25,26,27]. Table 1 summarizes recent ML-based studies on cementitious systems.

When analyzing the input parameters of the studies reviewed in Table 1, it is evident that the models predominantly focus on general mixture designs to predict the properties of fresh or hardened concrete. In contrast, the present study offers a more micro-level data architecture by directly integrating the molecular parameters of the WRA from the synthesis phase (e.g., anionic charge density and chain lengths) and the phase composition of the cement into the modeling process. This depth of data positions our work as substantially more comprehensive than similar ML applications in the literature, particularly in its capacity to elucidate the underlying chemical interactions of the system. The studies given in Table 1 above have been analyzed into five items below. In light of the conclusions obtained as a result of this analysis, some gaps in the literature are expected, and how to fill these gaps is explained.

(i) Evaluation of Model Outputs: Most studies on cementitious systems primarily focus on compressive strength. However, some also investigate other factors such as carbonation depth, yield stress, plastic viscosity, PCE adsorption ratio, bleeding, channel flow, CO_2_ footprint, elastic modulus, alkali–silica reaction-induced expansion, flexural strength, splitting tensile strength, and Marsh cone time. Yield stress and viscosity, the outputs of this study, appear in only two studies [15,16], which used ANNs without metaheuristic enhancements.

(ii) Evaluation of ML Techniques Used: The reviewed studies utilized various ML techniques, including Classical Regression Analysis, Decision Trees, M5P-trees, Multiadaptive Regression Splines, Random Forest, Relevance Vector Machines, Support Vector Machines, XGBoost, AdaBoost, CatBoost, KNN, ANFIS, Support Vector Regression etc. Some studies employed classical ANNs, while others hybridized ANNs with metaheuristics, often replacing backpropagation algorithms in training. In three studies, metaheuristics were employed to optimize network hyperparameters. However, it should be noted that these algorithms are not recent.

(iii) Evaluation of Metaheuristics in ANNs: Metaheuristics are commonly used in hybrid ANN studies instead of backpropagation algorithms, which can result in longer computation times. Network architecture design remains a user-driven, trial-and-error process. The present paper aims to address these issues by using metaheuristics to optimize only the network architecture and transfer functions, and also by utilizing the generally accepted backpropagation algorithm as the training algorithm.

(iv) Evaluation of Metaheuristic Algorithms: The studies reviewed employ various metaheuristic algorithms, including Adam optimizer, Biogeography-Based Optimization (BBO), Beta Differential Evolution-Improve Particle Swarm Optimization Algorithm (BDE-IPSO), Cuttlefish Optimization Algorithm (CFOA), Covariance Matrix Adapted Evolution Strategy Algorithm (CMAES), Electromagnetic Field Optimization (EFO), Fruit Fly Optimization Algorithm (FOA), Genetic Algorithm (GA), Grey Wolf Optimizer (GWO), Harris Hawks Optimization (HHO), Lion Swarm Optimization Algorithm (LSO), Particle Swarm Optimization Algorithm (PSO), Runge–Kutta Optimization (RUN), Salp Swarm Algorithm (SSA), Sine Cosine Algorithm (SCA), Slime Mould Algorithm (SMA), Sparrow Search Algorithm (SSA), Water Cycle Algorithm (WCA), and Whale Optimization Algorithm (WOA). Among the metaheuristics used in the studies examined, the oldest is GA (1989), while the newest is BDE-IPSO (2022).

As a result of these evaluations, two key gaps in the literature were identified:

(i) Several competing regression and machine learning methods were applied in the reviewed studies, but it can be seen that only the ANN method was used in the yield stress and viscosity prediction studies, various competing machine learning methods were not tested, and no enhancement was made with metaheuristic algorithms in the studies conducted using the ANN method.

(ii) Few and outdated competing metaheuristic algorithms were used in the studies where ANN hyperparameter optimization was performed.

To address these gaps, paste mixtures with two W/C ratios (0.32 and 0.35) and three water-reducing admixture (WRA) dosages (0%, 0.10%, and 0.15% by weight of cement) were prepared. Testing these mixtures provided a dataset of 750 entries, including rheological parameters such as final viscosity (F.V) and dynamic yield stress (D.YS). Before proceeding to the modeling process, colinearity tests were performed on the experimental data and input parameters with multicollinearity were removed from the models. Modeling studies were conducted using Classical Regression Analysis (CRA), Multivariate Adaptive Regression Splines (MARS), Support Vektor Machines (SVM), Decision Trees (DT), Random Forest (RF), k-Nearest Neighbors (kNN), Model Tree (m5Tree) and Optimized Artificial Neural Networks (*op*ANN) using metaheuristic algorithms. This is the most comprehensive study in the literature in terms of the competing regression and machine learning methods used. In this study, ten up to date metaheuristics, frequently used in engineering problems, are evaluated for their effectiveness in hyperparameter optimization in the *op*ANN framework. The metaheuristic algorithms used in this study, including Escaping Bird Search (EBS), Gaining-Sharing Knowledge-Based (GSK), Kepler Optimization (KOA), Marine Predators (MPA), Rime Optimization (RIME), Snake Optimizer (SO), Symbiotic Organisms Search (SOS), and Teaching Learning-Based Artificial Bee Colony (TLABC), have not been previously tested. This study represents the most extensive evaluation of metaheuristics in the context of hyperparameter optimization that has been conducted among the reviewed studies. The performance of these algorithms was statistically assessed to identify the most robust one. Comparisons between the competitive ML techniques (CRA, MARS, SVM, DT, RF, kNN, m5Tree and *op*ANN) determined which method produced the highest-performing models.

The primary objective of this study is to develop a comprehensive, machine learning (ML)-based decision support framework capable of accurately predicting the interactions between cement and water-reducing admixtures (WRAs). To achieve this overarching goal and integrate the study’s components into a unified workflow, the sub-objectives are structured in a mutually supportive hierarchy. Initially, to enhance the learning capacity and generalizability of the ML models, 25 distinct WRAs with specific molecular properties were synthesized, thereby providing the essential input parameter diversity required for the algorithms to effectively model molecular-level structure–performance relationships. Subsequently, the rheological effects of these synthesized admixtures were systematically characterized, establishing the comprehensive and novel experimental database requisite for the training and validation of the algorithms. In the final phase, by comparing ML methods optimized with contemporary meta-heuristic algorithms, the most robust technique for representing the acquired dataset and predicting rheological parameters with the highest stability was identified. In summary, the chemical synthesis and experimental characterization processes in this study are not isolated or disjointed objectives; rather, they serve as the fundamental building blocks that generate the quantitative data infrastructure absolutely necessary for the success of the advanced modeling and prediction framework.

## 2. Materials and Methods

### 2.1. Materials

Within the scope of the study, 5 different CEM I 42.5R type Portland cements were used. Four of these cements were produced using the same raw material. The fifth cement was obtained from a different cement factory. C_3_A content was taken into account when naming the cements. Since the raw materials used in the different factories’ cements differed from those of other cements, this cement was named CEM I. The properties of all cements obtained from the manufacturers are also given in Table 2.

As part of the study, twenty-five polycarboxylate ether (PCE)-based water-reducing admixtures (WRAs) were synthesized via free-radical copolymerization. The synthesis program was structured into two main categories to systematically investigate the influence of polymer architecture on rheological behavior.

In the first category, seven different 100% carboxylate-type WRAs (WRA1–WRA7) were synthesized with systematically varied main and side chain lengths. The main chain length was defined using a coefficient k, which corresponds to the number of nonionic grafted units along the backbone. For instance, a polymer containing 21 nonionic graft units was denoted as having a backbone length of 21k. Side-chain molecular weights were adjusted independently to evaluate steric-hindrance effects while maintaining comparable grafting densities.

In the second category, the influence of anionic group chemistry was investigated. Eighteen additional WRAs (WRA8–WRA25) were synthesized by partially substituting the 100% carboxylate functionality with phosphate or sulfonate groups at controlled substitution ratios ranging from 1% to 20%. This approach enabled systematic evaluation of both (i) anionic group type and (ii) degree of substitution on adsorption behavior and rheological performance.

All WRAs were synthesized via aqueous free-radical copolymerization under a nitrogen atmosphere to prevent oxygen inhibition. The reactions were conducted at a controlled temperature (±1 °C) using a redox-initiator system. Monomer feeding was performed in a semi-batch manner to ensure controlled polymer growth and minimize compositional drift. Reaction time, initiator concentration, and monomer feed rate were kept constant for all syntheses to ensure comparability among polymers.

The synthesized polymers were characterized using Gel Permeation Chromatography (GPC) to determine number-average molecular weight (Mn), weight-average molecular weight (Mw), and polydispersity index (PDI = Mw/Mn). The molecular weight distributions were consistent with controlled radical polymerization behavior, and PDI values remained within a relatively narrow range, ensuring comparable dispersity across different WRA families.

The molecular characteristics (Mn, Mw, PDI, substitution ratios, and grafting density parameters) are summarized in Table 3.

To assess synthesis reproducibility, selected WRAs representing different structural categories were synthesized in duplicate batches under identical reaction conditions. Comparative GPC analyses revealed deviations in Mw and Mn values below ±5%, and no statistically significant differences were observed in subsequent rheological performance tests. These results confirm good interbatch consistency and synthesis robustness.

### 2.2. Experimental Method

Paste mixtures were prepared with two different water-to-cement (W/C) ratios (0.32 and 0.35) and three WRA dosages (0%, 0.10%, and 0.15% by weight of cement) to determine rheological parameters, including final viscosity (F.V) and dynamic yield stress (D.YS). Preliminary tests indicated that a W/C ratio above 0.35 led to cement paste instability and segregation. Conversely, mixtures with a W/C ratio below 0.32 were too stiff for rheological measurements. Therefore, W/C ratios of 0.32 and 0.35 were selected for evaluating the mixtures’ rheological parameters.

The mixtures were prepared according to the following standardized protocol:

Cement and mixing water were first placed into the mixer bowl and mixed at low speed for 30 s to ensure initial homogenization.

The mixer was then stopped, and any cement paste adhering to the bowl walls was carefully scraped back into the mixture to ensure full incorporation of the material.

The mixture was subsequently mixed at high speed for 2 min. Accordingly, mixtures without chemical admixture were mixed for a total duration of 2.5 min.

For mixtures containing water-reducing admixture (WRA), the following additional procedure was applied:

After completion of the scraping step (Step 2), the WRA corresponding to 0.10% by weight of cement was added to the mixture, followed by high-speed mixing for 2 min.

After completing the rheological measurement of the 0.10% dosage mixture, the same paste was returned to the mixer, and an additional 0.05% WRA (by weight of cement) was added to obtain a total dosage of 0.15%. The mixture was again homogenized under high-speed mixing prior to rheological testing.

This procedure was repeated independently for each water-to-binder (w/b) ratio, cement type, and admixture type.

A rheometer equipped with an 8 mm ball measuring system (MCR52 Ball Measuring System—BMS) (Anton Paar, Graz, Austria) was used to assess the rheological parameters of the paste mixtures. Based on previous studies [2,10,11,29,30], a three-stage rheological protocol was executed. The first stage involved eliminating the shear history of the mixtures by applying a constant shear rate of 5 s^−1^ for 30 s. In the second stage, the flow curve’s ascending part was obtained by linearly increasing the shear rate from 0 to 30 s^−1^ over 150 s. In the final stage, the shear rate was linearly decreased from 30 s^−1^ to 0 over 150 s. Test data were modeled using the Herschel–Bulkley model, and D.YS and F.V values were calculated for each mixture. The D.YS value was determined at the point where the shear stress curve intersected the y-axis, while the F.V was taken as the instantaneous viscosity value at the highest shear rate, where the viscosity curve became nearly horizontal.

Each factorial combination of material and mixture parameters was prepared and tested once under strictly controlled laboratory conditions (constant temperature, identical mixing protocol, same operator, and rheometer setup). Given the high sensitivity of fresh-state rheological measurements to minor variations in shear history and early hydration kinetics, repeated batch preparation may introduce additional variability. Therefore, to ensure consistency across the large parametric space investigated, each formulation was tested only once.

**Figure 1:**
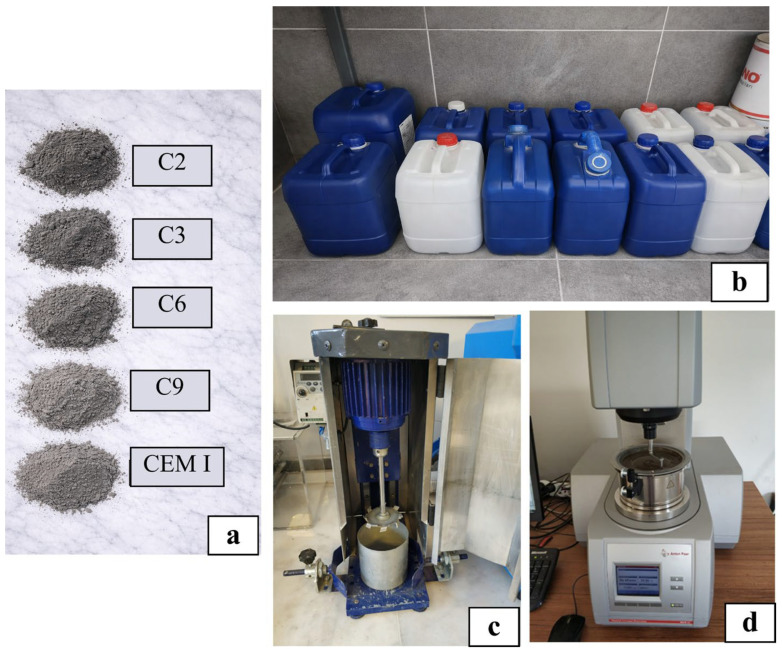
(**a**) Cement types and (**b**) WRA types introduced in the Section 2.1; (**c**) high-speed mixer and (**d**) rheometer mentioned in the Methods section.

Figure 1 presents the cement and WRA introduced in the Materials section, together with the high-speed mixer and rheometer employed in the Methods section.

### 2.3. Data Preparation

The models developed in this study aim to calculate the yield stress and viscosity of concrete using various machine-learning techniques. Two separate models are developed for threshold yield stress and viscosity. The experimental parameters are W/C ratio, cement fineness, C_3_A content, C_3_S content, C_2_S content, C_4_AF content, equivalent alkali value, WRA dosages (% by weight of cement), Mw: average molecular weight (weight), Mn: average molecular weight (number), main chain length, side chain length (g/mole), carboxylate content, phosphate content, sulfonate content, and pH.

In this section, a multicollinearity test is first performed on the experimental parameters to determine the input parameters for the yield stress and viscosity machine learning models. For this purpose, collinearity analysis is performed, and the tolerance and variance inflation factors (VIFs) are calculated. The VIF values of the experimental parameters for the yield stress and viscosity models to be created, and the tolerance and VIF values calculated after the removed parameters are given in Table 4 and Table 5. The extracted parameters are shown in crossed-out cells in the tables.

As it is known, VIF (Variance Inflation Factor) values are used to evaluate multicollinearity between independent variables in multiple linear regression models. If VIF < 1, there is no multicollinearity between independent variables. Although this situation is rarely seen in practice, it indicates that the variables are completely independent of each other. In the range 1 ≤ VIF < 5, there is a moderate level of multicollinearity between the independent variables. This is generally an acceptable range and does not pose a serious problem for the model. In the range 5 ≤ VIF < 10, there is a high level of multicollinearity between the independent variables. This may affect the reliability of the model and should be taken into consideration. Removing some of the variables from the model may be a solution. A VIF ≥ 10 indicates a very serious multicollinearity among the independent variables. This situation seriously affects the reliability of the coefficients of the model and these variables should be removed from the model and transformed.

**Figure 2:**
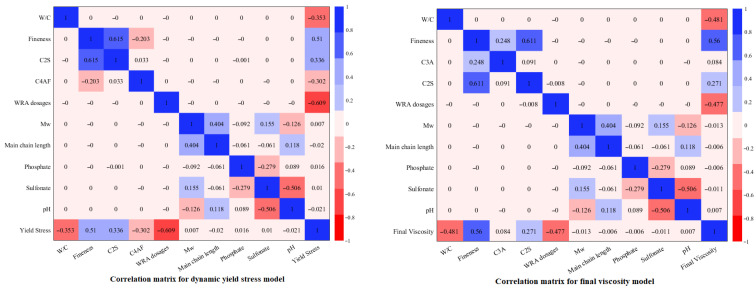
Correlation matrices for dependent and independent variables of dynamic yield stress and final viscosity models.

In light of this evaluation, the parameters with high VIF values for yield stress and viscosity models were removed one by one from the experimental data shown in Table 4 and Table 5 and the VIF values were recalculated. Finally, the VIF values of all parameters were determined to be less than five and given in the last three columns of the tables. After this stage, the input parameters given in the last three columns of Table 4 and Table 5 will be used in the regression and machine learning models to be created for dynamic yield stress and final viscosity. The correlation matrices prepared for the dependent and independent data used for the dynamic yield stress and final viscosity models are shown in Figure 2.

The correlation matrices presented in Figure 2 confirm the absence of multicollinearity among the independent variables identified by VIF analysis for the yield stress and final viscosity models.

#### 2.3.1. Methodology of Modelling

This section provides the machine learning techniques used in the modeling, such as Classical Regression Analysis (CRA), Multivariate Adaptive Regression Splines (MARS), Support Vektor Machines (SVM), Decision Trees (DT), Random Forest (RF), k-Nearest Neighbors (kNN), Model Tree (m5Tree) and optimized artificial neural networks (*op*ANN) using metaheuristic algorithms are presented, and the settings of the metaheuristic algorithms and the computational environment used in the ANN hyperparameter optimization are given.

**Figure 3:**
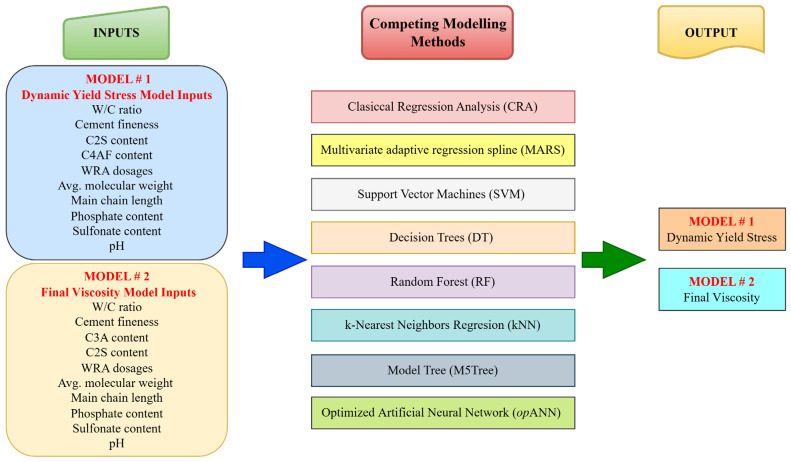
Schematic representation of the methodology used for modeling dynamic yield stress and final viscosity.

Input, output parameters and competing modeling methods used in the modeling process of dynamic yield stress and final viscosity are shown schematically in Figure 3. The dataset, comprising 750 samples, was partitioned using a triple-split strategy to ensure unbiased performance evaluation. In total, 80% of the data (600 samples) was allocated for model development, which was further subdivided during the 5-fold cross-validation process into training and validation sets. The training set was used for weight updates, while the validation set was utilized for hyperparameter tuning and the *op*ANN optimization process. The remaining 20% (150 samples) was strictly isolated as an independent test set and was never exposed to the models during any stage of training or optimization. To prevent data leakage, a selective normalization strategy was adopted; distance-based models (ANN, SVM, and kNN) were normalized in the range of 0.1 to 0.9 using parameters derived solely from the training folds, whereas scale-invariant models (CRA, MARS, RF, DT, and M5Tree) were trained using raw data to maintain engineering interpretability.

To ensure a fair and transparent comparison, a consistent hyperparameter tuning protocol was implemented across all models. For the baseline models (MARS, SVM, DT, RF, kNN, and M5Tree), the optimal configurations were determined using the Grid Search method based on the performance on the validation sets. While the *op*ANN model was optimized using metaheuristic algorithms with 21 independent runs to ensure statistical robustness, the hyperparameters of the baseline models were tuned using the grid search method. The baseline models were optimized using Grid Search, which is a deterministic optimization technique that exhaustively evaluates a predefined discrete parameter space; thus, multiple runs are redundant as the outcome remains invariant. Conversely, metaheuristic algorithms are stochastic by nature. To account for their inherent randomness and to ensure the statistical significance of the results, 21 independent runs were conducted for the *op*ANN model, following the established conventions in optimization literature. The hyperparameter search space and the computational budget for each model are detailed in Table 6, Table 7, Table 8, Table 9, Table 10 and Table 11. In the *op*ANN process, the cost function for the metaheuristic algorithms was explicitly defined as the validation MSE. This approach ensured that the independent test set remained entirely unseen, providing an objective measure of the models’ generalization capabilities on new data. To guarantee the reproducibility of the numerical results, the ‘twister’ algorithm with a fixed random seed (rng(42)) was utilized during the training and validation phases.

#### 2.3.2. Classic Regression Analysis (CRA)

In the initial phase, baseline predictive models were developed using Classical Regression Analysis (CRA). The coefficients of seven fundamental regression functions—linear, power, exponential, inverse, logarithmic, S-curve, and quadratic—were determined using the training dataset and subsequently evaluated on the test set.

#### 2.3.3. Multivariate Adaptive Regression Splines (MARS)

Multivariate Adaptive Regression Splines (MARS) is a non-parametric method capable of modeling complex non-linear relationships and variable interactions without prior assumptions about the underlying data structure [31]. In this study, the MARS modeling process was implemented using the open-source ARESLab toolbox [32] within the MATLAB (Ver 1.13.0) environment. To ensure optimal predictive performance, the specific implementation parameters were strictly tuned via grid search optimization. The exact search space configurations, including maximum basis functions and interaction degrees, along with a computational budget of 13,440 unique trials, are explicitly detailed in Table 6.

#### 2.3.4. Support Vector Machines (SVM)

Support Vector Machines (SVM) project non-linearly separable data into a higher-dimensional feature space using kernel functions to establish an optimal separating hyperplane [33,34]. To effectively capture the complex relationships within the utilized dataset, the SVM hyperparameters were systematically optimized via grid search. The practical implementation settings, including the selection of kernel functions, box constraints, and epsilon margins, resulted in a computational budget of 1008 unique trials, which are comprehensively outlined in Table 7.

#### 2.3.5. Decision Trees (DT)

Decision Trees (DT) capture non-linear relationships by recursively partitioning data based on specific feature thresholds. While highly interpretable, tree depth and leaf sizes must be rigorously constrained to mitigate the risk of overfitting [35,36]. Consequently, the practical implementation of the DT model heavily relied on a grid search optimization to determine the ideal structural boundaries. The detailed hyperparameter search space—encompassing minimum parent/leaf sizes and maximum splits across 3072 unique trials—is presented in Table 8.

#### 2.3.6. Random Forests (RF)

Random Forest (RF) is an ensemble technique that aggregates multiple decision trees trained on random data subsamples and feature subsets to reduce model variance and enhance generalization [37,38]. For this study’s implementation, the RF architecture was fine-tuned using grid search optimization. The specific configurations, including the number of ensemble learning cycles and splitting criteria evaluated over a computational budget of 10,368 unique trials, are strictly defined in Table 9.

#### 2.3.7. K-Nearest Neighbors (kNN)

K-Nearest Neighbors (kNN) estimates target values based on the distance-weighted average of the closest data points [36,39]. Because the predictive accuracy of kNN is inherently sensitive to the number of neighbors (k) and the chosen distance metric, these parameters were rigorously determined through grid search optimization. The precise search space, which included various distance types, weight functions, and k-values resulting in 19,040 unique trials, is specified in Table 10.

#### 2.3.8. Model Tree (m5Tree)

M5 Model Trees integrate the hierarchical decision logic of standard trees with local linear regression functions at the leaf nodes, offering a hybrid approach for modeling non-linear interdependencies [40,41]. To optimize the balance between global data topology and local linear trends, the algorithm’s pruning and sampling mechanisms were tuned via grid search. The specific implementation settings, comprising a computational budget of 4608 unique combinations, are detailed in Table 11.

#### 2.3.9. Optimized Artificial Neural Network (*op*ANN)

**Figure 4:**
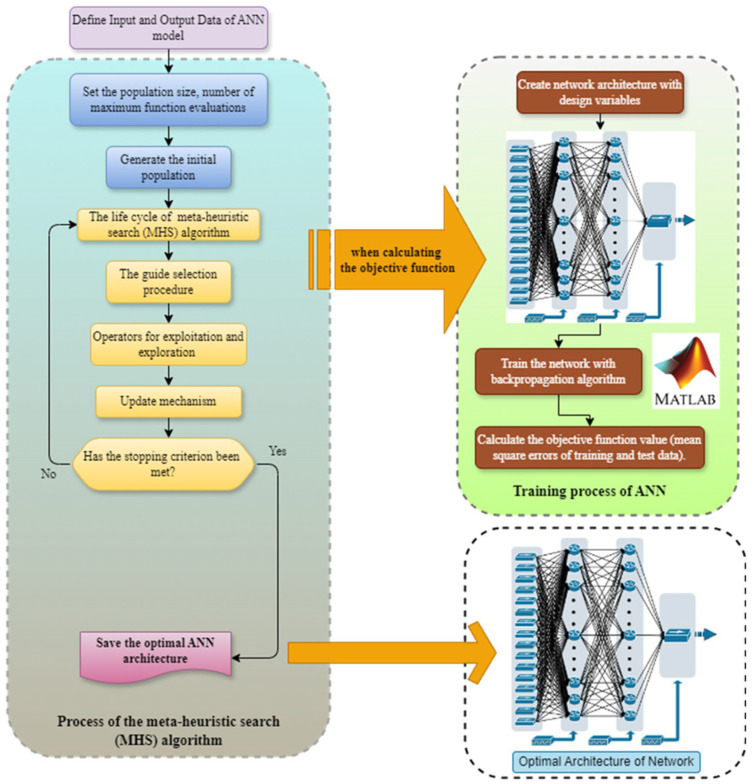
Optimization process of artificial neural network (*op*ANN).

In this study, instead of tuning the ANN hyperparameters by trial and error, it is aimed to transform it into an optimization problem and solve this problem with meta-heuristic algorithms. In the optimization problem designed for this purpose, the objective function is defined as the minimization of the mean squared error (MSE) of the training and test datasets. Optimization problem using all input parameters (*op*ANN-AIP): In this optimization problem, there are five design variables: the number of neurons in the first hidden layer, the number of neurons in the second hidden layer, the types of activation functions in the first hidden layer, the second hidden layer and the output layer. Therefore, in this problem, the optimal number of neurons in the hidden layer(s) of the network and the optimal activation function types for these hidden layers and the output layer are determined. The optimal activation functions are determined by metaheuristic search algorithms, from a pool of 12 different activation functions, including purelin, tansig, logsig, elliotsig, hardlim, hardlims, satlin, satlins, poslin, tribas, radbasi and radbasn. The lower and upper bounds and increment values of the design variables used in these problems are given in Table 12. The flowchart summarizing the optimization process for determining the neural network architecture is depicted in Figure 4.

Performance metrics: The root mean square error (*RMSE*), the Nash–Sutcliffe efficiency coefficient (*NSE*) and Performance Index (*PI*) s are used to evaluate the performance of the ANN. These metrics are calculated according to Equations (1)–(3).

(1) $$RMSE={\left[\frac{1}{n}\sum_{i=1}^{n}{\left(E_{i}-P_{i}\right)}^{2}\right]}^{1/2}$$

(2) $$NSE=1-\frac{\;\sum_{i=1}^{n}{\left(E_{i}-P_{i}\right)}^{2}}{\sum_{i=1}^{n}{\left(E_{i}-\bar{E}\right)}^{2}}$$

(3) $$PI=\frac{RRMSE}{1+R}\;$$

where $E_{i}$ is the experimental (real) output value, $\bar{E}$ is the average of these values, $P_{i}$ is the output value obtained from the model, *RRMSE* is the relative root mean squared error, *R* is the correlation coefficient, and *n* is the total number of data observations. The Nash–Sutcliffe (*NSE*) coefficient is a metric ranging from −∞ to 1. An *NSE* value of 1 indicates that the model is a perfect fit, while an *NSE* value between 0.75 and 1 indicates that the model fit is very good. The range between 0.65 and 0.75 indicates a good model fit, while values between 0.50 and 0.65 indicate that the model is adequate. A *NSE* value below 0.50 indicates that the model is inadequate. The performance index (*PI*) takes values in the range of 0–1. A *PI* close to zero indicates that the model is successful, while a *PI* close to 1 indicates that the model is inadequate.

Friedman’s test [31], a nonparametric statistical test, was used to rank the performance of the metaheuristic algorithms. In addition, the statistics of the optimization findings of the algorithms were also evaluated through blot box plots. Among the metaheuristic algorithms, the first three algorithms with the highest average Friedman score in all problems were identified and proposed as the algorithms with the highest performance.

Metaheuristic optimization algorithms used and their settings: Escaping Bird Search (EBS), Gaining-Sharing Knowledge Based (GSK), Kepler Optimization (KOA), Marine Predators (MPA), Rime Optimization (RIME), Runge–Kutta Optimization (RUN), Slime Mould (SMA), Snake Optimizer (SO), Symbiotic Organisms Search (SOS), Teaching Learning-Based Artificial Bee Colony (TLABC) algorithms were used to simulate the optimization problem and their performances were compared. To make a fair evaluation, the parameter settings recommended by the developers of the metaheuristics were followed and are summarized in Table 13 below. The termination criterion of the algorithm is based on the number of objective function evaluations (maxFEs) and maxFEs are set to 1000 times the number of design variables. For each problem, 21 independent simulations were performed.

Simulation environment: All algorithmic operations were executed on a workstation equipped with an Intel^®^ Xeon^®^ CPU E5-1650v3@3.50GHz processor (İntel, Chengdu, China).

Training algorithm and settings: The Levenberg–Marquardt algorithm, is known to be effective for training the network during the hyperparameter optimization of the developed ANN [52]. In this study, the Levenberg–Marquardt algorithm was implemented through the MATLAB toolbox. In the algorithm, default settings were used in the toolbox. However, the maximum training iteration value was limited to 30 to reduce the computation time during network optimization.

## 3. Result and Discussion

### 3.1. Experimental Results

Due to the large number of cement and WRA types investigated, the experimental results are given in the Supplementary Materials. The experimental results are analyzed under three sub-headings.

#### 3.1.1. Effect of WRA Chain Lengths on Rheological Properties

In this sub-heading, the interaction of WRAs progressing from WRA1 to WRA7 with cements with varying C_3_A ratios was examined through rheological properties.

It was observed that increasing the W/C ratio led to a reduction in both the D.YS and F.V values of the mixtures. Additionally, as the water content increased, the D.YS and F.V values of the WRAs tended to converge. In pastes with a high W/C ratio, the increased distance between cement particles reduces their tendency to agglomerate [53,54,55]. This greater separation makes the dispersion effect of WRAs more apparent [56].

The rheological parameters of cement pastes with a 0.32 W/C ratio and 0.10% WRA dosage showed that the admixture with a medium main chain length (WRA1) exhibited the best compatibility with all cements. Reducing the main chain length (WRA2) and increasing it (WRA3) while keeping the side chain length constant resulted in increases in F.V values. Additionally, the interaction of WRA with C_3_A and ettringite played a crucial role. The C_3_A content in ordinary cements typically ranges from 5 to 10% [5]. An increase in C_3_A content generally has a negative effect on the fresh properties of cement; thus, a lower C_3_A content is recommended to enhance WRA performance [57]. Since ettringite and C_3_A have positive charges, WRA is primarily adsorbed onto aluminate phases [58,59]. Consequently, high C_3_A cements increase the water/WRA demand of the mixture, negatively affecting its fresh-state properties [2,60]. For cement pastes with a 0.32 W/C ratio and WRAs with a fixed main chain, reducing the side chain length of the WRA molecule from 2400 (WRA1) to 1000 g/mole (WRA4), or increasing it to 3000 g/mole (WRA5), resulted in increases in F.V values. These increases also led to improvements in D.YS. The rheological performance of the WRAs followed a similar trend with an increase in the W/C ratio.

WRA1 (medium main and side chain WRA) and WRA7 (short main and long side chain WRA) exhibited similar characteristics. WRA6, which has long main chains and short side chains is the worst performance among these three.

#### 3.1.2. Effect of WRA Anionic Group Properties on Rheological Properties

In this sub-heading, the interaction of WRAs progressing from WRA8 to WRA19 with cements with varying C_3_A ratios was examined through rheological properties.

When the results were examined, it was observed that increasing the phosphate and sulfonate content of the WRAs up to a certain point improved the rheological characteristics of the mixtures, irrespective of the cement C_3_A ratio. In paste mixtures prepared with WRA12 (9% phosphate content) and WRA17 (7% sulfonate content) at a 0.32 W/C ratio, a reduction in viscosity and dynamic yield stress compared to the paste containing the WRA1 (100% Carboxylate). For mixtures with a 0.35 W/C ratio, a similar trend was observed.

The impact of the type of anionic group on the rheological properties of the paste was distinct from its effect on adsorption. Several factors contribute to this difference. Notably, phosphate and sulfonate groups have a greater ability to form chelate complexes with Ca^2+^ ions on cement particles compared to carboxylates. It has been suggested that phosphate and sulfonate form stronger bonds with Ca^2+^ [61]. As a result, increasing the proportion of phosphate and sulfonate anionic groups, up to a certain level, enhances the rheological properties of the paste. However, beyond a specific substitution ratio, while the adsorption of the WRA increases, the rheological properties of the paste deteriorate (i.e., viscosity and dynamic yield stress increase). This is because a higher adsorption affinity, due to increased phosphate and sulfonate substitution, leads to more WRA being adsorbed on other cement grains. Excessive adsorption, however, can cause bridging between adjacent cement grains, leading to agglomeration [62].

#### 3.1.3. Effect of WRA Anionic Charge Densities on Rheological Properties

In this subheading, the interaction of WRAs progressing from WRA20 to WRA25 with cements with varying C_3_A ratios was examined through rheological properties.

Regardless of the cement type, WRAs with an anionic charge density of 2.3:1 and 4:1 improved the rheological properties of paste mixtures with 0.32 and 0.35 W/C ratios compared to those with an anionic charge density of 3:1. However, an increase in the anionic charge density to 4:1 led to lower viscosity and dynamic yield stress (DYS) values compared to a reduction in anionic charge density to 2.3:1. The effect of increasing charge density became more pronounced as the WRA dosage was raised from 0.10% to 0.15%.

The adsorption of WRA on cement increases with a higher anionic charge density, leading to a greater dispersing effect. Thus, raising the WRA’s anionic charge density enhanced the viscosity and DYS of the paste mixtures [38,63]. However, even with low adsorption, the rheological properties improved when the WRA anionic charge density was below a certain threshold. In such cases, the molecular conformation of the polymer appears to play a critical role in its effectiveness [64,65].

Additionally, increasing the WRA’s anionic group density can sometimes lead to shrinkage or disruption due to the rigidity of the polymer’s main chain. This can reduce the hydrodynamic diameter (Rh), which defines the effective area of action of the WRA, thus decreasing its dispersion performance [66]. Nevertheless, in the WRAs used in this study, the increase in adsorption due to higher anionic group content generally outweighed the decrease in adsorption caused by shrinkage. Consequently, the R-4:1, P9-4:1, and S5-4:1 admixtures outperformed the other WRAs.

### 3.2. Evaluation of Modeling Results

This section compares the findings of the machine learning techniques used in this study. This section consists of three subsections. In the first subsection, the results obtained by using the ANN hyperparameter optimization (*op*ANN) procedure proposed in this study using 10 different metaheuristic algorithms, are compared, and the most successful metaheuristic algorithm and the best *op*ANN models for dynamic yield stress and final viscosity prediction are identified. In the second subsection, the performance of the best *op*ANN model and other competing machine learning methods on dynamic yield stress and final viscosity models is analyzed. In the third and last subsection, the constraints of the obtained models are presented.

#### 3.2.1. Proposed *op*ANN Modeling Results

In this section, the findings from ANN hyperparameter optimization using various metaheuristic algorithms are utilized to identify the most robust algorithms for efficient hyperparameter optimization. Subsequently, the best ANNs for predicting dynamic yield stress and final viscosity are selected, and their network architectures are presented.

(i) Comparison of algorithm performances in ANN hyperparameter optimization: The ANN hyperparameter optimization process (*op*ANN) is described in detail in Section 2.3.9. In the optimization process, networks are trained using the Levenberg–Marquardt algorithm coupled with a 5-fold cross-validation strategy. Metaheuristic search algorithms monitor the errors, and the objective function for optimization is strictly defined as the mean squared error (MSE) of the validation data. To prevent data leakage, the test dataset was completely isolated and never exposed to the network during the training or hyperparameter optimization phases.

Ten different metaheuristic search algorithms were used in the optimization process. The aim is to select the most appropriate algorithm for modeling the data used in the study. Friedman test was used for this purpose. The Friedman test is applied to error values obtained from independent simulations to assess the effectiveness of metaheuristic search algorithms in determining the optimal network structure. This ranks the algorithms based on their success. The Friedman test scores and the *p*-values obtained from this test for the metaheuristic search algorithms are presented in Table 14.

**Figure 5:**
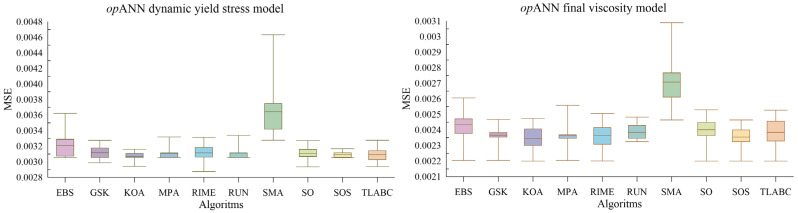
Box plots according to the statistics of the findings obtained from ANN hyperparameter optimization with metaheuristic algorithms.

Upon examining Table 14, it is observed that the *p*-values obtained from the Friedman test for both models are less than the 0.05 significance level (*p* < 0.05). This proves that the differences in the predictive performances of the algorithms are not coincidental and are statistically significant. Analyzing the scores on this statistical basis; in the dynamic yield stress prediction models, the KOA algorithm performs best in ANN hyperparameter optimization. Also, in the viscosity prediction models, the KOA algorithm performs best in ANN hyperparameter optimization. The second algorithm with the highest performance in the dynamic yield stress prediction model was GSK, while the third algorithm was RIME. In the final viscosity prediction model, the second algorithm was TLABC while the third algorithm was GSK. According to the average Friedman score of both models, the top three algorithms are KOA, GSK, and RUN, respectively. This clearly shows, as supported by the statistical differences, that the KOA algorithm is the algorithm with the highest performance in the ANN hyperparameter optimization process (*op*ANN). Box plots of the objective function statistics (MSE) of all algorithms from independent runs are shown in Figure 5.

Figure 5 shows that in the dynamic yield stress prediction model, the KOA algorithm in ANN hyperparameter optimization achieved the minimum average MSE value. Also in the viscosity prediction models, the KOA algorithm achieved the minimum average MSE value. These results confirm the evaluation based on Friedman scores.

Furthermore, the Wilcoxon test was applied to detail the statistical significance of the performance differences between the algorithms, and the results are summarized in Table 15. The three scores (+/=/−) provided in each cell in Table 15 represent the number of cases for which the competing algorithms perform better, similar, or worse than the KOA algorithm, respectively. For instance, according to the Σ 0/7/2 score obtained for the dynamic yield stress model, none of the 9 competing algorithms outperformed the KOA algorithm; 7 algorithms exhibited statistically similar performance, while 2 algorithms performed worse than KOA. The Σ 0/5/4 score obtained for the final viscosity model similarly confirms statistically that no competitor could surpass KOA. To summarize, the most successful algorithm for hyperparameter optimization in this problem is the KOA algorithm. Nevertheless, it cannot be guaranteed that the algorithm will perform identically for different data sets. This phenomenon is attributed to the “no free lunch” theorem [67]. This theorem posits that no single algorithm can successfully address every optimization problem. Consequently, when dealing with diverse datasets, the performance of multiple algorithms must be evaluated on the pertinent dataset to identify the most effective one for a specific problem.

When all these findings are evaluated comprehensively; the 5-fold cross-validation strategy and the isolated test dataset approach, which strictly prevent data leakage during network training, fully guarantee the reliability of the optimization results obtained. Built upon this robust methodological foundation, both the multiple (Friedman, *p* < 0.05) and pairwise (Wilcoxon) statistical tests have proven that the KOA algorithm either outperformed or maintained statistical superiority over the other 9 contemporary metaheuristic algorithms in predicting both rheological parameters. Consequently, it is definitively established that KOA is the most successful and stable algorithm for the hyperparameter optimization (*op*ANN) process in this study. Nevertheless, this superior success does not imply that the algorithm will exhibit the exact same performance on different datasets. This phenomenon is attributed to the “no free lunch” theorem [67], which posits that no single algorithm can successfully solve every optimization problem. As a result, when dealing with diverse engineering and materials science problems, the problem-specific performance of multiple algorithms must always be independently tested to identify the most effective tool.

(ii) Selection of the best ANNs for the prediction models: At this stage, the networks with the smallest error for all experimental data from 21 independent simulations were selected in the hyperparameter optimization performed with metaheuristic search algorithms.

**Figure 6:**
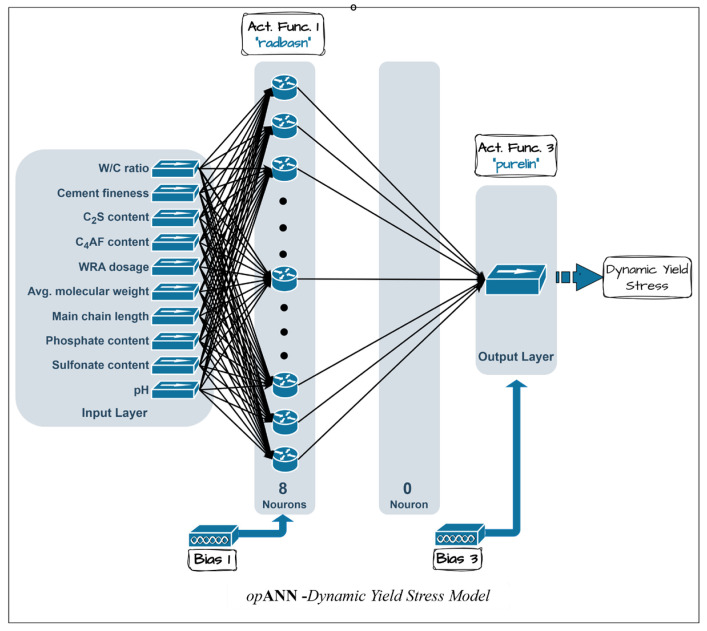
Best ANN architectures determined by *op*ANN procedure for the dynamic yield stress model.

**Figure 7:**
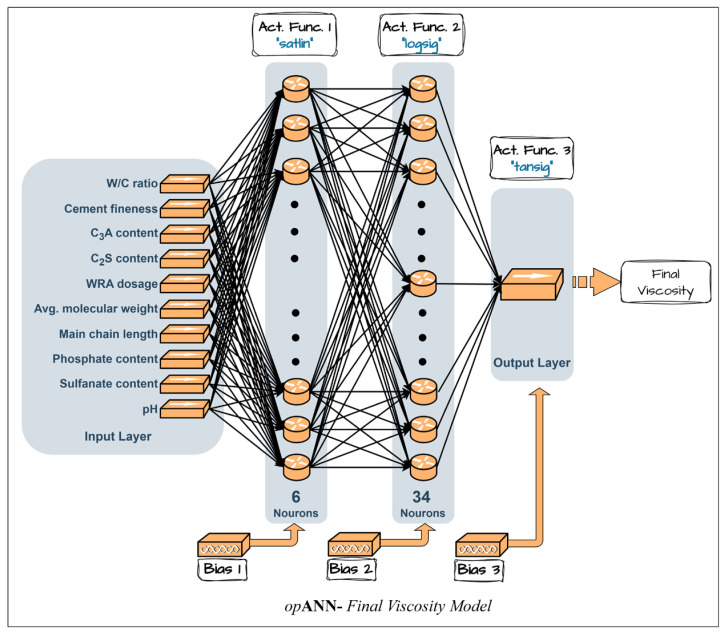
Best ANN architectures determined by *op*ANN procedures for the final viscosity model.

The optimum networks for the dynamic yield stress and final viscosity prediction models were determined based on the minimum mean squared errors (MSE) of the normalized validation dataset, achieving values of 0.0029 and 0.0022, respectively. The corresponding optimal network architectures are presented in Figure 6 and Figure 7.

#### 3.2.2. Results of Competing Regression and Machine Learning Methods

In this subsection, the performance of regression and machine learning techniques is investigated for models predicting dynamic yield stress and final viscosity. These models were chosen as competitors to the *op*ANN procedure. The input parameters for these models are shown in Figure 3.

Following the definition of the input variables, a rigorous hyperparameter tuning process was executed for each baseline model to ensure a fair and robust comparison with the proposed *op*ANN approach. Based on the grid search protocol and the 5-fold cross-validation strategy detailed in the methodology section, the models were fine-tuned to achieve their maximum predictive capacities. The optimal hyperparameter configurations that yielded the best performance for both the dynamic yield stress and final viscosity prediction models are summarized in Table 16. These optimally configured baseline models were subsequently utilized for the final performance evaluation against the *op*ANN procedure.

The error and performance metrics for the models obtained using all competing methods are presented in Table 17 for the dynamic yield stress prediction model and Table 18 for the final viscosity prediction model.

The evaluated models encompass classical regression analyses (CRA) alongside an array of advanced machine learning algorithms, including Multivariate Adaptive Regression Splines (MARS), Support Vector Machines (SVM), Decision Trees (DT), Random Forest (RF), k-Nearest Neighbors (kNN), Model Tree (M5tree), and the proposed hyperparameter-optimized artificial neural network (*op*ANN). The primary objective is to rigidly evaluate the generalization capabilities of these models on the isolated test dataset and to identify the most robust predictive framework.

The performance evaluation is based on the examination of the Root Mean Square Error (RMSE), Nash–Sutcliffe Efficiency (NSE), and Performance Index (PI) metrics. Table 17 and Table 18 detail these test dataset metrics for the dynamic yield stress and final viscosity prediction models, respectively. As established in the methodology, a model characterized by lower RMSE and PI values, coupled with a higher NSE value, is mathematically considered to possess superior predictive accuracy.

An initial analysis of the classical regression functions reveals that the quadratic function (CRA-QF) is the most successful traditional approach. It achieved the highest accuracy among the CRA methods for both dynamic yield stress (RMSE = 16.96, NSE = 0.795, PI = 0.19) and final viscosity (RMSE = 1.63, NSE = 0.843, PI = 0.12). However, a substantial enhancement in predictive capability is observed when employing machine learning techniques. Among the standalone machine learning models, M5tree demonstrated the best performance for the dynamic yield stress model (RMSE = 12.18, NSE = 0.894, PI = 0.12). Conversely, for the final viscosity predictions, the MARS algorithm slightly outperformed the other standard ML techniques, achieving the lowest error margins (RMSE = 1.38, NSE = 0.888, PI = 0.10).

**Figure 8:**
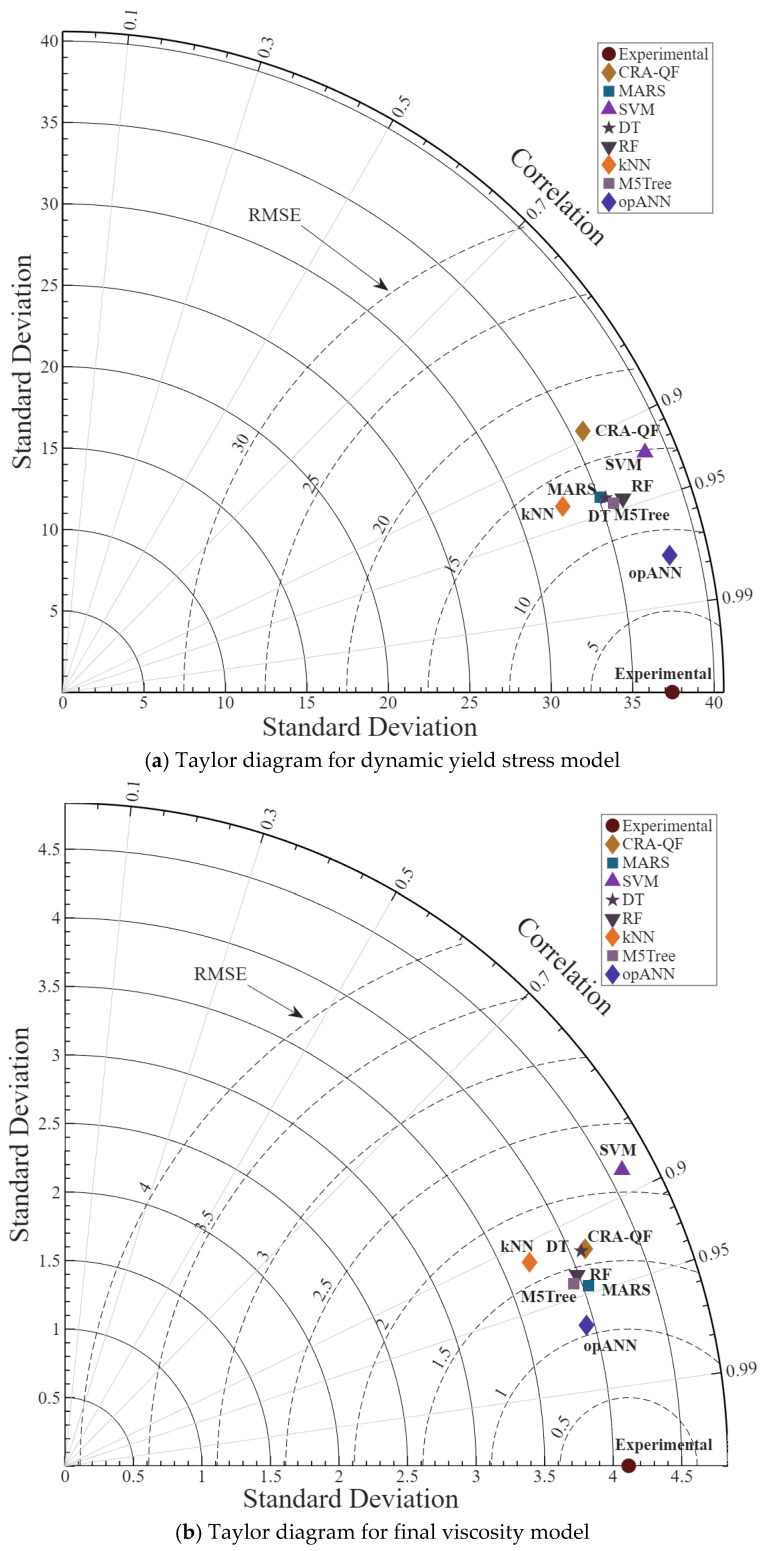
Taylor diagrams comparing competing machine learning methods for dynamic yield stress and final viscosity models.

Despite the competitive results yielded by advanced tree-based and spline models, the proposed *op*ANN architecture decisively outperformed all competing methods across both rheological parameters. For the dynamic yield stress predictions, the *op*ANN model minimized the RMSE to 8.56 and the PI to an exceptional 0.09, while maximizing the NSE to 0.948. Similarly, in the final viscosity predictions, the *op*ANN model achieved the highest overall performance with an RMSE of 1.12, an NSE of 0.925, and a PI of 0.08. To further guide the visual evaluation and corroborate the comparative superiority of the *op*ANN method over all competing machine learning algorithms, Taylor diagrams are presented in Figure 8.

A Taylor diagram is a graphical tool used to assess the agreement between model simulations and experimental data. This diagram visualizes model performance with various statistical metrics (correlation, standard deviation and root mean square error). The axis of the Taylor diagram shows the standard deviation of the experimental data and the Y-axis shows the standard deviation of the model simulations. The radial axis of the graph, i.e., the lines running from the center outwards, shows the correlation coefficient (R). Correlation measures the agreement between model predictions and experimental data. In the Taylor diagram, RMSE indicates how close the model is to the experimental data. The smaller the RMSE value, the better the model is.

In light of all this information, it is seen that the highest correlation between prediction and experimental data for dynamic yield stress and final viscosity models is obtained with the *op*ANN method. When evaluated by standard deviation, the *op*ANN method yields the closest match between the models and the experimental data. Similarly, the smallest RMSE value was also achieved with the *op*ANN method. It is also noteworthy that the models with the closest performance to the *op*ANN method are m5Tree and MARS.

**Figure 9:**
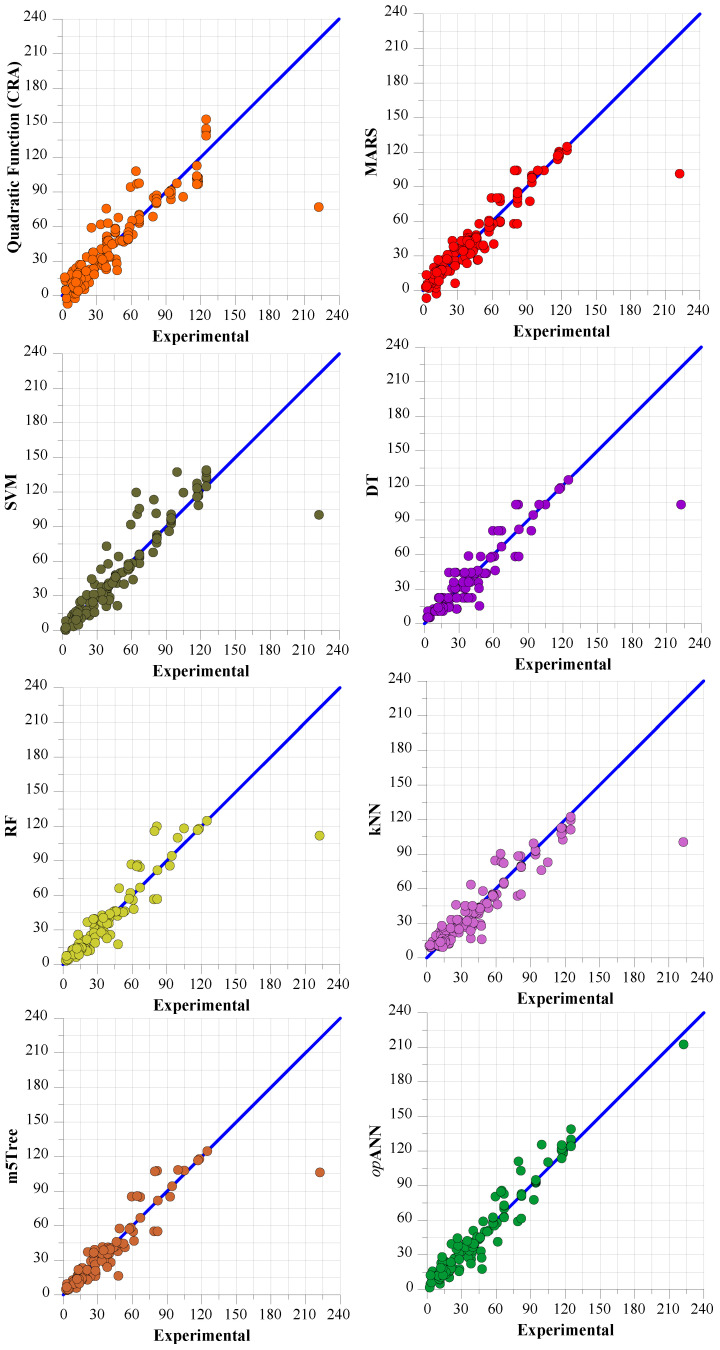
Scatter plots of experimental dynamic flow stress results with test data prediction results obtained from CRA-QF, MARS, SVM, DT, RF, kNN, m5Tree, and *op*ANN.

**Figure 10:**
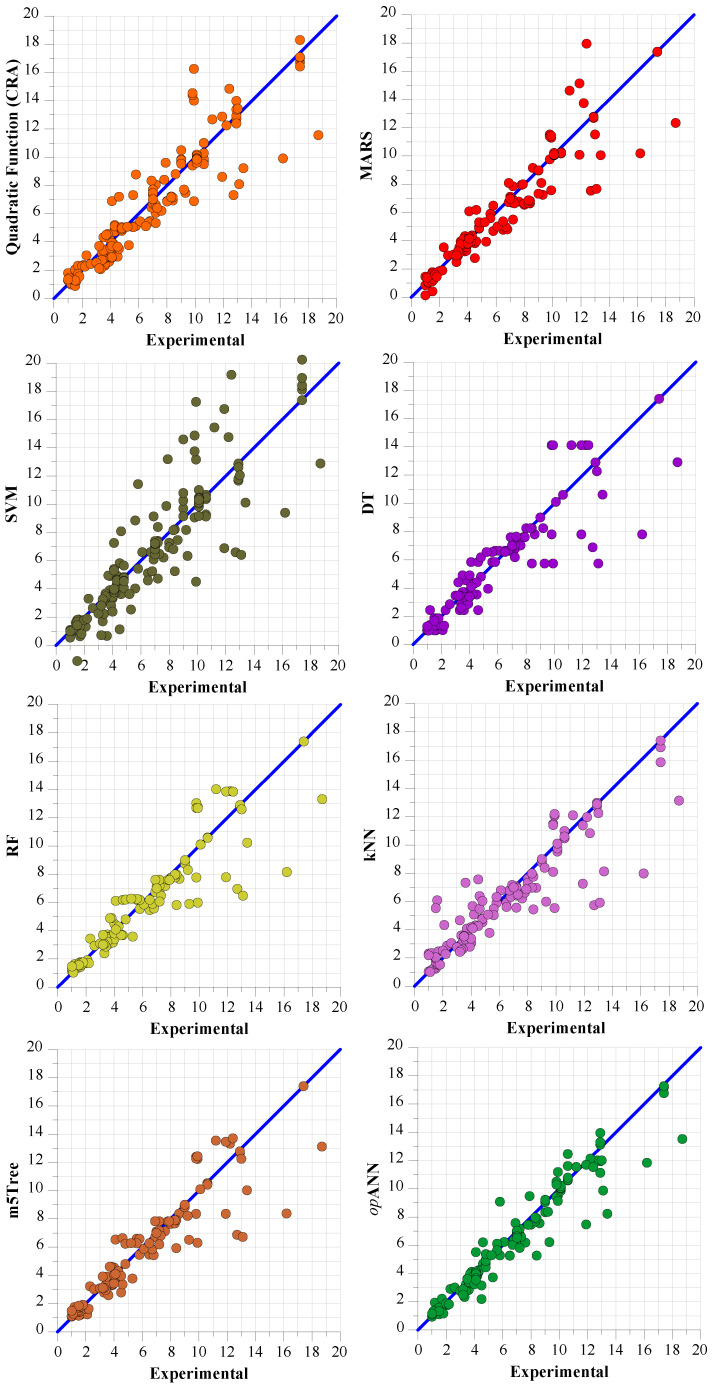
Scatter plots of experimental final viscosity results with test data prediction results obtained from CRA-QF, MARS, SVM, DT, RF, kNN, m5Tree, and *op*ANN.

Figure 9 and Figure 10 present scatter plots illustrating the performance of the models for dynamic yield stress and ultimate viscosity predictions, respectively, against the test data.

When analyzing the error values presented in Table 17 and Table 18 alongside the scatter diagrams in Figure 9 and Figure 10, it becomes apparent that the *op*ANN model for dynamic yield stress prediction and final viscosity prediction exhibits a much tighter clustering around the zero-error line. This visual representation underscores that these models deliver the most robust positive correlation between the predicted values and the experimental data. However, the graphs confirm that the m5Tree and MARS models have the closest prediction performance to the *op*ANN model, similar to the findings obtained in the Taylor diagram.

**Figure 11:**
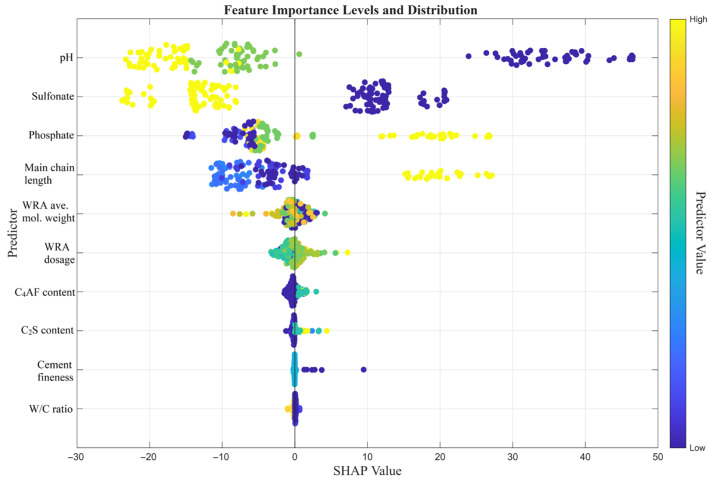
Feature importance and SHAP value distribution for the dynamic yield stress model.

**Figure 12:**
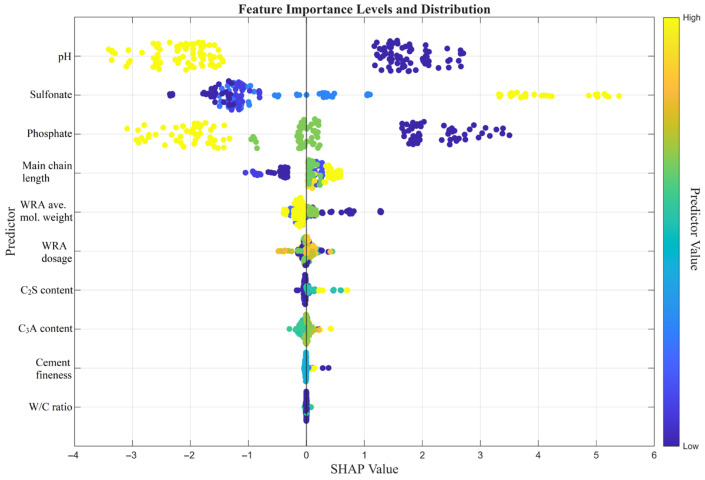
Feature importance and SHAP value distribution for the final viscosity model.

To enhance transparency into the ‘black-box’ nature of the developed machine learning models and to evaluate the marginal effects of input parameters on predictive outcomes, a SHAP (Shapley Additive Explanations) analysis was conducted. The SHAP summary plots, illustrating the feature importance rankings and impact directions for the dynamic yield stress and final viscosity models, are presented in Figure 11 and Figure 12.

An analysis of the SHAP summary plot presented in Figure 11 for the Dynamic Yield Stress model reveals that the most critical factors governing the yield stress are pH, Sulfonate, Phosphate, and Main chain length, respectively. The most decisive feature in the model is the effect of pH. Low pH levels dramatically increase the yield stress, elevating the SHAP value to a range of +40 to +50. Conversely, high pH values play a mitigating role in the yield stress. A high sulfonate content generates negative SHAP values ranging from −10 to −20, thereby reducing the yield point of the system and facilitating fluidity. In contrast, a high phosphate content leads to positive SHAP values, which in turn increases the yield stress. Physical parameters traditionally expected to dominate rheology, such as cement composition (C_4_AF, C_2_S), cement fineness, and even the Water/Cement (W/C) ratio, are clustered in a very narrow band (an ineffective zone) around the SHAP zero line.

Examining the SHAP plot provided in Figure 12 for the Final Viscosity model, it is evident that the effect hierarchy of the variables exhibits almost the exact same pattern as the yield stress model. Among the model input parameters, pH, Sulfonate, Phosphate, and Main chain length once again occupy the top four positions. However, the scale of the SHAP values in this case (ranging from −3 to +6) has narrowed in accordance with the physical limits of the predicted viscosity unit. Low pH, along with high phosphate content and extended main chain length, contribute to an increase in viscosity, whereas high sulfonate ratios decrease it. Unlike the yield stress model, the C_2_S content in the viscosity model ranks one step above C_3_A, demonstrating a relatively more distinct—albeit still limited—differentiation.

In summary, the SHAP analysis—conducted to enhance the transparency of the decision-making processes of the machine learning models—demonstrated that the chemical structure of polycarboxylate-based water-reducing admixtures (WRAs) plays a substantially more dominant role in rheology compared to the physical properties of the cement, for both the dynamic yield stress and final viscosity models. For both rheological parameters (Dynamic Yield Stress and Final Viscosity), pH, Sulfonate, Phosphate, and Main chain length emerged as the most critical (top-4) variables. It was observed that low pH levels (indicated by blue dots) radically increase both the yield stress (up to SHAP values of +50) and the viscosity. When evaluating the effects of the functional groups within the admixture architecture, it was determined that Sulfonate and Phosphate groups operate via opposing mechanisms on the rheology. A high sulfonate content (yellow dots) generated negative SHAP values, thereby reducing both rheological resistances. In contrast, a high phosphate ratio and an increased main chain length led to an increase in both yield stress and viscosity.

#### 3.2.3. Modelling Limitations

In this subsection, the effective operational ranges of the models developed in the study are outlined, and their limitations are defined. While the performance of the ANN models created in this study is commendable, it is important to note that these models were trained on a limited dataset. Consequently, Table 19 and Table 20 provide a summary of the usage limits for dynamic yield stress and final viscosity models, respectively.

### 3.3. Practical Engineering Implications and Model Limitations

The developed models can be primarily utilized for the following practical purposes:

Mixture Design Optimization Scenario: Consider a concrete batching plant that needs to select the most suitable water-reducing admixture (WRA) type and dosage for a specific cement with a given composition and fineness (e.g., CEM I). In this scenario, instead of synthesizing 25 different WRAs and conducting 750 physical experiments, the practitioner can input the physical and chemical data of the available cement and potential WRAs (such as pH, molecular weight, sulfonate content, etc.) directly into the proposed *op*ANN model. Consequently, the model can predict and determine the most economical WRA dosage required to achieve the targeted dynamic yield stress and final viscosity values.

New Admixture Development (R&D) Scenario: Assume a chemical admixture manufacturer aims to synthesize a novel polycarboxylate ether (PCE) designed to provide lower viscosity. Before initiating any laboratory synthesis, the manufacturer can conduct “virtual trials” using the model by systematically adjusting input parameters such as molecular weight, main chain length, phosphate and sulfonate contents, and pH. Ultimately, the model can proactively predict which molecular architecture will yield the optimal rheological performance, thereby significantly reducing the costs and time associated with empirical R&D cycles.

Through these approaches, there is substantial potential for time and cost savings, particularly for concrete batching plants and chemical admixture R&D centers. However, it must be strongly emphasized that the reliability of these practical applications is strictly confined within the specific interpolation boundaries of the training dataset utilized in this study. Therefore, any real-world utilization of the proposed models must strictly operate within the input parameter ranges explicitly detailed in Table 19 and Table 20.

## 4. Conclusions

This study successfully established an integrated, data-driven workflow where targeted chemical synthesis and systematic rheological experiments were explicitly utilized to construct a micro-level, high-quality dataset. This robust data infrastructure enabled the development of highly accurate ML-based predictive models for cement-WRA interactions. The key findings from the study are summarized below:

- From the experimental perspective, increasing the W/C ratio from 0.32 to 0.35 consistently reduced both DYS and FV, confirming the dominant role of interparticle spacing in mitigating flocculation intensity. Among the chain-length-modified WRAs, the polymer possessing balanced main and side chain lengths (WRA1) exhibited superior compatibility across cements with varying C_3_A contents. Deviations from this molecular balance, either through shortening or excessive elongation of main or side chains, resulted in increased viscosity and yield stress, indicating reduced steric stabilization efficiency.
- Regarding anionic group chemistry, partial substitution of carboxylate groups with phosphate (9%) or sulfonate (7%) significantly improved dispersion performance at both W/C ratios. However, beyond an optimal substitution threshold, excessive adsorption affinity led to rheological deterioration, likely due to interparticle bridging. Similarly, increasing anionic charge density to 4:1 enhanced dispersion performance compared to 3:1 systems, particularly at higher dosages (0.15%), confirming the dominant role of electrostatic repulsion when steric constraints were appropriately balanced.
- These experimentally observed non-linear structure, property relationships created a complex, multidimensional interaction landscape between polymer architecture, C_3_A content, and rheological response. Leveraging this high-resolution dataset, highly accurate ML-based predictive models were developed.
- Evaluations conducted strictly on the isolated test dataset, ensuring zero data leakage, demonstrated that the proposed *op*ANN architecture decisively outperformed all competing classical regression and standalone ML models. Specifically, for dynamic yield stress (DYS), the *op*ANN model achieved the highest predictive accuracy (RMSE = 8.56, NSE = 0.948, PI = 0.09), significantly surpassing the second-best model, M5Tree (RMSE = 12.18, NSE = 0.894). Similarly, for final viscosity (FV), *op*ANN yielded the lowest error metrics (RMSE = 1.12, NSE = 0.925), outperforming the second-best MARS algorithm (RMSE = 1.38, NSE = 0.888). Among the traditional regression equations, the quadratic function (QF) provided the best baseline but remained substantially inferior to the ML approaches.
- The efficacy of contemporary metaheuristic algorithms in optimizing the ANN hyperparameter architecture was statistically validated. The Friedman test (*p* < 0.05) confirmed significant performance disparities, with the KOA algorithm demonstrating the highest optimization capability. Post hoc Wilcoxon signed-rank tests further solidified KOA’s superiority, revealing that no competing algorithm outperformed KOA in any independent runs for either the DYS (0/7/2) or FV (0/5/4) models.
- In accordance with the No Free Lunch (NFL) theorem [67], the exceptional optimization performance of the KOA algorithm is strictly confined to the specific boundaries of the 750-point experimental dataset and the hyperparameter search space defined in this study. While KOA proves to be a highly effective tool for the complex rheological behavior of this specific cement–WRA system, its performance cannot be automatically generalized to different modeling problems.
- Crucially, the SHAP interpretability analysis successfully bridged the gap between empirical laboratory observations and algorithmic predictions. The feature importance patterns extracted via SHAP corroborated the experimentally observed physicochemical mechanisms, quantitatively confirming the critical rheological impacts of the phosphate and sulfonate groups, thus proving the model learned underlying chemical interactions rather than merely memorizing data.

Limitations and Future Work

Despite the robust predictive performance demonstrated in this study, several limitations must be explicitly acknowledged to ensure proper interpretation of the results. First, the developed models—including the optimized KOA-ANN architecture—are strictly bounded within the interpolation domain of the experimental dataset. The dataset comprises 750 formulations generated using a single cement type (CEM I 42.5R), specific C_3_A ranges, controlled laboratory temperature (20 ± 1 °C), and defined W/C and admixture dosage intervals. Therefore, extrapolation beyond these parametric boundaries (e.g., different clinker mineralogy, altered sulfate balance, or substantially different alkali contents) may introduce uncertainty. Second, the models were trained exclusively on systems without supplementary cementitious materials (SCMs). The presence of mineral additives such as fly ash, slag, limestone filler, or calcined clay may significantly alter ionic strength, surface chemistry, adsorption competition, and early-age hydration kinetics. Since PCE adsorption behavior is highly sensitive to pore solution chemistry and solid surface characteristics, model generalization to blended or low-clinker systems cannot be assumed without independent validation. Third, temperature effects were not explicitly incorporated into the modelling framework. Given that both hydration kinetics and polymer conformation in solution are temperature-dependent, rheological behavior under elevated or reduced curing temperatures may deviate from the patterns captured in the present dataset. Additionally, the experiments were conducted under controlled laboratory-scale paste conditions. Scaling the framework to concrete-level systems introduces further complexity, including aggregate surface interactions, mixing energy variations, and shear-history differences typical of batching plant operations. In accordance with the No Free Lunch theorem, the optimization superiority of the KOA algorithm is inherently problem-specific and cannot be automatically generalized to unrelated modelling domains or expanded chemical spaces. Future work should focus on independent external validation utilizing diverse cementitious systems, such as blended cements or calcined clays. Furthermore, scaling this integrated workflow to real-world concrete batching plant scenarios and continuously incorporating new empirical data will be essential to refine and generalize the predictive framework.

## Figures and Tables

**Table 1:** Recent studies on the modeling of cementitious systems using ML techniques.

Reference	Estimated Dependent Parameter	Year	Used ML Technique	Has the ML Technique Been Improved with the Metaheuristic Algorithm?	Which Metaheuristic Algorithms Were Used to Improve the Results?
[14]	Carbonation depth	2025	MGGP, RF, ANN, GRA	Feature selection	PESA-II (2004)
[20]	Compressive strength	2024	GEP, ANN, RF, SVM, ANFIS, OGPR, GB, XGB, AdaBoost, KNN, BR	no	-
[16]	Yield stress, plastic viscosity, adsorption ratio of superplasticizer, bleeding	2023	ANN	no	-
[13]	Channel flow or bleeding rate	2023	ANN	no	-
[22]	Compressive strength, splitting tensile strength	2025	GPR, PF, DT	no	-
[26]	Flexural strength	2025	SVM, RF, XGB, MLP	no	-
[23]	Compressive strength	2025	KNN, RF, CatBoost	no	-
[24]	Compressive strength	2025	XGB, DT, SVM, KNN, BGR, AdaBoost, GBR, LR	no	-
[25]	Compressive strength	2025	XGB, GB, SVR, LR, DT, KNN, AdaBoost	no	-
[26]	Compressive strength, splitting tensile strength	2024	RF, GP	no	-
[28]	Compressive strength	2025	XGB, RF, SVM, DT, KNN, LR, Ridge, Lasso	no	-
[12]	Marsh cone time	2022	ANN	no	-
[18]	Compressive strength	2025	ET, XGB, GB, RF, DT, LGB, KNN, AdaBoost, ANN, SVM, LR, Ridge, Lasso, LLAR	SVM hyperparameter optimization	GA (1989)
[15]	Compressive strength, tensile strength	2024	ANN, ANFIS, GEP	no	-
[17]	Compressive strength of cement-grouted sand	2021	CRA	no	-

**Table 2:** Chemical, physical, and mechanical properties of cements.

		Type of Cement				
Chemical Components	Unit	C2	C3	C6	C9	CEM I
SiO_2_	%	18.94	19.58	19.73	20.15	18.74
Al_2_O_3_	%	4.33	4.72	5.19	5.53	5.37
Fe_2_O_3_	%	5.53	5.27	4.10	3.31	3.04
CaO	%	61.67	60.62	62.62	62.68	64.11
MgO	%	1.55	1.77	1.75	1.69	1.21
SO_3_	%	2.82	2.66	2.36	3.10	2.68
Na_2_O	%	0.31	0.32	0.36	0.26	0.34
K_2_O	%	0.57	0.54	0.57	0.58	0.62
Cl^−^	%	0.0375	0.0417	0.0436	0.0495	0.038
Free CaO	%	0.75	0.86	1.11	0.70	2.12
Loss of ignition	%	3.33	3.34	3.01	4.31	3.60
C_3_A	%	2.13	3.60	6.82	9.05	9.10
C_3_S	%	58.98	47.60	52.96	48.42	64.5
C_2_S	%	9.80	20.22	16.61	21.25	-
C_4_AF	%	16.83	16.04	12.48	10.07	9.3

**Table 3:** Properties of synthesized WRAs.

WRA Type	Mw ^1^	Mn ^2^	Main Chain Length (k)	Side-Chain Length (g/mole)	Anionic Charge Density ^3^	Carboxylate	Phosphate	Sulfonate	pH
WRA1	56,000	26,200	21	2400	3	100	0	0	3.97
WRA2	27,000	11,740	10	2400	3	100	0	0	2.87
WRA3	78,000	37,140	31	2400	3	100	0	0	3.26
WRA4	26,000	11,300	21	1000	3	100	0	0	3.16
WRA5	69,000	34,500	21	3000	3	100	0	0	3.44
WRA6	56,000	24,340	40	1000	3	100	0	0	3.27
WRA7	57,000	27,140	17	3000	3	100	0	0	3.27
WRA8	58,000	26,300	21	2400	3	99	1	0	2.91
WRA9	65,000	27,000	21	2400	3	97	3	0	2.93
WRA10	61,000	26,500	21	2400	3	95	5	0	2.93
WRA11	56,100	26,600	21	2400	3	93	7	0	2.65
WRA12	53,000	23,000	21	2400	3	91	9	0	2.54
WRA13	52,000	22,600	21	2400	3	80	20	0	2.61
WRA14	62,100	29,500	21	2400	3	99	0	1	1.08
WRA15	63,000	28,600	21	2400	3	97	0	3	1.08
WRA16	58,000	29,000	21	2400	3	95	0	5	1.08
WRA17	63,000	28,600	21	2400	3	93	0	7	1.08
WRA18	60,000	25,000	21	2400	3	91	0	9	1.08
WRA19	61,000	29,000	21	2400	3	80	0	20	1.08
WRA20	63,000	30,000	21	2400	2.3	100	0	0	3.54
WRA21	56,000	26,500	21	2400	4	100	0	0	2.94
WRA22	60,000	26,000	21	2400	2.3	91	9	0	2.97
WRA23	54,000	22,500	21	2400	4	91	9	0	3.21
WRA24	62,000	29,000	21	2400	2.3	91	0	9	3.24
WRA25	56,000	28,000	21	2400	4	91	0	9	3.58

^1^ Weight average molecular weight ^2^ Number average molecular weight. ^3^ Molar ratio of acrilic acid to non-inonic groups.

**Table 4:** Tolerance and VIF values for input parameters of the yield stress model.

All Experimental Inputs			Reduced Inputs After Collinearity Analysis		
	Tolerance	VIF		Tolerance	VIF
W/C ratio	0.996	1004	W/C ratio	0.996	1004
Cement fineness	0.473	2115	Cement fineness	0.553	1808
C_3_A content	2.539 × 10^−7^	3,716,140.681	C_2_S content	0.573	1745
C_3_S content	1.502 × 10^−5^	66,575.225	C_4_AF content	0.893	1120
C_2_S content	0.259	3857	WRA dosages	0.994	1007
C_4_AF content	0.334	2991	Mw (weight),	0.794	1260
Equivalent alkali value	0.160	6248	Main chain length	0.802	1248
WRA dosages	0.993	1007	Phosphate content	0.914	1094
Mw (weight),	0.050	20,013	Sulfonate content	0.679	1473
Mn (number)	0.047	21,156	pH	0.723	1383
Main chain length	0.110	9069			
Side chain length	0.098	10,225			
Carboxylate content	Very small	Inf			
Phosphate content	0.653	1531			
Sulfonate content	0.657	1523			
pH	0.657	1523			

**Table 5:** Tolerance and VIF values for input parameters of the final viscosity model.

All Experimental Inputs			Reduced Inputs After Collinearity Analysis		
	Tolerance	VIF		Tolerance	VIF
W/C ratio	1.000	1.000	W/C ratio	1.000	1.000
Cement fineness	0.406	2.463	Cement fineness	0.589	1.699
C_3_A content	0.014	70.462	C_3_A content	0.933	1.072
C_3_S content	0.000	2197.045	C_2_S content	0.622	1.607
C_2_S content	0.001	1944.626	WRA dosages	1.000	1.000
C_4_AF content	Very small	Inf	Mw (weight)	0.794	1.259
Equivalent alkali value	0.070	14.324	Main chain length	0.803	1.246
WRA dosages	0.886	1.129	Phosphate content	0.912	1.096
Mw (weight),	0.051	19.482	Sulfonate content	0.680	1.470
Mn (number)	0.048	20.667	pH	0.725	1.380
Main chain length	0.112	8.936			
Side chain length	0.099	10.091			
Carboxylate content	Very small	Inf			
Phosphate content	0.650	1.539			
Sulfonate content	0.658	1.520			
pH	0.659	1.518			

**Table 6:** Hyperparameter search space and grid search configurations for the MARS model.

Hyperparameter	Description	Search Space
maxFuncs	The maximum number of basis functions included in model in the forward building phase	{5, 10, 20, 40, 60, 80, 100}
cubic	Whether to use piecewise-cubic or piecewise linear type of modelling	{true, false}
maxInteractions	The maximum degree of interactions between input variables	{1, 2, 4, 6, 8, 10}
prune	Whether to perform model pruning	{true, false}
useMinSpan	a hyperparameter that restricts knot proximity to reduce local variance and prevent overfitting to sequential error patterns.	{0, 2, 5, 10}
useEndSpan	A coefficient used to reduce the local variance of estimates near the endpoints of data ranges.	{0, 10, 20, 30}
newVarPenalty	Penalty for adding a new variable to a model in the forward phase.	{0, 0.01, 0.05, 0.1, 0.2}

**Table 7:** Hyperparameter search space and grid search configurations for the SVM model.

Hyperparameter	Description	Search Space
Kernel Function	Kernel Function	{‘linear’, ‘gaussian’, ‘polynomial’}
BoxConstraint	Box constraint	{0.001, 0.01, 0.1, 1, 10, 100, 200}
ε	Half the width of the epsilon-insensitive band	{0.0001, 0.001, 0.01, 0.1, 1}
γ	Gamma for kernel scale	{0.001, 0.01, 0.1, 1, 10}
PolynomialOrder	Polynomial kernel function order	{1, 2, 3, 4, 5}

**Table 8:** Hyperparameter search space and grid search configurations for the DT model.

Hyperparameter	Description	Search Space
MinParentSize	Minimum number of branch node observations	{1, 2, 5, 10, 20, 50, 100, 200}
MaxNumSplits	Maximal number of decision splits	{0, 1, 3, 5, 10, 20, 30, 50}
MinLeafSize	Minimum number of leaf node observations	{1, 2, 5, 13, 30, 69, 161, 375}
NumVariablesToSample	Number of predictors to select at random for each split	{1, 3, 4, 5, 6, 10}

**Table 9:** Hyperparameter search space and grid search configurations for the RF model.

Hyperparameter	Description	Search Space
NumLearningCycles	Number of ensemble learning cycles	{50, 100, 150, 200, 300, 500}
MinLeafSize	Minimum observations per leaf	{1, 2, 5, 13, 30, 69, 161, 375}
MinParentSize	Minimum observations per branch node	{1, 2, 5, 10, 20, 50}
NumPredictorsSample	Number of predictors to select at random for each split	{1, 3, 4, 5, 6, 10}
MaxNumSplits	Maximal number of decision splits (or branch nodes) per tree	{‘none’, 1, 3, 5, 10, 30}

**Table 10:** Hyperparameter search space and grid search configurations for the kNN model.

Hyperparameter	Description	Search Space
K	Number of nearest neighbors	{1, 3, 5, 7, 9, 11, 13, 17, 21, 25, 35, 41, 51, 81}
BucketSize	Maximum number of data points in the leaf node	{1, 5, 10, 20, 50, 100, 200, 500}
Distance Type	Distance metric	{‘euclidean’, ‘cityblock’, ‘chebychev’, ’minkowski’}
Weight Type	Weight Function	{‘uniform’, ‘inverse’, ‘gaussian’}
Alpha (For inverse)	Exponent for the ’inverse’ weight function	{0.1, 0.25, 0.5, 0.75, 1, 1.5, 2, 3}
Sigma (For gaussian)	Scale Parameter	{0.1, 0.25, 0.5, 0.75, 1, 1.5, 2, 3}
P (for minkowski)	Exponent for the Minkowski distance metric	{1, 1.5, 2, 3, 4, 5, 10}

**Table 11:** Hyperparameter search space and grid search configurations for the M5 Tree model.

Hyperparameter	Description	Search Space
numTrees	Number of trees to build	{50, 100, 200, 250, 300, 500}
minLeafSize	The minimum number of training observations a leaf node may represent	{1, 2, 5, 13, 30, 69, 161, 375}
numVarsTry	Number of input variables randomly sampled as candidates at each split in a tree	{1, 3, 4, 5, 6, 10}
prune	Whether to prune the tree	{‘true’, ‘false’’}
inBagFraction	The fraction of the total number of observations to be sampled for in-bag set	{0.2, 0.3, 0.4, 0.5, 0.6, 0.7, 0.9, 1.0}

**Table 12:** Lower and upper limits of design variables, increment values, and the range of possible values. Reprinted with permission from [42]. Polymers. 2025.

Design Variable	Lower Limit	Upper Limit	Increment	Number of Possible Values
D_1_: Number of neurons in the first hidden layer	2	40	2	20
D_2_: Number of neurons in the second hidden layer	0	40	2	21
D_3_: Activation function in the first hidden layer	{purelin, tansig, logsig, elliotsig, hardlim, hardlims, satlin, satlins, poslin, tribas, radbasi, radbasn}			12
D_4_: Activation function in the second hidden layer				12
D_5_: Output layer activation function				12

**Table 13:** Settings for algorithm parameters. Reprinted with permission from [42]. Polymers. 2025.

Algorithm	Reference	Settings
EBS	[43]	Population size = 10
GSK	[44]	Population size = 100, P = 0.1, $k_{f}$ = 0.5, $k_{r}$ = 0.9, K = 10
KOA	[45]	N = 25, T = 3, μ0 = 0.1, γ = 15
MPA	[46]	Number of search agents = 25, FADs = 0.2, P = 0.5
RIME	[47]	Population size = 30, w = 5
RUN	[23]	Population size = 50
SMA	[48]	Population size = 30, z = 0.03
SO	[49]	Search Agents no = 30, vec_flag = [1, −1], Threshold = 0.25, Threshold2 = 0.6, C1 = 0.5, C2 = 0.05, C3 = 2
SOS	[50]	Ecosystem size = 50
TLABC	[51]	Number of food sources (NP) = 50, limit = 200, Scale factor (F) = rand

**Table 14:** Friedman test scores metaheuristic search algorithms.

	Friedman Scores		
	ANN Models		Mean Score
	Dynamic Yield Stress Model	Final Viscosity Model	
*op*ANN-KOA	3.571	4.619	4.095
*op*ANN-GSK	4.095	4.667	4.381
*op*ANN-RUN	4.762	5.095	4.929
*op*ANN-RIME	4.429	5.952	5.191
*op*ANN-MPA	4.762	5.976	5.369
*op*ANN-SOS	6.429	5.071	5.750
*op*ANN-TLABC	7.191	4.619	5.905
*op*ANN-SO	7.762	4.905	6.333
*op*ANN-EBS	6.714	6.238	6.476
*op*ANN-SMA	5.286	7.857	6.571
*p* value	3.41 × 10^−10^	3.95 × 10^−11^	

Green represents the first algorithm, blue represents the second, and orange represents the third.

**Table 15:** Wilcoxon test scores metaheuristic search algorithms.

	Wilcoxon Test Results	
KOA vs	Dynamic Yield Stress Model	Final Viscosity Model
EBS	0/0/1	0/0/1
GSK	0/1/0	0/1/0
MPA	0/1/0	0/1/0
RIME	0/1/0	0/1/0
RUN	0/1/0	0/0/1
SMA	0/0/1	0/0/1
SO	0/1/0	0/0/1
SOS	0/1/0	0/1/0
TLABC	0/1/0	0/1/0
∑	0/7/2	0/5/4

**Table 16:** Best hyperparameter values identified via grid search for the competing machine learning algorithms.

	Dynamic Yield Stress Model	Final Viscosity Model
MARS	maxFuncs = 60, cubic = true, maxInteractions = 4, prune = true, useMinSpan = 2, useEndSpan = 0, newVarPenalty = 0	maxFuncs = 40, cubic = false, maxInteractions = 4, prune = true, useMinSpan = 5, useEndSpan = 0, newVarPenalty = 0
SVM	Kernel Function = gaussian, BoxConstraint = 100, ε = 0.01, γ = 0.1	Kernel Function = polynomial, BoxConstraint = 0.1, ε = 0.01, γ = 0.1, PolynomialOrder = 3
DT	MinParentSize = 1, MaxNumSplits = 30, MinLeafSize = 5, NumVariablesToSample = 10	MinParentSize = 1, MaxNumSplits = 0, MinLeafSize = 5, NumVariablesToSample = 10
RF	NumLearningCycles = 50, MinLeafSize = 2, MinParentSize = 2, NumPredictorsSample = 10, MaxNumSplits = None	NumLearningCycles = 100, MinLeafSize = 2, MinParentSize = 10, NumPredictorsSample = 10, MaxNumSplits = None
kNN	K = 25, BucketSize = 1, Distance Type = minkowski, Weight Type = gaussian, Sigma = 0.25, P = 1.5	K = 5, BucketSize = 1, Distance Type = minkowski, Weight Type = inverse, Alpha = 1, P = 1.5
M5tree	numTrees = 250, minLeafSize = 5, numVarsTry = 10, prune = true, inBagFraction = 1	numTrees = 250, minLeafSize = 5, numVarsTry = 10, prune = true, inBagFraction = 0.90

**Table 17:** Test dataset performance metrics (RMSE, NSE, and PI) for competing methods in the dynamic yield stress prediction model.

Model	Method	RMSE	NSE	PI
Dynamic Yield Stress	CRA-LF	17.99	0.769	0.20
	CRA-PF	18.32	0.761	0.21
	CRA-EF	17.86	0.772	0.20
	CRA-INVF	18.02	0.768	0.20
	CRA-LnF	17.62	0.778	0.20
	CRA-QF	16.96	0.795	0.19
	CRA-SF	18.42	0.758	0.21
	MARS	12.81	0.883	0.14
	SVM	14.82	0.843	0.16
	kNN	13.40	0.872	0.15
	DT	12.65	0.886	0.14
	RF	12.30	0.892	0.13
	M5tree	12.18	0.894	0.12
	*op*ANN	8.56	0.948	0.09

**Table 18:** Test dataset performance metrics (RMSE, NSE, and PI) for competing methods in the final viscosity prediction model.

Model	Method	RMSE	NSE	PI
Final Viscosity	CRA-LF	2.23	0.706	0.18
	CRA-PF	2.29	0.691	0.18
	CRA-EF	2.17	0.722	0.17
	CRA-INVF	2.55	0.614	0.21
	CRA-LnF	2.37	0.669	0.19
	CRA-QF	1.63	0.843	0.12
	CRA-SF	2.43	0.651	0.20
	MARS	1.38	0.888	0.10
	SVM	2.17	0.723	0.17
	kNN	1.67	0.834	0.13
	DT	1.61	0.846	0.12
	RF	1.46	0.874	0.11
	M5tree	1.41	0.883	0.11
	*op*ANN	1.12	0.925	0.08

**Table 19:** Limits to use the dynamic yield stress model.

	Dynamic Yield Stress Model Input Parameters									
Limit	x_1_	x_2_	x_3_	x_4_	x_5_	x_6_	x_7_	x_8_	x_9_	x_10_
Minimum	0.32	3600	8	9.3	0	26,000	10	0	0	1.08
Maximum	0.35	4259	21.25	16.83	0.15	78,000	40	20	20	3.97

x_1_: W/C ratio, x_2_: Cement fineness, x_3_: C2S content, x_4_: C4AF content, x_5_: WRA dosage, x_6_: WRA average molecular weight, x_7_: Main chain length, x_8_: Phosphate amount, x_9_: Sulfonate amount, x_10_: pH.

**Table 20:** Limits to use the final viscosity model.

	Final Viscosity Model Input Parameters									
Limit	x_1_	x_2_	x_3_	x_4_	x_5_	x_6_	x_7_	x_8_	x_9_	x_10_
Minimum	0.32	3600	2.13	8	0	26,000	10	0	0	1.08
Maximum	0.35	4259	9.1	21.25	0.15	78,000	40	20	20	3.97

x_1_: W/C ratio, x_2_: Cement fineness, x_3_: C2S content, x_4_: C4AF content, x_5_: WRA dosage, x_6_: WRA average molecular weight, x_7_: Main chain length, x_8_: Phosphate amount, x_9_: Sulfonate amount, x_10_: pH.

## Data Availability

The original contributions presented in this study are included in the article/Supplementary Materials. Further inquiries can be directed to the corresponding author.

## Supplementary Materials

The following supporting information can be downloaded at: https://www.mdpi.com/article/10.3390/polym18050656/s1.

## References

[1] Ramyar K., Mardani A., Kobya V. (2023). Polikarboksilat Esaslı Su Azaltıcı Katkı-Çimento Uyumu, Beton 2023 Hazır Beton Kongresi, 8–11 Kasım. https://www.thbb.org/media/833780/hazir_beton_dergisi_makale_polikarboksilat_esasli_su_azaltici_katki_cimento_uyumu_186.pdf.

[2] Mardani-Aghabaglou A., Felekoğlu B., Ramyar K. (2017). Effect of cement C3A content on properties of cementitious systems containing high-range water-reducing admixture. J. Mater. Civ. Eng..

[3] Sha S., Wang M., Shi C., Xiao Y. (2020). Influence of the structures of polycarboxylate superplasticizer on its performance in cement-based materials-A review. Constr. Build. Mater..

[4] Ferrari L., Bernard L., DeYZhner F., Kaufmann J., Winnefeld F., Plank J. (2012). Characterization of Polycarboxylate-Ether Based Superplasticizer on Cement Clinker Surfaces. J. Am. Ceram. Soc..

[5] Karakuzu K., Kobya V., Mardani-Aghabaglou A., Felekoğlu B., Ramyar K. (2021). Adsorption properties of polycarboxylate ether-based high range water reducing admixture on cementitious systems: A review. Constr. Build. Mater..

[6] Kong X.M., Zhang Y.R., Hou S.S. (2013). Study on the rheological properties of portland cement pastes with polycarboxylate superplasticizers. Rheol. Acta.

[7] Ma Y., Sha S., Zhou B., Lei F., Liu Y., Xiao Y., Shi C. (2022). Adsorption and dispersion capability of polycarboxylate-based superplasticizers: A review. J. Sustain. Cem.-Based Mater..

[8] Kobya V., Karakuzu K., Mardani A., Felekoğlu B., Ramyar K. (2023). Combined interaction of WRA chains lengths, C3A and water content in cementitious systems. Constr. Build. Mater..

[9] Kobya V. (2023). Effect of Water Reducing Admıxture Chain Length on Behaviour of Cementitious Systems Having Different C3A Content. Ph.D. Thesis.

[10] Mardani-Aghabaglou A. (2016). Investigation of Cement-Superplasticizer Admixture Compatibility. Ph.D. Thesis.

[11] Mardani-Aghabaglou A., Kankal M., Nacar S., Felekoğlu B., Ramyar K. (2021). Assessment of cement characteristics affecting rheological properties of cement pastes. Neural Comput. Appl..

[12] Ardhira P.J., Lakshmi S., Sreya S., Supraja P., Sathyan D. (2022). A multilayer neural network approach on the effect of superplasticizer family on the flow behaviour of PPC paste. Mater. Today Proc..

[13] Kang I.K., Shin T.Y., Kim J.H. (2023). Observation-informed modeling of artificial neural networks to predict flow and bleeding of cement-based materials. Constr. Build. Mater..

[14] Hosseinnia A., Sichani M.N., Alamdari B.E., Aghelizadeh P., Teimortashlu A. (2025). Machine learning formulation for predicting concrete carbonation depth: A sustainability analysis and optimal mixture design. Structures.

[15] Asif U., Memon S.A., Javed M.F., Kim J. (2024). Predictive modeling and experimental validation for assessing the mechanical properties of cementitious composites made with silica fume and ground granulated blast furnace slag. Buildings.

[16] Yoon J., Kim H., Ju S., Li Z., Pyo S. (2023). Framework for rapid characterization of fresh properties of cementitious materials using point cloud and machine learning. Constr. Build. Mater..

[17] Emad W., Salih A., Kurda R. (2021). Experimental study using ASTM and BS standards and model evaluations to predict the compressive strength of the cement grouted sands modified with polymer. Case Stud. Constr. Mater..

[18] Bentegri H., Rabehi M., Kherfane S., Nahool T.A., Rabehi A., Guermoui M., Alhussan A.A., Khafaga D.S., Eid M.M., El-Kenawy E.S. (2025). Assessment of compressive strength of eco-concrete reinforced using machine learning tools. Sci. Rep..

[19] Öztürk N., Şentürk H.B., Gündoğdu A., Duran C. (2018). Modeling of Co(II) adsorption by artificial bee colony and genetic algorithm. Membr. Water Treat..

[20] Aylas-Paredes B.K., Han T., Neithalath A., Huang J., Goel A., Kumar A., Neithalath N. (2025). Data driven design of ultra high performance concrete prospects and application. Sci. Rep..

[21] Choi J.-H., Kim D., Ko M.-S., Lee D.-E., Wi K., Lee H.-S. (2023). Compressive strength prediction of ternary-blended concrete using deep neural network with tuned hyperparameters. J. Build. Eng..

[22] Fawad M., Alabduljabbar H., Farooq F., Najeh T., Gamil Y., Ahmed B. (2024). Indirect prediction of graphene nanoplatelets-reinforced cementitious composites compressive strength by using machine learning approaches. Sci. Rep..

[23] Kumar R., Karthik S., Kumar A., Tantri A., Shahaji, Sathvik S. (2025). Machine learning approach for predicting the compressive strength of biomedical waste ash in concrete: A sustainability approach. Discov. Mater..

[24] Sobuz M.H.R., Khatun M., Kabbo M.K.I., Sutan N.M. (2025). An explainable machine learning model for encompassing the mechanical strength of polymer-modified concrete. Asian J. Civ. Eng..

[25] Mishra M. (2025). Quantifying compressive strength in limestone powder incorporated concrete with incorporating various machine learning algorithms with SHAP analysis. Asian J. Civ. Eng..

[26] Sathvik S., Oyebisi S., Kumar R., Shakor P., Adejonwo O., Tantri A., Suma V. (2025). Analyzing the influence of manufactured sand and fly ash on concrete strength through experimental and machine learning methods. Sci. Rep..

[27] Ahmadi M., Kioumarsi M. (2023). Predicting the elastic modulus of normal and high strength concretes using hybrid ANN-PSO. Mater. Today Proc..

[28] Shaaban M., Amin M., Selim S., Riad I.M. (2025). Machine learning approaches for forecasting compressive strength of high-strength concrete. Sci. Rep..

[29] Karakuzu K. (2023). Effect of Water Reducing Admixture Anionic Monomer Change on Behavior of Cement Systems Having Different C3A Content. Ph.D. Thesis.

[30] Karakuzu K., Kobya V., Mardani A., Felekoğlu B., Ramyar K. (2024). Role of Anionic Group of Methylallyl Ether-Based PCEs on Behavior of Cementitious Systems with Various C3A Contents. J. Mater. Civ. Eng..

[31] Friedman M. (1940). A Comparison of Alternative Tests of Significance for the Problem of m Rankings. Ann. Math. Stat..

[32] Jēkabsons G. (2016). ARESLab.

[33] Cortes C., Vapnik V. (1995). Support-vector networks. Mach. Learn..

[34] Smola A.J., Schölkopf B. (2004). A tutorial on support vector regression. Stat. Comput..

[35] Breiman L., Friedman J., Stone C.J., Olshen R.A. (1984). Classification and Regression Trees.

[36] Hastie T., Tibshirani R., Friedman J. (2009). The Elements of Statistical Learning: Data Mining, Inference, and Prediction.

[37] Breiman L. (2001). Random forests. Mach. Learn..

[38] Liaw A., Wiener M. (2002). Classification and regression by randomForest. R News.

[39] Altman N.S. (1992). An introduction to kernel and nearest-neighbor nonparametric regression. Am. Stat..

[40] Quinlan J.R. (1992). Learning with continuous classes. 5th Australian Joint Conference on Artificial Intelligence.

[41] Wang Y., Witten I.H. (1997). Induction of model trees for predicting continuous classes. Proceedings of the 9th European Conference on Machine Learning.

[42] Kaya Y., Öztürk H.T., Kobya V., Mardani N., Mardani A. (2025). Experimentally and Modeling Assessment of Parameters Affecting Grinding Aid-Containing Cement–PCE Compatibility: CRA, MARS and AOMA-ANN Methods. Polymers.

[43] Shahrouzi M., Kaveh A. (2022). An efficient derivative-free optimization algorithm inspired by avian life-saving manoeuvres. J. Comput. Sci..

[44] Mohamed A.W., Hadi A.A., Mohamed A.K. (2020). Gaining-sharing knowledge based algorithm for solving optimization problems: A novel nature-inspired algorithm. Int. J. Mach. Learn. Cybern..

[45] Abdel-Basset M., Mohamed R., Azeem S.A.A., Jameel M., Abouhawwash M. (2023). Kepler optimization algorithm: A new metaheuristic algorithm inspired by Kepler’s laws of planetary motion. Knowl.-Based Syst..

[46] Faramarzi A., Heidarinejad M., Mirjalili S., Gandomi A.H. (2020). Marine Predators Algorithm: A nature-inspired metaheuristic. Expert Syst. Appl..

[47] Su H., Zhao D., Heidari A.A., Liu L., Zhang X., Mafarja M., Chen H. (2023). RIME: A physics-based optimization. Neurocomputing.

[48] Li H., Yao Y., Wang Z., Cui S., Wang Y. (2020). Influence of monomer ratios on molecular weight properties and dispersing effectiveness in polycarboxylate superplasticizers. Materials.

[49] Hashim F.A., Hussien A.G. (2022). Snake Optimizer: A novel meta-heuristic optimization algorithm. Knowl.-Based Syst..

[50] Cheng M.Y., Prayogo D. (2014). Symbiotic Organisms Search: A new metaheuristic optimization algorithm. Comput. Struct..

[51] Chen X., Xu B. (2018). Teaching-learning-based artificial bee colony. Lecture Notes in Computer Science (Including Subseries Lecture Notes in Artificial Intelligence and Lecture Notes in Bioinformatics): Vol. 10941 LNCS.

[52] Sapna S. (2012). Backpropagation Learning Algorithm Based on Levenberg Marquardt Algorithm. Comput. Sci. Inf. Technol. (CSIT).

[53] Roussel N., Lemaître A., Flatt R.J., Coussot P. (2010). Steady state flow of cement suspensions: A micromechanical state of the art. Cem. Concr. Res..

[54] Kobya V., Karakuzu K., Mardani A., Felekoğlu B., Ramyar K. (2024). Effect of polycarboxylate-based water-reducing admixture chains length on portland cement-admixture compatibility. J. Sustain. Cem.-Based Mater..

[55] Karakuzu K., Kobya V., Mardani A., Felekoğlu B., Ramyar K. (2025). Effect of PCE anionic charge density on fly ash cementitious system-PCE compatibility. J. Adhes. Sci. Technol..

[56] Qian Y. (2021). Effect of polycarboxylate ether (PCE) superplasticizer on thixotropic structural build-up of fresh cement pastes over time. Constr. Build. Mater..

[57] Aïtcin P.C. (2004). High Performance Concrete.

[58] He Y., Zhang X., Hooton R.D. (2017). Effects of organosilane-modified polycarboxylate superplasticizer on the fluidity and hydration properties of cement paste. Constr. Build. Mater..

[59] Kobya V., Karakuzu K., Mardani A., Felekoğlu B., Ramyar K. (2023). Effect of chain characteristics of polycarboxylate-based water-reducing admixtures on behavior of cementitious systems: A Review. J. Mater. Civ. Eng..

[60] Plank J., Brandl A., Lummer N.R. (2007). Effect of different anchor groups on adsorption behavior and effectiveness of poly (N, N-dimethylacrylamide-co-Ca 2-acrylamido-2-methylpropanesulfonate) as cement fluid loss additive in presence of acetone–formaldehyde–sulfite dispersant. J. Appl. Polym. Sci..

[61] Stecher J., Plank J. (2019). Novel concrete superplasticizers based on phosphate esters. Cem. Concr. Res..

[62] He Y., Shu X., Wang X., Yang Y., Liu J., Ran Q. (2019). Effects of polycarboxylates with different adsorption groups on the rheological properties of cement paste. J. Dispers. Sci. Technol..

[63] Sun W., Pan L., Li J., Xu N., Guo Z. (2023). Enhancing the application of mechanochemistry in the synthesis of high-concentration polycarboxylate superplasticizer: Is aqueous copolymerization needed?. J. Dispers. Sci. Technol..

[64] Shu X., Ran Q., Liu J., Zhao H., Zhang Q., Wang X., Liu J. (2016). Tailoring the solution conformation of polycarboxylate superplasticizer toward the improvement of dispersing performance in cement paste. Constr. Build. Mater..

[65] Shu X., Wang Y., Yang Y., Wang X., Zhang Q., Zhao H., Ran Q., Liu J. (2019). Rheological properties of cement pastes with polycarboxylate superplasticizers of varied backbone stiffness. J. Mater. Civ. Eng..

[66] Hirata T., Branicio P., Ye J., Zheng J., Tomike Y., Lange A., Plank J., Sullivan M. (2017). Atomistic dynamics simulation to solve conformation of model PCE superplasticisers in water and cement pore solution. Adv. Cem. Res..

[67] Wolpert D.H., Macready W.G. (1997). No free lunch theorems for optimization. IEEE Trans. Evol. Comput..

