# Supplementary material for: An Experimental and Modeling Study on the Interaction of Cements with Varying C3A Ratios and Different Water-Reducing Admixtures Using the op-ANN and Various Machine Learning Methods
Source: Polymers (Basel). 2026 Mar 7;18(5):656. doi: 10.3390/polym18050656 (PMC12986647; doi:10.3390/polym18050656)
Supplement: Supplementary file 1 [file polymers-18-00656-s001.zip › polymers-4141904-supplementary.pdf]

	Effective parameter																RESULTS	
	Cement Properties							WRA Properties										
													Anionic group content (%)					
	**Fineness**	**C3A**	**C3S**	**C2S**	**C4AF**	**Equivalent Alkaline**	**WRA dosages (% by weight of cement)**	**Mw**	**Mn**	**Main chain length (k)**	**Side chain length (g/mol)**	**Anionic/ Nonionic ratio**	**Carboxylate**	**Phosphate**	**Sulfonate**	**pH**	**DYS (Pa)**	**Viscosity (Pa*s)**
**C2-0.32-WRA1**	3786	2,13	58,98	9,8	16,83	0,685	0	56000	26200	21	2400	3	100	0	0	3,97	66,8	12,9
	3786	2,13	58,98	9,8	16,83	0,685	0,1	56000	26200	21	2400	3	100	0	0	3,97	23,9	8,6
	3786	2,13	58,98	9,18	16,83	0,685	0,15	56000	26200	21	2400	3	100	0	0	3,97	11,6	3,3
**C2-0.35-WRA1**	3786	2,13	58,98	9,8	16,83	0,685	0	56000	26200	21	2400	3	100	0	0	3,97	38,7	10,6
	3786	2,13	58,98	9,8	16,83	0,685	0,1	56000	26200	21	2400	3	100	0	0	3,97	19,3	8,4
	3786	2,13	58,98	9,18	16,83	0,685	0,15	56000	26200	21	2400	3	100	0	0	3,97	2,9	1
**C2-0.32-WRA2**	3786	2,13	58,98	9,8	16,83	0,685	0	27000	11740	10	2400	3	100	0	0	2,87	66,8	12,9
	3786	2,13	58,98	9,8	16,83	0,685	0,1	27000	11740	10	2400	3	100	0	0	2,87	46,7	13,4
	3786	2,13	58,98	9,18	16,83	0,685	0,15	27000	11740	10	2400	3	100	0	0	2,87	28,1	4,5
**C2-0.35-WRA2**	3786	2,13	58,98	9,8	16,83	0,685	0	27000	11740	10	2400	3	100	0	0	2,87	38,7	10,6
	3786	2,13	58,98	9,8	16,83	0,685	0,1	27000	11740	10	2400	3	100	0	0	2,87	38,2	12,7
	3786	2,13	58,98	9,18	16,83	0,685	0,15	27000	11740	10	2400	3	100	0	0	2,87	9,4	1,6
**C2-0.32-WRA3**	3786	2,13	58,98	9,8	16,83	0,685	0	78000	37140	31	2400	3	100	0	0	3,26	66,8	12,9
	3786	2,13	58,98	9,8	16,83	0,685	0,1	78000	37140	31	2400	3	100	0	0	3,26	47,3	16,2
	3786	2,13	58,98	9,18	16,83	0,685	0,15	78000	37140	31	2400	3	100	0	0	3,26	27,8	4,6
**C2-0.35-WRA3**	3786	2,13	58,98	9,8	16,83	0,685	0	78000	37140	31	2400	3	100	0	0	3,26	38,7	10,6
	3786	2,13	58,98	9,8	16,83	0,685	0,1	78000	37140	31	2400	3	100	0	0	3,26	47,7	13,1
	3786	2,13	58,98	9,18	16,83	0,685	0,15	78000	37140	31	2400	3	100	0	0	3,26	11,3	2,1
**C2-0.32-WRA4**	3786	2,13	58,98	9,8	16,83	0,685	0	26000	11300	21	1000	3	100	0	0	3,16	66,8	12,9
	3786	2,13	58,98	9,8	16,83	0,685	0,1	26000	11300	21	1000	3	100	0	0	3,16	27,3	11,9
	3786	2,13	58,98	9,18	16,83	0,685	0,15	26000	11300	21	1000	3	100	0	0	3,16	11,8	3,6
**C2-0.35-WRA4**	3786	2,13	58,98	9,8	16,83	0,685	0	26000	11300	21	1000	3	100	0	0	3,16	38,7	10,6
	3786	2,13	58,98	9,8	16,83	0,685	0,1	26000	11300	21	1000	3	100	0	0	3,16	21,5	9,9

	3786	2,13	58,98	9,18	16,83	0,685	0,15	26000	11300	21	1000	3	100	0	0	3,16	5,0	1,5
**C2-0.32-WRA5**	3786	2,13	58,98	9,8	16,83	0,685	0	69000	34500	21	3000	3	100	0	0	3,44	66,8	12,9
	3786	2,13	58,98	9,8	16,83	0,685	0,1	69000	34500	21	3000	3	100	0	0	3,44	34,4	9,8
	3786	2,13	58,98	9,18	16,83	0,685	0,15	69000	34500	21	3000	3	100	0	0	3,44	8,0	2,3
**C2-0.35-WRA5**	3786	2,13	58,98	9,8	16,83	0,685	0	69000	34500	21	3000	3	100	0	0	3,44	38,7	10,6
	3786	2,13	58,98	9,8	16,83	0,685	0,1	69000	34500	21	3000	3	100	0	0	3,44	17,8	9,3
	3786	2,13	58,98	9,18	16,83	0,685	0,15	69000	34500	21	3000	3	100	0	0	3,44	2,3	1
**C2-0.32-WRA6**	3786	2,13	58,98	9,8	16,83	0,685	0	56000	24340	40	1000	3	100	0	0	3,27	66,8	12,9
	3786	2,13	58,98	9,8	16,83	0,685	0,1	56000	24340	40	1000	3	100	0	0	3,27	19,2	9,8
	3786	2,13	58,98	9,18	16,83	0,685	0,15	56000	24340	40	1000	3	100	0	0	3,27	7,2	2,3
**C2-0.35-WRA6**	3786	2,13	58,98	9,8	16,83	0,685	0	56000	24340	40	1000	3	100	0	0	3,27	38,7	10,6
	3786	2,13	58,98	9,8	16,83	0,685	0,1	56000	24340	40	1000	3	100	0	0	3,27	15,3	7,3
	3786	2,13	58,98	9,18	16,83	0,685	0,15	56000	24340	40	1000	3	100	0	0	3,27	2,8	1,1
**C2-0.32-WRA7**	3786	2,13	58,98	9,8	16,83	0,685	0	57000	27140	17	3000	3	100	0	0	3,27	66,8	12,9
	3786	2,13	58,98	9,8	16,83	0,685	0,1	57000	27140	17	3000	3	100	0	0	3,27	25,6	9,3
	3786	2,13	58,98	9,18	16,83	0,685	0,15	57000	27140	17	3000	3	100	0	0	3,27	12,1	3,7
**C2-0.35-WRA7**	3786	2,13	58,98	9,8	16,83	0,685	0	57000	27140	17	3000	3	100	0	0	3,27	38,7	10,6
	3786	2,13	58,98	9,8	16,83	0,685	0,1	57000	27140	17	3000	3	100	0	0	3,27	18,6	6,8
	3786	2,13	58,98	9,18	16,83	0,685	0,15	57000	27140	17	3000	3	100	0	0	3,27	3,9	1,1
**C2-0.32-WRA8**	3786	2,13	58,98	9,8	16,83	0,685	0	58000	26300	21	2400	3	99	1	0	2,91	66,8	12,9
	3786	2,13	58,98	9,8	16,83	0,685	0,1	58000	26300	21	2400	3	99	1	0	2,91	37,5	9,9
	3786	2,13	58,98	9,18	16,83	0,685	0,15	58000	26300	21	2400	3	99	1	0	2,91	13,0	3,2
**C2-0.35-WRA8**	3786	2,13	58,98	9,8	16,83	0,685	0	58000	26300	21	2400	3	99	1	0	2,91	38,7	10,6
	3786	2,13	58,98	9,8	16,83	0,685	0,1	58000	26300	21	2400	3	99	1	0	2,91	54,8	7
	3786	2,13	58,98	9,18	16,83	0,685	0,15	58000	26300	21	2400	3	99	1	0	2,91	6,9	1,1
**C2-0.32-WRA9**	3786	2,13	58,98	9,8	16,83	0,685	0	65000	27000	21	2400	3	97	3	0	2,93	66,8	12,9
	3786	2,13	58,98	9,8	16,83	0,685	0,1	65000	27000	21	2400	3	97	3	0	2,93	43,4	10,6
	3786	2,13	58,98	9,18	16,83	0,685	0,15	65000	27000	21	2400	3	97	3	0	2,93	12,6	3,6
**C2-0.35-WRA9**	3786	2,13	58,98	9,8	16,83	0,685	0	65000	27000	21	2400	3	97	3	0	2,93	38,7	10,6
	3786	2,13	58,98	9,8	16,83	0,685	0,1	65000	27000	21	2400	3	97	3	0	2,93	15,2	7
	3786	2,13	58,98	9,18	16,83	0,685	0,15	65000	27000	21	2400	3	97	3	0	2,93	6,8	1,1
**C2-0.32-WRA10**	3786	2,13	58,98	9,8	16,83	0,685	0	61000	26500	21	2400	3	95	5	0	2,93	66,8	12,9
	3786	2,13	58,98	9,8	16,83	0,685	0,1	61000	26500	21	2400	3	95	5	0	2,93	39,3	10,2

	3786	2,13	58,98	9,18	16,83	0,685	0,15	61000	26500	21	2400	3	95	5	0	2,93	12,4	3,7
	3786	2,13	58,98	9,8	16,83	0,685	0	61000	26500	21	2400	3	95	5	0	2,93	38,7	10,6
**C2-0.35-WRA10**	3786	2,13	58,98	9,8	16,83	0,685	0,1	61000	26500	21	2400	3	95	5	0	2,93	23,2	7,1
	3786	2,13	58,98	9,18	16,83	0,685	0,15	61000	26500	21	2400	3	95	5	0	2,93	2,9	1,1
	3786	2,13	58,98	9,8	16,83	0,685	0	56100	26600	21	2400	3	93	7	0	2,65	66,8	12,9
**C2-0.32-WRA11**	3786	2,13	58,98	9,8	16,83	0,685	0,1	56100	26600	21	2400	3	93	7	0	2,65	38,7	10,4
	3786	2,13	58,98	9,18	16,83	0,685	0,15	56100	26600	21	2400	3	93	7	0	2,65	9,3	2,8
	3786	2,13	58,98	9,8	16,83	0,685	0	56100	26600	21	2400	3	93	7	0	2,65	38,7	10,6
**C2-0.35-WRA11**	3786	2,13	58,98	9,8	16,83	0,685	0,1	56100	26600	21	2400	3	93	7	0	2,65	71,0	6,4
	3786	2,13	58,98	9,18	16,83	0,685	0,15	56100	26600	21	2400	3	93	7	0	2,65	7,7	1
	3786	2,13	58,98	9,8	16,83	0,685	0	53000	23000	21	2400	3	91	9	0	2,54	66,8	12,9
**C2-0.32-WRA12**	3786	2,13	58,98	9,8	16,83	0,685	0,1	53000	23000	21	2400	3	91	9	0	2,54	37,1	9,9
	3786	2,13	58,98	9,18	16,83	0,685	0,15	53000	23000	21	2400	3	91	9	0	2,54	14,1	2,6
	3786	2,13	58,98	9,8	16,83	0,685	0	53000	23000	21	2400	3	91	9	0	2,54	38,7	10,6
**C2-0.35-WRA12**	3786	2,13	58,98	9,8	16,83	0,685	0,1	53000	23000	21	2400	3	91	9	0	2,54	25,5	6,6
	3786	2,13	58,98	9,18	16,83	0,685	0,15	53000	23000	21	2400	3	91	9	0	2,54	12,3	1,1
	3786	2,13	58,98	9,8	16,83	0,685	0	52000	22600	21	2400	3	80	20	0	2,61	66,8	12,9
**C2-0.32-WRA13**	3786	2,13	58,98	9,8	16,83	0,685	0,1	52000	22600	21	2400	3	80	20	0	2,61	42,7	11,2
	3786	2,13	58,98	9,18	16,83	0,685	0,15	52000	22600	21	2400	3	80	20	0	2,61	21,5	4,4
	3786	2,13	58,98	9,8	16,83	0,685	0	52000	22600	21	2400	3	80	20	0	2,61	38,7	10,6
**C2-0.35-WRA13**	3786	2,13	58,98	9,8	16,83	0,685	0,1	52000	22600	21	2400	3	80	20	0	2,61	68,1	7,2
	3786	2,13	58,98	9,18	16,83	0,685	0,15	52000	22600	21	2400	3	80	20	0	2,61	7,7	1,2
	3786	2,13	58,98	9,8	16,83	0,685	0	62100	29500	21	2400	3	99	0	1	1,08	66,8	12,9
**C2-0.32-WRA14**	3786	2,13	58,98	9,8	16,83	0,685	0,1	62100	29500	21	2400	3	99	0	1	1,08	45,4	12,1
	3786	2,13	58,98	9,18	16,83	0,685	0,15	62100	29500	21	2400	3	99	0	1	1,08	10,2	3,1
	3786	2,13	58,98	9,8	16,83	0,685	0	62100	29500	21	2400	3	99	0	1	1,08	38,7	10,6
**C2-0.35-WRA14**	3786	2,13	58,98	9,8	16,83	0,685	0,1	62100	29500	21	2400	3	99	0	1	1,08	63,7	8,3
	3786	2,13	58,98	9,18	16,83	0,685	0,15	62100	29500	21	2400	3	99	0	1	1,08	8,6	1
	3786	2,13	58,98	9,8	16,83	0,685	0	63000	28600	21	2400	3	97	0	3	1,08	66,8	12,9
**C2-0.32-WRA15**	3786	2,13	58,98	9,8	16,83	0,685	0,1	63000	28600	21	2400	3	97	0	3	1,08	38,6	10,3
	3786	2,13	58,98	9,18	16,83	0,685	0,15	63000	28600	21	2400	3	97	0	3	1,08	14,7	3,4
**C2-0.35-WRA15**	3786	2,13	58,98	9,8	16,83	0,685	0	63000	28600	21	2400	3	97	0	3	1,08	38,7	10,6
	3786	2,13	58,98	9,8	16,83	0,685	0,1	63000	28600	21	2400	3	97	0	3	1,08	83,2	9

	3786	2,13	58,98	9,18	16,83	0,685	0,15	63000	28600	21	2400	3	97	0	3	1,08	8,0	1,1
**C2-0.32-WRA16**	3786	2,13	58,98	9,8	16,83	0,685	0	58000	29000	21	2400	3	95	0	5	1,08	66,8	6,8
	3786	2,13	58,98	9,8	16,83	0,685	0,1	58000	29000	21	2400	3	95	0	5	1,08	34,3	9
	3786	2,13	58,98	9,18	16,83	0,685	0,15	58000	29000	21	2400	3	95	0	5	1,08	13,8	3,4
**C2-0.35-WRA16**	3786	2,13	58,98	9,8	16,83	0,685	0	58000	29000	21	2400	3	95	0	5	1,08	38,7	10,6
	3786	2,13	58,98	9,8	16,83	0,685	0,1	58000	29000	21	2400	3	95	0	5	1,08	22,1	8,8
	3786	2,13	58,98	9,18	16,83	0,685	0,15	58000	29000	21	2400	3	95	0	5	1,08	10,5	1,2
**C2-0.32-WRA17**	3786	2,13	58,98	9,8	16,83	0,685	0	63000	28600	21	2400	3	93	0	7	1,08	66,8	12,9
	3786	2,13	58,98	9,8	16,83	0,685	0,1	63000	28600	21	2400	3	93	0	7	1,08	32,9	8
	3786	2,13	58,98	9,18	16,83	0,685	0,15	63000	28600	21	2400	3	93	0	7	1,08	9,8	2,4
**C2-0.35-WRA17**	3786	2,13	58,98	9,8	16,83	0,685	0	63000	28600	21	2400	3	93	0	7	1,08	38,7	10,6
	3786	2,13	58,98	9,8	16,83	0,685	0,1	63000	28600	21	2400	3	93	0	7	1,08	56,6	8
	3786	2,13	58,98	9,18	16,83	0,685	0,15	63000	28600	21	2400	3	93	0	7	1,08	4,9	0,8
**C2-0.32-WRA18**	3786	2,13	58,98	9,8	16,83	0,685	0	60000	25000	21	2400	3	91	0	9	1,08	66,8	12,9
	3786	2,13	58,98	9,8	16,83	0,685	0,1	60000	25000	21	2400	3	91	0	9	1,08	32,5	10,2
	3786	2,13	58,98	9,18	16,83	0,685	0,15	60000	25000	21	2400	3	91	0	9	1,08	8,9	3,1
**C2-0.35-WRA18**	3786	2,13	58,98	9,8	16,83	0,685	0	60000	25000	21	2400	3	91	0	9	1,08	38,7	10,6
	3786	2,13	58,98	9,8	16,83	0,685	0,1	60000	25000	21	2400	3	91	0	9	1,08	75,0	8,4
	3786	2,13	58,98	9,18	16,83	0,685	0,15	60000	25000	21	2400	3	91	0	9	1,08	11,1	1,3
**C2-0.32-WRA19**	3786	2,13	58,98	9,8	16,83	0,685	0	61000	29000	21	2400	3	80	0	20	1,08	66,8	12,9
	3786	2,13	58,98	9,8	16,83	0,685	0,1	61000	29000	21	2400	3	80	0	20	1,08	29,0	9,7
	3786	2,13	58,98	9,18	16,83	0,685	0,15	61000	29000	21	2400	3	80	0	20	1,08	17,6	10,6
**C2-0.35-WRA19**	3786	2,13	58,98	9,8	16,83	0,685	0	61000	29000	21	2400	3	80	0	20	1,08	38,7	3,8
	3786	2,13	58,98	9,8	16,83	0,685	0,1	61000	29000	21	2400	3	80	0	20	1,08	67,0	8,7
	3786	2,13	58,98	9,18	16,83	0,685	0,15	61000	29000	21	2400	3	80	0	20	1,08	8,8	1,2
**C2-0.32-WRA20**	3786	2,13	58,98	9,8	16,83	0,685	0	63000	30000	21	2400	3	100	0	0	3,54	66,8	12,9
	3786	2,13	58,98	9,8	16,83	0,685	0,1	63000	30000	21	2400	3	100	0	0	3,54	26,8	6,8
	3786	2,13	58,98	9,18	16,83	0,685	0,15	63000	30000	21	2400	3	100	0	0	3,54	10,1	2,8
**C2-0.35-WRA20**	3786	2,13	58,98	9,8	16,83	0,685	0	63000	30000	21	2400	3	100	0	0	3,54	38,7	10,6
	3786	2,13	58,98	9,8	16,83	0,685	0,1	63000	30000	21	2400	3	100	0	0	3,54	16,3	5,3
	3786	2,13	58,98	9,18	16,83	0,685	0,15	63000	30000	21	2400	3	100	0	0	3,54	3,7	1,1
**C2-0.32-WRA21**	3786	2,13	58,98	9,8	16,83	0,685	0	56000	26500	21	2400	3	100	0	0	2,94	66,8	12,9
	3786	2,13	58,98	9,8	16,83	0,685	0,1	56000	26500	21	2400	3	100	0	0	2,94	15,9	5,4

	3786	2,13	58,98	9,18	16,83	0,685	0,15	56000	26500	21	2400	3	100	0	0	2,94	5,0	0,9
	3786	2,13	58,98	9,8	16,83	0,685	0	56000	26500	21	2400	3	100	0	0	2,94	38,7	10,6
**C2-0.35-WRA21**	3786	2,13	58,98	9,8	16,83	0,685	0,1	56000	26500	21	2400	3	100	0	0	2,94	4,2	4,7
	3786	2,13	58,98	9,18	16,83	0,685	0,15	56000	26500	21	2400	3	100	0	0	2,94	2,0	0,7
	3786	2,13	58,98	9,8	16,83	0,685	0	60000	26000	21	2400	3	91	9	0	2,97	66,8	12,9
**C2-0.32-WRA22**	3786	2,13	58,98	9,8	16,83	0,685	0,1	60000	26000	21	2400	3	91	9	0	2,97	27,6	8,3
	3786	2,13	58,98	9,18	16,83	0,685	0,15	60000	26000	21	2400	3	91	9	0	2,97	34,6	2,5
	3786	2,13	58,98	9,8	16,83	0,685	0	60000	26000	21	2400	3	91	9	0	2,97	38,7	10,6
**C2-0.35-WRA22**	3786	2,13	58,98	9,8	16,83	0,685	0,1	60000	26000	21	2400	3	91	9	0	2,97	18,2	5,3
	3786	2,13	58,98	9,18	16,83	0,685	0,15	60000	26000	21	2400	3	91	9	0	2,97	11,9	1,6
	3786	2,13	58,98	9,8	16,83	0,685	0	54000	22500	21	2400	3	91	9	0	3,21	66,8	12,9
**C2-0.32-WRA23**	3786	2,13	58,98	9,8	16,83	0,685	0,1	54000	22500	21	2400	3	91	9	0	3,21	57,2	7,3
	3786	2,13	58,98	9,18	16,83	0,685	0,15	54000	22500	21	2400	3	91	9	0	3,21	9,4	2,4
	3786	2,13	58,98	9,8	16,83	0,685	0	54000	22500	21	2400	3	91	9	0	3,21	38,7	10,6
**C2-0.35-WRA23**	3786	2,13	58,98	9,8	16,83	0,685	0,1	54000	22500	21	2400	3	91	9	0	3,21	15,5	5
	3786	2,13	58,98	9,18	16,83	0,685	0,15	54000	22500	21	2400	3	91	9	0	3,21	4,8	1,2
	3786	2,13	58,98	9,8	16,83	0,685	0	62000	29000	21	2400	3	91	0	9	3,24	66,8	12,9
**C2-0.32-WRA24**	3786	2,13	58,98	9,8	16,83	0,685	0,1	62000	29000	21	2400	3	91	0	9	3,24	38,3	8
	3786	2,13	58,98	9,18	16,83	0,685	0,15	62000	29000	21	2400	3	91	0	9	3,24	15,2	3,4
	3786	2,13	58,98	9,8	16,83	0,685	0	62000	29000	21	2400	3	91	0	9	3,24	38,7	10,6
**C2-0.35-WRA24**	3786	2,13	58,98	9,8	16,83	0,685	0,1	62000	29000	21	2400	3	91	0	9	3,24	16,4	6,4
	3786	2,13	58,98	9,18	16,83	0,685	0,15	62000	29000	21	2400	3	91	0	9	3,24	5,6	1,5
	3786	2,13	58,98	9,8	16,83	0,685	0	56000	28000	21	2400	3	91	0	9	3,58	66,8	12,9
**C2-0.32-WRA25**	3786	2,13	58,98	9,8	16,83	0,685	0,1	56000	28000	21	2400	3	91	0	9	3,58	27,2	7,5
	3786	2,13	58,98	9,18	16,83	0,685	0,15	56000	28000	21	2400	3	91	0	9	3,58	4,1	1,8
	3786	2,13	58,98	9,8	16,83	0,685	0	56000	28000	21	2400	3	91	0	9	3,58	38,7	10,6
**C2-0.35-WRA25**	3786	2,13	58,98	9,8	16,83	0,685	0,1	56000	28000	21	2400	3	91	0	9	3,58	11,2	5,1
	3786	2,13	58,98	9,18	16,83	0,685	0,15	56000	28000	21	2400	3	91	0	9	3,58	2,1	0,7
	3754	3,6	47,6	20,22	16,04	0,675	0	56000	26200	21	2400	3	100	0	0	3,97	94,3	10,1
**C3-0.32-WRA1**	3754	3,6	47,6	20,22	16,04	0,675	0,1	56000	26200	21	2400	3	100	0	0	3,97	32,4	6,8
	3754	3,6	47,6	20,22	16,04	0,675	0,15	56000	26200	21	2400	3	100	0	0	3,97	9,3	2,9
**C3-0.35-WRA1**	3754	3,6	47,6	20,22	16,04	0,675	0	56000	26200	21	2400	3	100	0	0	3,97	40,1	4,3
	3754	3,6	47,6	20,22	16,04	0,675	0,1	56000	26200	21	2400	3	100	0	0	3,97	11,6	2,9

	3754	3,6	47,6	20,22	16,04	0,675	0,15	56000	26200	21	2400	3	100	0	0	3,97	3,9	1,1
**C3-0.32-WRA2**	3754	3,6	47,6	20,22	16,04	0,675	0	27000	11740	10	2400	3	100	0	0	2,87	94,3	10,1
	3754	3,6	47,6	20,22	16,04	0,675	0,1	27000	11740	10	2400	3	100	0	0	2,87	48,7	7,6
	3754	3,6	47,6	20,22	16,04	0,675	0,15	27000	11740	10	2400	3	100	0	0	2,87	43,5	6,1
**C3-0.35-WRA2**	3754	3,6	47,6	20,22	16,04	0,675	0	27000	11740	10	2400	3	100	0	0	2,87	40,1	4,3
	3754	3,6	47,6	20,22	16,04	0,675	0,1	27000	11740	10	2400	3	100	0	0	2,87	20,3	4,6
	3754	3,6	47,6	20,22	16,04	0,675	0,15	27000	11740	10	2400	3	100	0	0	2,87	12,5	2,5
**C3-0.32-WRA3**	3754	3,6	47,6	20,22	16,04	0,675	0	78000	37140	31	2400	3	100	0	0	3,26	94,3	10,1
	3754	3,6	47,6	20,22	16,04	0,675	0,1	78000	37140	31	2400	3	100	0	0	3,26	41,4	7,3
	3754	3,6	47,6	20,22	16,04	0,675	0,15	78000	37140	31	2400	3	100	0	0	3,26	37,9	5,5
**C3-0.35-WRA3**	3754	3,6	47,6	20,22	16,04	0,675	0	78000	37140	31	2400	3	100	0	0	3,26	40,1	4,3
	3754	3,6	47,6	20,22	16,04	0,675	0,1	78000	37140	31	2400	3	100	0	0	3,26	20,8	4,4
	3754	3,6	47,6	20,22	16,04	0,675	0,15	78000	37140	31	2400	3	100	0	0	3,26	12,9	2,6
**C3-0.32-WRA4**	3754	3,6	47,6	20,22	16,04	0,675	0	26000	11300	21	1000	3	100	0	0	3,16	94,3	10,1
	3754	3,6	47,6	20,22	16,04	0,675	0,1	26000	11300	21	1000	3	100	0	0	3,16	28,4	7,2
	3754	3,6	47,6	20,22	16,04	0,675	0,15	26000	11300	21	1000	3	100	0	0	3,16	13,1	3,8
**C3-0.35-WRA4**	3754	3,6	47,6	20,22	16,04	0,675	0	26000	11300	21	1000	3	100	0	0	3,16	40,1	4,3
	3754	3,6	47,6	20,22	16,04	0,675	0,1	26000	11300	21	1000	3	100	0	0	3,16	13,6	3,9
	3754	3,6	47,6	20,22	16,04	0,675	0,15	26000	11300	21	1000	3	100	0	0	3,16	12,5	1,5
**C3-0.32-WRA5**	3754	3,6	47,6	20,22	16,04	0,675	0	69000	34500	21	3000	3	100	0	0	3,44	94,3	10,1
	3754	3,6	47,6	20,22	16,04	0,675	0,1	69000	34500	21	3000	3	100	0	0	3,44	27,2	6,9
	3754	3,6	47,6	20,22	16,04	0,675	0,15	69000	34500	21	3000	3	100	0	0	3,44	12,6	3,5
**C3-0.35-WRA5**	3754	3,6	47,6	20,22	16,04	0,675	0	69000	34500	21	3000	3	100	0	0	3,44	40,1	4,3
	3754	3,6	47,6	20,22	16,04	0,675	0,1	69000	34500	21	3000	3	100	0	0	3,44	9,7	3,5
	3754	3,6	47,6	20,22	16,04	0,675	0,15	69000	34500	21	3000	3	100	0	0	3,44	2,4	0,7
**C3-0.32-WRA6**	3754	3,6	47,6	20,22	16,04	0,675	0	56000	24340	40	1000	3	100	0	0	3,27	94,3	10,1
	3754	3,6	47,6	20,22	16,04	0,675	0,1	56000	24340	40	1000	3	100	0	0	3,27	35,7	7,2
	3754	3,6	47,6	20,22	16,04	0,675	0,15	56000	24340	40	1000	3	100	0	0	3,27	23,5	4,2
**C3-0.35-WRA6**	3754	3,6	47,6	20,22	16,04	0,675	0	56000	24340	40	1000	3	100	0	0	3,27	40,1	4,3
	3754	3,6	47,6	20,22	16,04	0,675	0,1	56000	24340	40	1000	3	100	0	0	3,27	12,6	3,8
	3754	3,6	47,6	20,22	16,04	0,675	0,15	56000	24340	40	1000	3	100	0	0	3,27	4,8	1,2
**C3-0.32-WRA7**	3754	3,6	47,6	20,22	16,04	0,675	0	57000	27140	17	3000	3	100	0	0	3,27	94,3	10,1
	3754	3,6	47,6	20,22	16,04	0,675	0,1	57000	27140	17	3000	3	100	0	0	3,27	26,8	6,9

	3754	3,6	47,6	20,22	16,04	0,675	0,15	57000	27140	17	3000	3	100	0	0	3,27	12,3	3,5
**C3-0.35-WRA7**	3754	3,6	47,6	20,22	16,04	0,675	0	57000	27140	17	3000	3	100	0	0	3,27	40,1	4,3
	3754	3,6	47,6	20,22	16,04	0,675	0,1	57000	27140	17	3000	3	100	0	0	3,27	12,8	3,9
	3754	3,6	47,6	20,22	16,04	0,675	0,15	57000	27140	17	3000	3	100	0	0	3,27	3,9	1,2
**C3-0.32-WRA8**	3754	3,6	47,6	20,22	16,04	0,675	0	58000	26300	21	2400	3	99	1	0	2,91	94,3	10,1
	3754	3,6	47,6	20,22	16,04	0,675	0,1	58000	26300	21	2400	3	99	1	0	2,91	39,2	7,6
	3754	3,6	47,6	20,22	16,04	0,675	0,15	58000	26300	21	2400	3	99	1	0	2,91	16,1	4
**C3-0.35-WRA8**	3754	3,6	47,6	20,22	16,04	0,675	0	58000	26300	21	2400	3	99	1	0	2,91	40,1	4,3
	3754	3,6	47,6	20,22	16,04	0,675	0,1	58000	26300	21	2400	3	99	1	0	2,91	19,3	3,6
	3754	3,6	47,6	20,22	16,04	0,675	0,15	58000	26300	21	2400	3	99	1	0	2,91	11,2	1,5
**C3-0.32-WRA9**	3754	3,6	47,6	20,22	16,04	0,675	0	65000	27000	21	2400	3	97	3	0	2,93	94,3	10,1
	3754	3,6	47,6	20,22	16,04	0,675	0,1	65000	27000	21	2400	3	97	3	0	2,93	33,9	7
	3754	3,6	47,6	20,22	16,04	0,675	0,15	65000	27000	21	2400	3	97	3	0	2,93	15,3	3,8
**C3-0.35-WRA9**	3754	3,6	47,6	20,22	16,04	0,675	0	65000	27000	21	2400	3	97	3	0	2,93	40,1	4,3
	3754	3,6	47,6	20,22	16,04	0,675	0,1	65000	27000	21	2400	3	97	3	0	2,93	19,3	3,6
	3754	3,6	47,6	20,22	16,04	0,675	0,15	65000	27000	21	2400	3	97	3	0	2,93	10,5	1,4
**C3-0.32-WRA10**	3754	3,6	47,6	20,22	16,04	0,675	0	61000	26500	21	2400	3	95	5	0	2,93	94,3	10,1
	3754	3,6	47,6	20,22	16,04	0,675	0,1	61000	26500	21	2400	3	95	5	0	2,93	38,8	7,2
	3754	3,6	47,6	20,22	16,04	0,675	0,15	61000	26500	21	2400	3	95	5	0	2,93	16,7	3,7
**C3-0.35-WRA10**	3754	3,6	47,6	20,22	16,04	0,675	0	61000	26500	21	2400	3	95	5	0	2,93	40,1	4,3
	3754	3,6	47,6	20,22	16,04	0,675	0,1	61000	26500	21	2400	3	95	5	0	2,93	14,4	3,5
	3754	3,6	47,6	20,22	16,04	0,675	0,15	61000	26500	21	2400	3	95	5	0	2,93	12,0	1,6
**C3-0.32-WRA11**	3754	3,6	47,6	20,22	16,04	0,675	0	56100	26600	21	2400	3	93	7	0	2,65	94,3	10,1
	3754	3,6	47,6	20,22	16,04	0,675	0,1	56100	26600	21	2400	3	93	7	0	2,65	36,2	6,9
	3754	3,6	47,6	20,22	16,04	0,675	0,15	56100	26600	21	2400	3	93	7	0	2,65	17,8	4,1
**C3-0.35-WRA11**	3754	3,6	47,6	20,22	16,04	0,675	0	56100	26600	21	2400	3	93	7	0	2,65	40,1	4,3
	3754	3,6	47,6	20,22	16,04	0,675	0,1	56100	26600	21	2400	3	93	7	0	2,65	19,4	3,7
	3754	3,6	47,6	20,22	16,04	0,675	0,15	56100	26600	21	2400	3	93	7	0	2,65	13,1	1,4
**C3-0.32-WRA12**	3754	3,6	47,6	20,22	16,04	0,675	0	53000	23000	21	2400	3	91	9	0	2,54	94,3	10,1
	3754	3,6	47,6	20,22	16,04	0,675	0,1	53000	23000	21	2400	3	91	9	0	2,54	31,3	6,5
	3754	3,6	47,6	20,22	16,04	0,675	0,15	53000	23000	21	2400	3	91	9	0	2,54	22,1	3,6
**C3-0.35-WRA12**	3754	3,6	47,6	20,22	16,04	0,675	0	53000	23000	21	2400	3	91	9	0	2,54	40,1	4,3
	3754	3,6	47,6	20,22	16,04	0,675	0,1	53000	23000	21	2400	3	91	9	0	2,54	14,6	3,4

	3754	3,6	47,6	20,22	16,04	0,675	0,15	53000	23000	21	2400	3	91	9	0	2,54	10,5	1,3
**C3-0.32-WRA13**	3754	3,6	47,6	20,22	16,04	0,675	0	52000	22600	21	2400	3	80	20	0	2,61	94,3	10,1
	3754	3,6	47,6	20,22	16,04	0,675	0,1	52000	22600	21	2400	3	80	20	0	2,61	32,7	6,9
	3754	3,6	47,6	20,22	16,04	0,675	0,15	52000	22600	21	2400	3	80	20	0	2,61	22,4	4,4
**C3-0.35-WRA13**	3754	3,6	47,6	20,22	16,04	0,675	0	52000	22600	21	2400	3	80	20	0	2,61	40,1	4,3
	3754	3,6	47,6	20,22	16,04	0,675	0,1	52000	22600	21	2400	3	80	20	0	2,61	17,4	3,5
	3754	3,6	47,6	20,22	16,04	0,675	0,15	52000	22600	21	2400	3	80	20	0	2,61	16,8	1,8
**C3-0.32-WRA14**	3754	3,6	47,6	20,22	16,04	0,675	0	62100	29500	21	2400	3	99	0	1	1,08	94,3	10,1
	3754	3,6	47,6	20,22	16,04	0,675	0,1	62100	29500	21	2400	3	99	0	1	1,08	39,4	7,2
	3754	3,6	47,6	20,22	16,04	0,675	0,15	62100	29500	21	2400	3	99	0	1	1,08	19,4	4,4
**C3-0.35-WRA14**	3754	3,6	47,6	20,22	16,04	0,675	0	62100	29500	21	2400	3	99	0	1	1,08	40,1	4,3
	3754	3,6	47,6	20,22	16,04	0,675	0,1	62100	29500	21	2400	3	99	0	1	1,08	15,5	3,4
	3754	3,6	47,6	20,22	16,04	0,675	0,15	62100	29500	21	2400	3	99	0	1	1,08	10,3	1,3
**C3-0.32-WRA15**	3754	3,6	47,6	20,22	16,04	0,675	0	63000	28600	21	2400	3	97	0	3	1,08	94,3	10,1
	3754	3,6	47,6	20,22	16,04	0,675	0,1	63000	28600	21	2400	3	97	0	3	1,08	37,3	7
	3754	3,6	47,6	20,22	16,04	0,675	0,15	63000	28600	21	2400	3	97	0	3	1,08	19,2	4,2
**C3-0.35-WRA15**	3754	3,6	47,6	20,22	16,04	0,675	0	63000	28600	21	2400	3	97	0	3	1,08	40,1	4,3
	3754	3,6	47,6	20,22	16,04	0,675	0,1	63000	28600	21	2400	3	97	0	3	1,08	13,2	3,3
	3754	3,6	47,6	20,22	16,04	0,675	0,15	63000	28600	21	2400	3	97	0	3	1,08	10,9	1,3
**C3-0.32-WRA16**	3754	3,6	47,6	20,22	16,04	0,675	0	58000	29000	21	2400	3	95	0	5	1,08	94,3	10,1
	3754	3,6	47,6	20,22	16,04	0,675	0,1	58000	29000	21	2400	3	95	0	5	1,08	37,5	7
	3754	3,6	47,6	20,22	16,04	0,675	0,15	58000	29000	21	2400	3	95	0	5	1,08	18,9	4,1
**C3-0.35-WRA16**	3754	3,6	47,6	20,22	16,04	0,675	0	58000	29000	21	2400	3	95	0	5	1,08	40,1	4,3
	3754	3,6	47,6	20,22	16,04	0,675	0,1	58000	29000	21	2400	3	95	0	5	1,08	16,9	3,7
	3754	3,6	47,6	20,22	16,04	0,675	0,15	58000	29000	21	2400	3	95	0	5	1,08	12,5	1,4
**C3-0.32-WRA17**	3754	3,6	47,6	20,22	16,04	0,675	0	63000	28600	21	2400	3	93	0	7	1,08	94,3	10,1
	3754	3,6	47,6	20,22	16,04	0,675	0,1	63000	28600	21	2400	3	93	0	7	1,08	38,6	6,4
	3754	3,6	47,6	20,22	16,04	0,675	0,15	63000	28600	21	2400	3	93	0	7	1,08	20,6	4,6
**C3-0.35-WRA17**	3754	3,6	47,6	20,22	16,04	0,675	0	63000	28600	21	2400	3	93	0	7	1,08	40,1	4,3
	3754	3,6	47,6	20,22	16,04	0,675	0,1	63000	28600	21	2400	3	93	0	7	1,08	16,7	3,8
	3754	3,6	47,6	20,22	16,04	0,675	0,15	63000	28600	21	2400	3	93	0	7	1,08	11,7	1,3
**C3-0.32-WRA18**	3754	3,6	47,6	20,22	16,04	0,675	0	60000	25000	21	2400	3	91	0	9	1,08	94,3	10,1
	3754	3,6	47,6	20,22	16,04	0,675	0,1	60000	25000	21	2400	3	91	0	9	1,08	39,7	6,9

	3754	3,6	47,6	20,22	16,04	0,675	0,15	60000	25000	21	2400	3	91	0	9	1,08	19,6	4,6
	3754	3,6	47,6	20,22	16,04	0,675	0	60000	25000	21	2400	3	91	0	9	1,08	40,1	4,3
**C3-0.35-WRA18**	3754	3,6	47,6	20,22	16,04	0,675	0,1	60000	25000	21	2400	3	91	0	9	1,08	14,7	3,3
	3754	3,6	47,6	20,22	16,04	0,675	0,15	60000	25000	21	2400	3	91	0	9	1,08	9,6	1,3
	3754	3,6	47,6	20,22	16,04	0,675	0	61000	29000	21	2400	3	80	0	20	1,08	94,3	10,1
**C3-0.32-WRA19**	3754	3,6	47,6	20,22	16,04	0,675	0,1	61000	29000	21	2400	3	80	0	20	1,08	40,2	7
	3754	3,6	47,6	20,22	16,04	0,675	0,15	61000	29000	21	2400	3	80	0	20	1,08	25,0	5
	3754	3,6	47,6	20,22	16,04	0,675	0	61000	29000	21	2400	3	80	0	20	1,08	40,1	4,3
**C3-0.35-WRA19**	3754	3,6	47,6	20,22	16,04	0,675	0,1	61000	29000	21	2400	3	80	0	20	1,08	15,5	3,5
	3754	3,6	47,6	20,22	16,04	0,675	0,15	61000	29000	21	2400	3	80	0	20	1,08	16,4	1,7
	3754	3,6	47,6	20,22	16,04	0,675	0	63000	30000	21	2400	3	100	0	0	3,54	94,3	10,1
**C3-0.32-WRA20**	3754	3,6	47,6	20,22	16,04	0,675	0,1	63000	30000	21	2400	3	100	0	0	3,54	25,4	6,1
	3754	3,6	47,6	20,22	16,04	0,675	0,15	63000	30000	21	2400	3	100	0	0	3,54	14,6	3,8
	3754	3,6	47,6	20,22	16,04	0,675	0	63000	30000	21	2400	3	100	0	0	3,54	40,1	4,3
**C3-0.35-WRA20**	3754	3,6	47,6	20,22	16,04	0,675	0,1	63000	30000	21	2400	3	100	0	0	3,54	12,7	3,3
	3754	3,6	47,6	20,22	16,04	0,675	0,15	63000	30000	21	2400	3	100	0	0	3,54	3,8	1,2
	3754	3,6	47,6	20,22	16,04	0,675	0	56000	26500	21	2400	3	100	0	0	2,94	94,3	10,1
**C3-0.32-WRA21**	3754	3,6	47,6	20,22	16,04	0,675	0,1	56000	26500	21	2400	3	100	0	0	2,94	26,8	5,4
	3754	3,6	47,6	20,22	16,04	0,675	0,15	56000	26500	21	2400	3	100	0	0	2,94	6,2	2,2
	3754	3,6	47,6	20,22	16,04	0,675	0	56000	26500	21	2400	3	100	0	0	2,94	40,1	4,3
**C3-0.35-WRA21**	3754	3,6	47,6	20,22	16,04	0,675	0,1	56000	26500	21	2400	3	100	0	0	2,94	13,1	3
	3754	3,6	47,6	20,22	16,04	0,675	0,15	56000	26500	21	2400	3	100	0	0	2,94	3,6	1,1
	3754	3,6	47,6	20,22	16,04	0,675	0	60000	26000	21	2400	3	91	9	0	2,97	94,3	10,1
**C3-0.32-WRA22**	3754	3,6	47,6	20,22	16,04	0,675	0,1	60000	26000	21	2400	3	91	9	0	2,97	46,7	8
	3754	3,6	47,6	20,22	16,04	0,675	0,15	60000	26000	21	2400	3	91	9	0	2,97	38,5	5,9
	3754	3,6	47,6	20,22	16,04	0,675	0	60000	26000	21	2400	3	91	9	0	2,97	40,1	4,3
**C3-0.35-WRA22**	3754	3,6	47,6	20,22	16,04	0,675	0,1	60000	26000	21	2400	3	91	9	0	2,97	15,6	3,7
	3754	3,6	47,6	20,22	16,04	0,675	0,15	60000	26000	21	2400	3	91	9	0	2,97	9,5	2,2
	3754	3,6	47,6	20,22	16,04	0,675	0	54000	22500	21	2400	3	91	9	0	3,21	94,3	10,1
**C3-0.32-WRA23**	3754	3,6	47,6	20,22	16,04	0,675	0,1	54000	22500	21	2400	3	91	9	0	3,21	30,5	6
	3754	3,6	47,6	20,22	16,04	0,675	0,15	54000	22500	21	2400	3	91	9	0	3,21	18,3	4,1
**C3-0.35-WRA23**	3754	3,6	47,6	20,22	16,04	0,675	0	54000	22500	21	2400	3	91	9	0	3,21	40,1	4,3
	3754	3,6	47,6	20,22	16,04	0,675	0,1	54000	22500	21	2400	3	91	9	0	3,21	13,9	3,3

	3754	3,6	47,6	20,22	16,04	0,675	0,15	54000	22500	21	2400	3	91	9	0	3,21	12,3	2,9
**C3-0.32-WRA24**	3754	3,6	47,6	20,22	16,04	0,675	0	62000	29000	21	2400	3	91	0	9	3,24	94,3	10,1
	3754	3,6	47,6	20,22	16,04	0,675	0,1	62000	29000	21	2400	3	91	0	9	3,24	35,8	7,3
	3754	3,6	47,6	20,22	16,04	0,675	0,15	62000	29000	21	2400	3	91	0	9	3,24	11,2	3,1
**C3-0.35-WRA24**	3754	3,6	47,6	20,22	16,04	0,675	0	62000	29000	21	2400	3	91	0	9	3,24	40,1	4,3
	3754	3,6	47,6	20,22	16,04	0,675	0,1	62000	29000	21	2400	3	91	0	9	3,24	12,1	3,3
	3754	3,6	47,6	20,22	16,04	0,675	0,15	62000	29000	21	2400	3	91	0	9	3,24	7,0	1,8
**C3-0.32-WRA25**	3754	3,6	47,6	20,22	16,04	0,675	0	56000	28000	21	2400	3	91	0	9	3,58	94,3	10,1
	3754	3,6	47,6	20,22	16,04	0,675	0,1	56000	28000	21	2400	3	91	0	9	3,58	34,9	6,9
	3754	3,6	47,6	20,22	16,04	0,675	0,15	56000	28000	21	2400	3	91	0	9	3,58	25,5	4,8
**C3-0.35-WRA25**	3754	3,6	47,6	20,22	16,04	0,675	0	56000	28000	21	2400	3	91	0	9	3,58	40,1	4,3
	3754	3,6	47,6	20,22	16,04	0,675	0,1	56000	28000	21	2400	3	91	0	9	3,58	11,8	3,3
	3754	3,6	47,6	20,22	16,04	0,675	0,15	56000	28000	21	2400	3	91	0	9	3,58	7,6	1,1
**C6-0.32-WRA1**	3659	6,82	52,96	16,61	12,48	0,675	0	56000	26200	21	2400	3	100	0	0	3,97	117,9	10,1
	3659	6,82	52,96	16,61	12,48	0,675	0,1	56000	26200	21	2400	3	100	0	0	3,97	28,8	7,1
	3659	6,82	52,96	16,61	12,48	0,675	0,15	56000	26200	21	2400	3	100	0	0	3,97	9,3	3,1
**C6-0.35-WRA1**	3659	6,82	52,96	16,61	12,48	0,675	0	56000	26200	21	2400	3	100	0	0	3,97	46,1	4,8
	3659	6,82	52,96	16,61	12,48	0,675	0,1	56000	26200	21	2400	3	100	0	0	3,97	9,7	2,7
	3659	6,82	52,96	16,61	12,48	0,675	0,15	56000	26200	21	2400	3	100	0	0	3,97	4,2	1,3
**C6-0.32-WRA2**	3659	6,82	52,96	16,61	12,48	0,675	0	27000	11740	10	2400	3	100	0	0	2,87	117,9	10,1
	3659	6,82	52,96	16,61	12,48	0,675	0,1	27000	11740	10	2400	3	100	0	0	2,87	46,1	7,6
	3659	6,82	52,96	16,61	12,48	0,675	0,15	27000	11740	10	2400	3	100	0	0	2,87	49,0	6,9
**C6-0.35-WRA2**	3659	6,82	52,96	16,61	12,48	0,675	0	27000	11740	10	2400	3	100	0	0	2,87	46,1	4,8
	3659	6,82	52,96	16,61	12,48	0,675	0,1	27000	11740	10	2400	3	100	0	0	2,87	15,4	3,8
	3659	6,82	52,96	16,61	12,48	0,675	0,15	27000	11740	10	2400	3	100	0	0	2,87	13,6	2,8
**C6-0.32-WRA3**	3659	6,82	52,96	16,61	12,48	0,675	0	78000	37140	31	2400	3	100	0	0	3,26	117,9	10,1
	3659	6,82	52,96	16,61	12,48	0,675	0,1	78000	37140	31	2400	3	100	0	0	3,26	42,7	7,5
	3659	6,82	52,96	16,61	12,48	0,675	0,15	78000	37140	31	2400	3	100	0	0	3,26	45,6	6,7
**C6-0.35-WRA3**	3659	6,82	52,96	16,61	12,48	0,675	0	78000	37140	31	2400	3	100	0	0	3,26	46,1	4,8
	3659	6,82	52,96	16,61	12,48	0,675	0,1	78000	37140	31	2400	3	100	0	0	3,26	16,3	3,8
	3659	6,82	52,96	16,61	12,48	0,675	0,15	78000	37140	31	2400	3	100	0	0	3,26	14,5	2,8
**C6-0.32-WRA4**	3659	6,82	52,96	16,61	12,48	0,675	0	26000	11300	21	1000	3	100	0	0	3,16	117,9	10,1
	3659	6,82	52,96	16,61	12,48	0,675	0,1	26000	11300	21	1000	3	100	0	0	3,16	28,4	7,2

	3659	6,82	52,96	16,61	12,48	0,675	0,15	26000	11300	21	1000	3	100	0	0	3,16	17,4	4,9
	3659	6,82	52,96	16,61	12,48	0,675	0	26000	11300	21	1000	3	100	0	0	3,16	46,1	4,8
**C6-0.35-WRA4**	3659	6,82	52,96	16,61	12,48	0,675	0,1	26000	11300	21	1000	3	100	0	0	3,16	14,5	4,7
	3659	6,82	52,96	16,61	12,48	0,675	0,15	26000	11300	21	1000	3	100	0	0	3,16	11,9	1,6
	3659	6,82	52,96	16,61	12,48	0,675	0	69000	34500	21	3000	3	100	0	0	3,44	117,9	10,1
**C6-0.32-WRA5**	3659	6,82	52,96	16,61	12,48	0,675	0,1	69000	34500	21	3000	3	100	0	0	3,44	27,4	6,9
	3659	6,82	52,96	16,61	12,48	0,675	0,15	69000	34500	21	3000	3	100	0	0	3,44	12,9	4,1
	3659	6,82	52,96	16,61	12,48	0,675	0	69000	34500	21	3000	3	100	0	0	3,44	46,1	4,8
**C6-0.35-WRA5**	3659	6,82	52,96	16,61	12,48	0,675	0,1	69000	34500	21	3000	3	100	0	0	3,44	8,3	3,1
	3659	6,82	52,96	16,61	12,48	0,675	0,15	69000	34500	21	3000	3	100	0	0	3,44	3,1	0,9
	3659	6,82	52,96	16,61	12,48	0,675	0	56000	24340	40	1000	3	100	0	0	3,27	117,9	10,1
**C6-0.32-WRA6**	3659	6,82	52,96	16,61	12,48	0,675	0,1	56000	24340	40	1000	3	100	0	0	3,27	29,7	7,2
	3659	6,82	52,96	16,61	12,48	0,675	0,15	56000	24340	40	1000	3	100	0	0	3,27	14,5	4,3
	3659	6,82	52,96	16,61	12,48	0,675	0	56000	24340	40	1000	3	100	0	0	3,27	46,1	4,8
**C6-0.35-WRA6**	3659	6,82	52,96	16,61	12,48	0,675	0,1	56000	24340	40	1000	3	100	0	0	3,27	8,8	3,2
	3659	6,82	52,96	16,61	12,48	0,675	0,15	56000	24340	40	1000	3	100	0	0	3,27	3,1	1,3
	3659	6,82	52,96	16,61	12,48	0,675	0	57000	27140	17	3000	3	100	0	0	3,27	117,9	10,1
**C6-0.32-WRA7**	3659	6,82	52,96	16,61	12,48	0,675	0,1	57000	27140	17	3000	3	100	0	0	3,27	23,2	6,6
	3659	6,82	52,96	16,61	12,48	0,675	0,15	57000	27140	17	3000	3	100	0	0	3,27	12,1	3,5
	3659	6,82	52,96	16,61	12,48	0,675	0	57000	27140	17	3000	3	100	0	0	3,27	46,1	4,8
**C6-0.35-WRA7**	3659	6,82	52,96	16,61	12,48	0,675	0,1	57000	27140	17	3000	3	100	0	0	3,27	9,0	3
	3659	6,82	52,96	16,61	12,48	0,675	0,15	57000	27140	17	3000	3	100	0	0	3,27	5,4	1,6
	3659	6,82	52,96	16,61	12,48	0,675	0	58000	26300	21	2400	3	99	1	0	2,91	117,9	10,1
**C6-0.32-WRA8**	3659	6,82	52,96	16,61	12,48	0,675	0,1	58000	26300	21	2400	3	99	1	0	2,91	55,1	8,6
	3659	6,82	52,96	16,61	12,48	0,675	0,15	58000	26300	21	2400	3	99	1	0	2,91	46,1	7,2
	3659	6,82	52,96	16,61	12,48	0,675	0	58000	26300	21	2400	3	99	1	0	2,91	46,1	4,8
**C6-0.35-WRA8**	3659	6,82	52,96	16,61	12,48	0,675	0,1	58000	26300	21	2400	3	99	1	0	2,91	14,7	3,7
	3659	6,82	52,96	16,61	12,48	0,675	0,15	58000	26300	21	2400	3	99	1	0	2,91	14,2	1,5
	3659	6,82	52,96	16,61	12,48	0,675	0	65000	27000	21	2400	3	97	3	0	2,93	117,9	10,1
**C6-0.32-WRA9**	3659	6,82	52,96	16,61	12,48	0,675	0,1	65000	27000	21	2400	3	97	3	0	2,93	44,2	8
	3659	6,82	52,96	16,61	12,48	0,675	0,15	65000	27000	21	2400	3	97	3	0	2,93	33,6	6,2
**C6-0.35-WRA9**	3659	6,82	52,96	16,61	12,48	0,675	0	65000	27000	21	2400	3	97	3	0	2,93	46,1	4,8
	3659	6,82	52,96	16,61	12,48	0,675	0,1	65000	27000	21	2400	3	97	3	0	2,93	13,5	3,6

	3659	6,82	52,96	16,61	12,48	0,675	0,15	65000	27000	21	2400	3	97	3	0	2,93	13,6	1,6
	3659	6,82	52,96	16,61	12,48	0,675	0	61000	26500	21	2400	3	95	5	0	2,93	117,9	10,1
**C6-0.32-WRA10**	3659	6,82	52,96	16,61	12,48	0,675	0,1	61000	26500	21	2400	3	95	5	0	2,93	55,6	8,6
	3659	6,82	52,96	16,61	12,48	0,675	0,15	61000	26500	21	2400	3	95	5	0	2,93	31,9	5,8
	3659	6,82	52,96	16,61	12,48	0,675	0	61000	26500	21	2400	3	95	5	0	2,93	46,1	4,8
**C6-0.35-WRA10**	3659	6,82	52,96	16,61	12,48	0,675	0,1	61000	26500	21	2400	3	95	5	0	2,93	13,1	3,6
	3659	6,82	52,96	16,61	12,48	0,675	0,15	61000	26500	21	2400	3	95	5	0	2,93	11,6	1,5
	3659	6,82	52,96	16,61	12,48	0,675	0	56100	26600	21	2400	3	93	7	0	2,65	117,9	10,1
**C6-0.32-WRA11**	3659	6,82	52,96	16,61	12,48	0,675	0,1	56100	26600	21	2400	3	93	7	0	2,65	44,9	8
	3659	6,82	52,96	16,61	12,48	0,675	0,15	56100	26600	21	2400	3	93	7	0	2,65	27,7	5,8
	3659	6,82	52,96	16,61	12,48	0,675	0	56100	26600	21	2400	3	93	7	0	2,65	46,1	4,8
**C6-0.35-WRA11**	3659	6,82	52,96	16,61	12,48	0,675	0,1	56100	26600	21	2400	3	93	7	0	2,65	12,7	3,5
	3659	6,82	52,96	16,61	12,48	0,675	0,15	56100	26600	21	2400	3	93	7	0	2,65	11,2	1,5
	3659	6,82	52,96	16,61	12,48	0,675	0	53000	23000	21	2400	3	91	9	0	2,54	117,9	10,1
**C6-0.32-WRA12**	3659	6,82	52,96	16,61	12,48	0,675	0,1	53000	23000	21	2400	3	91	9	0	2,54	43,4	8
	3659	6,82	52,96	16,61	12,48	0,675	0,15	53000	23000	21	2400	3	91	9	0	2,54	36,6	6,5
	3659	6,82	52,96	16,61	12,48	0,675	0	53000	23000	21	2400	3	91	9	0	2,54	46,1	4,8
**C6-0.35-WRA12**	3659	6,82	52,96	16,61	12,48	0,675	0,1	53000	23000	21	2400	3	91	9	0	2,54	12,4	3,4
	3659	6,82	52,96	16,61	12,48	0,675	0,15	53000	23000	21	2400	3	91	9	0	2,54	12,5	1,5
	3659	6,82	52,96	16,61	12,48	0,675	0	52000	22600	21	2400	3	80	20	0	2,61	117,9	10,1
**C6-0.32-WRA13**	3659	6,82	52,96	16,61	12,48	0,675	0,1	52000	22600	21	2400	3	80	20	0	2,61	53,5	8,3
	3659	6,82	52,96	16,61	12,48	0,675	0,15	52000	22600	21	2400	3	80	20	0	2,61	41,1	6,8
	3659	6,82	52,96	16,61	12,48	0,675	0	52000	22600	21	2400	3	80	20	0	2,61	46,1	4,8
**C6-0.35-WRA13**	3659	6,82	52,96	16,61	12,48	0,675	0,1	52000	22600	21	2400	3	80	20	0	2,61	16,7	3,9
	3659	6,82	52,96	16,61	12,48	0,675	0,15	52000	22600	21	2400	3	80	20	0	2,61	14,2	1,7
	3659	6,82	52,96	16,61	12,48	0,675	0	62100	29500	21	2400	3	99	0	1	1,08	117,9	10,1
**C6-0.32-WRA14**	3659	6,82	52,96	16,61	12,48	0,675	0,1	62100	29500	21	2400	3	99	0	1	1,08	50,0	8,4
	3659	6,82	52,96	16,61	12,48	0,675	0,15	62100	29500	21	2400	3	99	0	1	1,08	35,3	6,7
	3659	6,82	52,96	16,61	12,48	0,675	0	62100	29500	21	2400	3	99	0	1	1,08	46,1	4,8
**C6-0.35-WRA14**	3659	6,82	52,96	16,61	12,48	0,675	0,1	62100	29500	21	2400	3	99	0	1	1,08	13,7	3,6
	3659	6,82	52,96	16,61	12,48	0,675	0,15	62100	29500	21	2400	3	99	0	1	1,08	15,7	2
**C6-0.32-WRA15**	3659	6,82	52,96	16,61	12,48	0,675	0	63000	28600	21	2400	3	97	0	3	1,08	117,9	10,1
	3659	6,82	52,96	16,61	12,48	0,675	0,1	63000	28600	21	2400	3	97	0	3	1,08	52,2	8,3

	3659	6,82	52,96	16,61	12,48	0,675	0,15	63000	28600	21	2400	3	97	0	3	1,08	33,3	6,1
	3659	6,82	52,96	16,61	12,48	0,675	0	63000	28600	21	2400	3	97	0	3	1,08	46,1	4,8
**C6-0.35-WRA15**	3659	6,82	52,96	16,61	12,48	0,675	0,1	63000	28600	21	2400	3	97	0	3	1,08	21,0	4
	3659	6,82	52,96	16,61	12,48	0,675	0,15	63000	28600	21	2400	3	97	0	3	1,08	16,1	2,2
	3659	6,82	52,96	16,61	12,48	0,675	0	58000	29000	21	2400	3	95	0	5	1,08	117,9	10,1
**C6-0.32-WRA16**	3659	6,82	52,96	16,61	12,48	0,675	0,1	58000	29000	21	2400	3	95	0	5	1,08	50,7	8,3
	3659	6,82	52,96	16,61	12,48	0,675	0,15	58000	29000	21	2400	3	95	0	5	1,08	36,1	6,6
	3659	6,82	52,96	16,61	12,48	0,675	0	58000	29000	21	2400	3	95	0	5	1,08	46,1	4,8
**C6-0.35-WRA16**	3659	6,82	52,96	16,61	12,48	0,675	0,1	58000	29000	21	2400	3	95	0	5	1,08	7,3	3,3
	3659	6,82	52,96	16,61	12,48	0,675	0,15	58000	29000	21	2400	3	95	0	5	1,08	15,9	1,8
	3659	6,82	52,96	16,61	12,48	0,675	0	63000	28600	21	2400	3	93	0	7	1,08	117,9	10,1
**C6-0.32-WRA17**	3659	6,82	52,96	16,61	12,48	0,675	0,1	63000	28600	21	2400	3	93	0	7	1,08	42,6	7,8
	3659	6,82	52,96	16,61	12,48	0,675	0,15	63000	28600	21	2400	3	93	0	7	1,08	34,1	6,3
	3659	6,82	52,96	16,61	12,48	0,675	0	63000	28600	21	2400	3	93	0	7	1,08	46,1	4,8
**C6-0.35-WRA17**	3659	6,82	52,96	16,61	12,48	0,675	0,1	63000	28600	21	2400	3	93	0	7	1,08	14,6	3,5
	3659	6,82	52,96	16,61	12,48	0,675	0,15	63000	28600	21	2400	3	93	0	7	1,08	17,6	2,2
	3659	6,82	52,96	16,61	12,48	0,675	0	60000	25000	21	2400	3	91	0	9	1,08	117,9	10,1
**C6-0.32-WRA18**	3659	6,82	52,96	16,61	12,48	0,675	0,1	60000	25000	21	2400	3	91	0	9	1,08	51,5	8,2
	3659	6,82	52,96	16,61	12,48	0,675	0,15	60000	25000	21	2400	3	91	0	9	1,08	46,9	6,8
	3659	6,82	52,96	16,61	12,48	0,675	0	60000	25000	21	2400	3	91	0	9	1,08	46,1	4,8
**C6-0.35-WRA18**	3659	6,82	52,96	16,61	12,48	0,675	0,1	60000	25000	21	2400	3	91	0	9	1,08	15,1	3,7
	3659	6,82	52,96	16,61	12,48	0,675	0,15	60000	25000	21	2400	3	91	0	9	1,08	15,2	2,6
	3659	6,82	52,96	16,61	12,48	0,675	0	61000	29000	21	2400	3	80	0	20	1,08	117,9	10,1
**C6-0.32-WRA19**	3659	6,82	52,96	16,61	12,48	0,675	0,1	61000	29000	21	2400	3	80	0	20	1,08	58,9	8,8
	3659	6,82	52,96	16,61	12,48	0,675	0,15	61000	29000	21	2400	3	80	0	20	1,08	46,5	7,3
	3659	6,82	52,96	16,61	12,48	0,675	0	61000	29000	21	2400	3	80	0	20	1,08	46,1	4,8
**C6-0.35-WRA19**	3659	6,82	52,96	16,61	12,48	0,675	0,1	61000	29000	21	2400	3	80	0	20	1,08	15,8	3,5
	3659	6,82	52,96	16,61	12,48	0,675	0,15	61000	29000	21	2400	3	80	0	20	1,08	20,2	2,3
	3659	6,82	52,96	16,61	12,48	0,675	0	63000	30000	21	2400	3	100	0	0	3,54	117,9	10,1
**C6-0.32-WRA20**	3659	6,82	52,96	16,61	12,48	0,675	0,1	63000	30000	21	2400	3	100	0	0	3,54	32,8	6,7
	3659	6,82	52,96	16,61	12,48	0,675	0,15	63000	30000	21	2400	3	100	0	0	3,54	13,3	3,5
**C6-0.35-WRA20**	3659	6,82	52,96	16,61	12,48	0,675	0	63000	30000	21	2400	3	100	0	0	3,54	46,1	4,8
	3659	6,82	52,96	16,61	12,48	0,675	0,1	63000	30000	21	2400	3	100	0	0	3,54	13,3	3,2

	3659	6,82	52,96	16,61	12,48	0,675	0,15	63000	30000	21	2400	3	100	0	0	3,54	14,8	2
	3659	6,82	52,96	16,61	12,48	0,675	0	56000	26500	21	2400	3	100	0	0	2,94	117,9	10,1
**C6-0.32-WRA21**	3659	6,82	52,96	16,61	12,48	0,675	0,1	56000	26500	21	2400	3	100	0	0	2,94	23,8	6
	3659	6,82	52,96	16,61	12,48	0,675	0,15	56000	26500	21	2400	3	100	0	0	2,94	17,7	4,7
	3659	6,82	52,96	16,61	12,48	0,675	0	56000	26500	21	2400	3	100	0	0	2,94	46,1	4,8
**C6-0.35-WRA21**	3659	6,82	52,96	16,61	12,48	0,675	0,1	56000	26500	21	2400	3	100	0	0	2,94	10,9	2,8
	3659	6,82	52,96	16,61	12,48	0,675	0,15	56000	26500	21	2400	3	100	0	0	2,94	3,8	1
	3659	6,82	52,96	16,61	12,48	0,675	0	60000	26000	21	2400	3	91	9	0	2,97	117,9	10,1
**C6-0.32-WRA22**	3659	6,82	52,96	16,61	12,48	0,675	0,1	60000	26000	21	2400	3	91	9	0	2,97	33,9	6,8
	3659	6,82	52,96	16,61	12,48	0,675	0,15	60000	26000	21	2400	3	91	9	0	2,97	34,5	6,1
	3659	6,82	52,96	16,61	12,48	0,675	0	60000	26000	21	2400	3	91	9	0	2,97	46,1	4,8
**C6-0.35-WRA22**	3659	6,82	52,96	16,61	12,48	0,675	0,1	60000	26000	21	2400	3	91	9	0	2,97	12,3	3,2
	3659	6,82	52,96	16,61	12,48	0,675	0,15	60000	26000	21	2400	3	91	9	0	2,97	21,3	2,4
	3659	6,82	52,96	16,61	12,48	0,675	0	54000	22500	21	2400	3	91	9	0	3,21	117,9	10,1
**C6-0.32-WRA23**	3659	6,82	52,96	16,61	12,48	0,675	0,1	54000	22500	21	2400	3	91	9	0	3,21	28,7	6,4
	3659	6,82	52,96	16,61	12,48	0,675	0,15	54000	22500	21	2400	3	91	9	0	3,21	19,0	4,5
	3659	6,82	52,96	16,61	12,48	0,675	0	54000	22500	21	2400	3	91	9	0	3,21	46,1	4,8
**C6-0.35-WRA23**	3659	6,82	52,96	16,61	12,48	0,675	0,1	54000	22500	21	2400	3	91	9	0	3,21	12,3	3,1
	3659	6,82	52,96	16,61	12,48	0,675	0,15	54000	22500	21	2400	3	91	9	0	3,21	5,9	1,6
	3659	6,82	52,96	16,61	12,48	0,675	0	62000	29000	21	2400	3	91	0	9	3,24	117,9	10,1
**C6-0.32-WRA24**	3659	6,82	52,96	16,61	12,48	0,675	0,1	62000	29000	21	2400	3	91	0	9	3,24	24,9	6,2
	3659	6,82	52,96	16,61	12,48	0,675	0,15	62000	29000	21	2400	3	91	0	9	3,24	8,6	3,1
	3659	6,82	52,96	16,61	12,48	0,675	0	62000	29000	21	2400	3	91	0	9	3,24	46,1	4,8
**C6-0.35-WRA24**	3659	6,82	52,96	16,61	12,48	0,675	0,1	62000	29000	21	2400	3	91	0	9	3,24	11,0	3
	3659	6,82	52,96	16,61	12,48	0,675	0,15	62000	29000	21	2400	3	91	0	9	3,24	7,9	2,1
	3659	6,82	52,96	16,61	12,48	0,675	0	56000	28000	21	2400	3	91	0	9	3,58	117,9	10,1
**C6-0.32-WRA25**	3659	6,82	52,96	16,61	12,48	0,675	0,1	56000	28000	21	2400	3	91	0	9	3,58	28,6	6
	3659	6,82	52,96	16,61	12,48	0,675	0,15	56000	28000	21	2400	3	91	0	9	3,58	28,1	2,8
	3659	6,82	52,96	16,61	12,48	0,675	0	56000	28000	21	2400	3	91	0	9	3,58	46,1	4,8
**C6-0.35-WRA25**	3659	6,82	52,96	16,61	12,48	0,675	0,1	56000	28000	21	2400	3	91	0	9	3,58	7,5	2,6
	3659	6,82	52,96	16,61	12,48	0,675	0,15	56000	28000	21	2400	3	91	0	9	3,58	2,3	0,9
**C9-0.32-WRA1**	4259	9,05	48,42	21,25	10,07	0,675	0	56000	26200	21	2400	3	100	0	0	3,97	124,8	17,4
	4259	9,05	48,42	21,25	10,07	0,675	0,1	56000	26200	21	2400	3	100	0	0	3,97	76,1	11,9

	4259	9,05	48,42	21,25	10,07	0,675	0,15	56000	26200	21	2400	3	100	0	0	3,97	85,0	13,2
	4259	9,05	48,42	21,25	10,07	0,675	0	56000	26200	21	2400	3	100	0	0	3,97	116,7	9
**C9-0.35-WRA1**	4259	9,05	48,42	21,25	10,07	0,675	0,1	56000	26200	21	2400	3	100	0	0	3,97	62,6	8,8
	4259	9,05	48,42	21,25	10,07	0,675	0,15	56000	26200	21	2400	3	100	0	0	3,97	37,1	6,1
	4259	9,05	48,42	21,25	10,07	0,675	0	27000	11740	10	2400	3	100	0	0	2,87	124,8	17,4
**C9-0.32-WRA2**	4259	9,05	48,42	21,25	10,07	0,675	0,1	27000	11740	10	2400	3	100	0	0	2,87	72,6	11,5
	4259	9,05	48,42	21,25	10,07	0,675	0,15	27000	11740	10	2400	3	100	0	0	2,87	84,3	11,6
	4259	9,05	48,42	21,25	10,07	0,675	0	27000	11740	10	2400	3	100	0	0	2,87	116,7	9
**C9-0.35-WRA2**	4259	9,05	48,42	21,25	10,07	0,675	0,1	27000	11740	10	2400	3	100	0	0	2,87	58,2	9
	4259	9,05	48,42	21,25	10,07	0,675	0,15	27000	11740	10	2400	3	100	0	0	2,87	42,0	8,3
	4259	9,05	48,42	21,25	10,07	0,675	0	78000	37140	31	2400	3	100	0	0	3,26	124,8	17,4
**C9-0.32-WRA3**	4259	9,05	48,42	21,25	10,07	0,675	0,1	78000	37140	31	2400	3	100	0	0	3,26	62,8	10,6
	4259	9,05	48,42	21,25	10,07	0,675	0,15	78000	37140	31	2400	3	100	0	0	3,26	64,8	10,9
	4259	9,05	48,42	21,25	10,07	0,675	0	78000	37140	31	2400	3	100	0	0	3,26	116,7	9
**C9-0.35-WRA3**	4259	9,05	48,42	21,25	10,07	0,675	0,1	78000	37140	31	2400	3	100	0	0	3,26	68,1	9,3
	4259	9,05	48,42	21,25	10,07	0,675	0,15	78000	37140	31	2400	3	100	0	0	3,26	58,3	9,3
	4259	9,05	48,42	21,25	10,07	0,675	0	26000	11300	21	1000	3	100	0	0	3,16	124,8	17,4
**C9-0.32-WRA4**	4259	9,05	48,42	21,25	10,07	0,675	0,1	26000	11300	21	1000	3	100	0	0	3,16	71,2	12,5
	4259	9,05	48,42	21,25	10,07	0,675	0,15	26000	11300	21	1000	3	100	0	0	3,16	93,7	11,7
	4259	9,05	48,42	21,25	10,07	0,675	0	26000	11300	21	1000	3	100	0	0	3,16	116,7	9
**C9-0.35-WRA4**	4259	9,05	48,42	21,25	10,07	0,675	0,1	26000	11300	21	1000	3	100	0	0	3,16	54,9	8,2
	4259	9,05	48,42	21,25	10,07	0,675	0,15	26000	11300	21	1000	3	100	0	0	3,16	43,9	6,1
	4259	9,05	48,42	21,25	10,07	0,675	0	69000	34500	21	3000	3	100	0	0	3,44	124,8	17,4
**C9-0.32-WRA5**	4259	9,05	48,42	21,25	10,07	0,675	0,1	69000	34500	21	3000	3	100	0	0	3,44	91,6	13,4
	4259	9,05	48,42	21,25	10,07	0,675	0,15	69000	34500	21	3000	3	100	0	0	3,44	113,4	13,6
	4259	9,05	48,42	21,25	10,07	0,675	0	69000	34500	21	3000	3	100	0	0	3,44	116,7	9
**C9-0.35-WRA5**	4259	9,05	48,42	21,25	10,07	0,675	0,1	69000	34500	21	3000	3	100	0	0	3,44	58,9	8,5
	4259	9,05	48,42	21,25	10,07	0,675	0,15	69000	34500	21	3000	3	100	0	0	3,44	47,0	6,8
	4259	9,05	48,42	21,25	10,07	0,675	0	56000	24340	40	1000	3	100	0	0	3,27	124,8	17,4
**C9-0.32-WRA6**	4259	9,05	48,42	21,25	10,07	0,675	0,1	56000	24340	40	1000	3	100	0	0	3,27	54,9	10,2
	4259	9,05	48,42	21,25	10,07	0,675	0,15	56000	24340	40	1000	3	100	0	0	3,27	79,0	10,8
**C9-0.35-WRA6**	4259	9,05	48,42	21,25	10,07	0,675	0	56000	24340	40	1000	3	100	0	0	3,27	116,7	9
	4259	9,05	48,42	21,25	10,07	0,675	0,1	56000	24340	40	1000	3	100	0	0	3,27	52,8	7,6

	4259	9,05	48,42	21,25	10,07	0,675	0,15	56000	24340	40	1000	3	100	0	0	3,27	41,1	6,1
**C9-0.32-WRA7**	4259	9,05	48,42	21,25	10,07	0,675	0	57000	27140	17	3000	3	100	0	0	3,27	124,8	17,4
	4259	9,05	48,42	21,25	10,07	0,675	0,1	57000	27140	17	3000	3	100	0	0	3,27	96,8	12,7
	4259	9,05	48,42	21,25	10,07	0,675	0,15	57000	27140	17	3000	3	100	0	0	3,27	118,3	13,8
**C9-0.35-WRA7**	4259	9,05	48,42	21,25	10,07	0,675	0	57000	27140	17	3000	3	100	0	0	3,27	116,7	9
	4259	9,05	48,42	21,25	10,07	0,675	0,1	57000	27140	17	3000	3	100	0	0	3,27	52,2	8,5
	4259	9,05	48,42	21,25	10,07	0,675	0,15	57000	27140	17	3000	3	100	0	0	3,27	56,0	7,4
**C9-0.32-WRA8**	4259	9,05	48,42	21,25	10,07	0,675	0	58000	26300	21	2400	3	99	1	0	2,91	124,8	17,4
	4259	9,05	48,42	21,25	10,07	0,675	0,1	58000	26300	21	2400	3	99	1	0	2,91	114,4	10,8
	4259	9,05	48,42	21,25	10,07	0,675	0,15	58000	26300	21	2400	3	99	1	0	2,91	144,8	13
**C9-0.35-WRA8**	4259	9,05	48,42	21,25	10,07	0,675	0	58000	26300	26300	2400	3	99	1	0	2,91	116,7	9
	4259	9,05	48,42	21,25	10,07	0,675	0,1	58000	26300	26300	2400	3	99	1	0	2,91	50,2	7,4
	4259	9,05	48,42	21,25	10,07	0,675	0,15	58000	26300	26300	2400	3	99	1	0	2,91	38,0	5,8
**C9-0.32-WRA9**	4259	9,05	48,42	21,25	10,07	0,675	0	65000	27000	27000	2400	3	97	3	0	2,93	124,8	17,4
	4259	9,05	48,42	21,25	10,07	0,675	0,1	65000	27000	27000	2400	3	97	3	0	2,93	90,1	10,2
	4259	9,05	48,42	21,25	10,07	0,675	0,15	65000	27000	27000	2400	3	97	3	0	2,93	131,1	13,3
**C9-0.35-WRA9**	4259	9,05	48,42	21,25	10,07	0,675	0	65000	27000	27000	2400	3	97	3	0	2,93	116,7	9
	4259	9,05	48,42	21,25	10,07	0,675	0,1	65000	27000	27000	2400	3	97	3	0	2,93	42,5	7,5
	4259	9,05	48,42	21,25	10,07	0,675	0,15	65000	27000	27000	2400	3	97	3	0	2,93	32,3	5,9
**C9-0.32-WRA10**	4259	9,05	48,42	21,25	10,07	0,675	0	61000	26500	26500	2400	3	95	5	0	2,93	124,8	17,4
	4259	9,05	48,42	21,25	10,07	0,675	0,1	61000	26500	26500	2400	3	95	5	0	2,93	100,4	10,8
	4259	9,05	48,42	21,25	10,07	0,675	0,15	61000	26500	26500	2400	3	95	5	0	2,93	95,8	12
**C9-0.35-WRA10**	4259	9,05	48,42	21,25	10,07	0,675	0	61000	26500	26500	2400	3	95	5	0	2,93	116,7	9
	4259	9,05	48,42	21,25	10,07	0,675	0,1	61000	26500	26500	2400	3	95	5	0	2,93	50,3	7,4
	4259	9,05	48,42	21,25	10,07	0,675	0,15	61000	26500	26500	2400	3	95	5	0	2,93	31,3	5,9
**C9-0.32-WRA11**	4259	9,05	48,42	21,25	10,07	0,675	0	56100	26600	26600	2400	3	93	7	0	2,65	124,8	17,4
	4259	9,05	48,42	21,25	10,07	0,675	0,1	56100	26600	26600	2400	3	93	7	0	2,65	79,9	10,1
	4259	9,05	48,42	21,25	10,07	0,675	0,15	56100	26600	26600	2400	3	93	7	0	2,65	117,4	12,1
**C9-0.35-WRA11**	4259	9,05	48,42	21,25	10,07	0,675	0	56100	26600	26600	2400	3	93	7	0	2,65	116,7	9
	4259	9,05	48,42	21,25	10,07	0,675	0,1	56100	26600	26600	2400	3	93	7	0	2,65	52,6	7,4
	4259	9,05	48,42	21,25	10,07	0,675	0,15	56100	26600	26600	2400	3	93	7	0	2,65	36,2	6,1
**C9-0.32-WRA12**	4259	9,05	48,42	21,25	10,07	0,675	0	53000	23000	23000	2400	3	91	9	0	2,54	124,8	17,4
	4259	9,05	48,42	21,25	10,07	0,675	0,1	53000	23000	23000	2400	3	91	9	0	2,54	56,4	9,3

	4259	9,05	48,42	21,25	10,07	0,675	0,15	53000	23000	23000	2400	3	91	9	0	2,54	82,3	11,5
**C9-0.35-WRA12**	4259	9,05	48,42	21,25	10,07	0,675	0	53000	23000	23000	2400	3	91	9	0	2,54	116,7	9
	4259	9,05	48,42	21,25	10,07	0,675	0,1	53000	23000	23000	2400	3	91	9	0	2,54	46,6	7,3
	4259	9,05	48,42	21,25	10,07	0,675	0,15	53000	23000	23000	2400	3	91	9	0	2,54	28,2	5,7
**C9-0.32-WRA13**	4259	9,05	48,42	21,25	10,07	0,675	0	52000	22600	22600	2400	3	80	20	0	2,61	124,8	17,4
	4259	9,05	48,42	21,25	10,07	0,675	0,1	52000	22600	22600	2400	3	80	20	0	2,61	72,5	9,5
	4259	9,05	48,42	21,25	10,07	0,675	0,15	52000	22600	22600	2400	3	80	20	0	2,61	89,1	11,6
**C9-0.35-WRA13**	4259	9,05	48,42	21,25	10,07	0,675	0	52000	22600	22600	2400	3	80	20	0	2,61	116,7	9
	4259	9,05	48,42	21,25	10,07	0,675	0,1	52000	22600	22600	2400	3	80	20	0	2,61	57,4	7,6
	4259	9,05	48,42	21,25	10,07	0,675	0,15	52000	22600	22600	2400	3	80	20	0	2,61	46,5	6,3
**C9-0.32-WRA14**	4259	9,05	48,42	21,25	10,07	0,675	0	62100	29500	29500	2400	3	99	0	1	1,08	124,8	17,4
	4259	9,05	48,42	21,25	10,07	0,675	0,1	62100	29500	29500	2400	3	99	0	1	1,08	77,5	9,9
	4259	9,05	48,42	21,25	10,07	0,675	0,15	62100	29500	29500	2400	3	99	0	1	1,08	77,6	12,4
**C9-0.35-WRA14**	4259	9,05	48,42	21,25	10,07	0,675	0	62100	29500	29500	2400	3	99	0	1	1,08	116,7	9
	4259	9,05	48,42	21,25	10,07	0,675	0,1	62100	29500	29500	2400	3	99	0	1	1,08	48,0	7
	4259	9,05	48,42	21,25	10,07	0,675	0,15	62100	29500	29500	2400	3	99	0	1	1,08	31,3	5,2
**C9-0.32-WRA15**	4259	9,05	48,42	21,25	10,07	0,675	0	63000	28600	28600	2400	3	97	0	3	1,08	124,8	17,4
	4259	9,05	48,42	21,25	10,07	0,675	0,1	63000	28600	28600	2400	3	97	0	3	1,08	89,4	17,4
	4259	9,05	48,42	21,25	10,07	0,675	0,15	63000	28600	28600	2400	3	97	0	3	1,08	126,6	13,5
**C9-0.35-WRA15**	4259	9,05	48,42	21,25	10,07	0,675	0	63000	28600	28600	2400	3	97	0	3	1,08	116,7	9
	4259	9,05	48,42	21,25	10,07	0,675	0,1	63000	28600	28600	2400	3	97	0	3	1,08	76,5	7,8
	4259	9,05	48,42	21,25	10,07	0,675	0,15	63000	28600	28600	2400	3	97	0	3	1,08	38,4	6,4
**C9-0.32-WRA16**	4259	9,05	48,42	21,25	10,07	0,675	0	58000	29000	29000	2400	3	95	0	5	1,08	124,8	17,4
	4259	9,05	48,42	21,25	10,07	0,675	0,1	58000	29000	29000	2400	3	95	0	5	1,08	59,3	9,9
	4259	9,05	48,42	21,25	10,07	0,675	0,15	58000	29000	29000	2400	3	95	0	5	1,08	81,3	12,2
**C9-0.35-WRA16**	4259	9,05	48,42	21,25	10,07	0,675	0	58000	29000	29000	2400	3	95	0	5	1,08	116,7	9
	4259	9,05	48,42	21,25	10,07	0,675	0,1	58000	29000	29000	2400	3	95	0	5	1,08	60,6	7,2
	4259	9,05	48,42	21,25	10,07	0,675	0,15	58000	29000	29000	2400	3	95	0	5	1,08	25,4	4,1
**C9-0.32-WRA17**	4259	9,05	48,42	21,25	10,07	0,675	0	63000	28600	28600	2400	3	93	0	7	1,08	124,8	17,4
	4259	9,05	48,42	21,25	10,07	0,675	0,1	63000	28600	28600	2400	3	93	0	7	1,08	64,8	9,8
	4259	9,05	48,42	21,25	10,07	0,675	0,15	63000	28600	28600	2400	3	93	0	7	1,08	79,3	11,2
**C9-0.35-WRA17**	4259	9,05	48,42	21,25	10,07	0,675	0	63000	28600	28600	2400	3	93	0	7	1,08	116,7	9
	4259	9,05	48,42	21,25	10,07	0,675	0,1	63000	28600	28600	2400	3	93	0	7	1,08	48,7	6,9

	4259	9,05	48,42	21,25	10,07	0,675	0,15	63000	28600	28600	2400	3	93	0	7	1,08	33,4	4,6
**C9-0.32-WRA18**	4259	9,05	48,42	21,25	10,07	0,675	0	60000	25000	25000	2400	3	91	0	9	1,08	124,8	17,4
	4259	9,05	48,42	21,25	10,07	0,675	0,1	60000	25000	25000	2400	3	91	0	9	1,08	66,7	9,8
	4259	9,05	48,42	21,25	10,07	0,675	0,15	60000	25000	25000	2400	3	91	0	9	1,08	104,9	11,9
**C9-0.35-WRA18**	4259	9,05	48,42	21,25	10,07	0,675	0	60000	25000	25000	2400	3	91	0	9	1,08	116,7	9
	4259	9,05	48,42	21,25	10,07	0,675	0,1	60000	25000	25000	2400	3	91	0	9	1,08	78,7	7,8
	4259	9,05	48,42	21,25	10,07	0,675	0,15	60000	25000	25000	2400	3	91	0	9	1,08	39,7	5,6
**C9-0.32-WRA19**	4259	9,05	48,42	21,25	10,07	0,675	0	61000	29000	29000	2400	3	80	0	20	1,08	124,8	17,4
	4259	9,05	48,42	21,25	10,07	0,675	0,1	61000	29000	29000	2400	3	80	0	20	1,08	64,0	9,9
	4259	9,05	48,42	21,25	10,07	0,675	0,15	61000	29000	29000	2400	3	80	0	20	1,08	99,4	12,4
**C9-0.35-WRA19**	4259	9,05	48,42	21,25	10,07	0,675	0	61000	29000	29000	2400	3	80	0	20	1,08	116,7	9
	4259	9,05	48,42	21,25	10,07	0,675	0,1	61000	29000	29000	2400	3	80	0	20	1,08	81,7	7,9
	4259	9,05	48,42	21,25	10,07	0,675	0,15	61000	29000	29000	2400	3	80	0	20	1,08	38,3	5,8
**C9-0.32-WRA20**	4259	9,05	48,42	21,25	10,07	0,675	0	63000	30000	30000	2400	3	100	0	0	3,54	124,8	17,4
	4259	9,05	48,42	21,25	10,07	0,675	0,1	63000	30000	30000	2400	3	100	0	0	3,54	92,6	13
	4259	9,05	48,42	21,25	10,07	0,675	0,15	63000	30000	30000	2400	3	100	0	0	3,54	222,4	18,7
**C9-0.35-WRA20**	4259	9,05	48,42	21,25	10,07	0,675	0	63000	30000	30000	2400	3	100	0	0	3,54	116,7	9
	4259	9,05	48,42	21,25	10,07	0,675	0,1	63000	30000	30000	2400	3	100	0	0	3,54	58,5	9,2
	4259	9,05	48,42	21,25	10,07	0,675	0,15	63000	30000	30000	2400	3	100	0	0	3,54	61,3	7,3
**C9-0.32-WRA21**	4259	9,05	48,42	21,25	10,07	0,675	0	56000	26500	26500	2400	3	100	0	0	2,94	124,8	17,4
	4259	9,05	48,42	21,25	10,07	0,675	0,1	56000	26500	26500	2400	3	100	0	0	2,94	40,9	11,2
	4259	9,05	48,42	21,25	10,07	0,675	0,15	56000	26500	26500	2400	3	100	0	0	2,94	58,3	11,5
**C9-0.35-WRA21**	4259	9,05	48,42	21,25	10,07	0,675	0	56000	26500	26500	2400	3	100	0	0	2,94	116,7	9
	4259	9,05	48,42	21,25	10,07	0,675	0,1	56000	26500	26500	2400	3	100	0	0	2,94	60,7	8,5
	4259	9,05	48,42	21,25	10,07	0,675	0,15	56000	26500	26500	2400	3	100	0	0	2,94	36,2	6
**C9-0.32-WRA22**	4259	9,05	48,42	21,25	10,07	0,675	0	60000	26000	26000	2400	3	91	9	0	2,97	124,8	17,4
	4259	9,05	48,42	21,25	10,07	0,675	0,1	60000	26000	26000	2400	3	91	9	0	2,97	59,2	8,2
	4259	9,05	48,42	21,25	10,07	0,675	0,15	60000	26000	26000	2400	3	91	9	0	2,97	138,1	14,3
**C9-0.35-WRA22**	4259	9,05	48,42	21,25	10,07	0,675	0	60000	26000	26000	2400	3	91	9	0	2,97	116,7	9
	4259	9,05	48,42	21,25	10,07	0,675	0,1	60000	26000	26000	2400	3	91	9	0	2,97	59,4	9,1
	4259	9,05	48,42	21,25	10,07	0,675	0,15	60000	26000	26000	2400	3	91	9	0	2,97	44,2	8
**C9-0.32-WRA23**	4259	9,05	48,42	21,25	10,07	0,675	0	54000	22500	22500	2400	3	91	9	0	3,21	124,8	17,4
	4259	9,05	48,42	21,25	10,07	0,675	0,1	54000	22500	22500	2400	3	91	9	0	3,21	137,6	14,6

	4259	9,05	48,42	21,25	10,07	0,675	0,15	54000	22500	22500	2400	3	91	9	0	3,21	85,8	12,9
	4259	9,05	48,42	21,25	10,07	0,675	0	54000	22500	22500	2400	3	91	9	0	3,21	116,7	9
**C9-0.35-WRA23**	4259	9,05	48,42	21,25	10,07	0,675	0,1	54000	22500	22500	2400	3	91	9	0	3,21	50,3	8,9
	4259	9,05	48,42	21,25	10,07	0,675	0,15	54000	22500	22500	2400	3	91	9	0	3,21	44,2	6,4
	4259	9,05	48,42	21,25	10,07	0,675	0	62000	29000	29000	2400	3	91	0	9	3,24	124,8	17,4
**C9-0.32-WRA24**	4259	9,05	48,42	21,25	10,07	0,675	0,1	62000	29000	29000	2400	3	91	0	9	3,24	66,0	9,9
	4259	9,05	48,42	21,25	10,07	0,675	0,15	62000	29000	29000	2400	3	91	0	9	3,24	119,6	13,7
	4259	9,05	48,42	21,25	10,07	0,675	0	62000	29000	29000	2400	3	91	0	9	3,24	116,7	9
**C9-0.35-WRA24**	4259	9,05	48,42	21,25	10,07	0,675	0,1	62000	29000	29000	2400	3	91	0	9	3,24	56,0	8,4
	4259	9,05	48,42	21,25	10,07	0,675	0,15	62000	29000	29000	2400	3	91	0	9	3,24	48,8	6,7
	4259	9,05	48,42	21,25	10,07	0,675	0	56000	28000	28000	2400	3	91	0	9	3,58	124,8	17,4
**C9-0.32-WRA25**	4259	9,05	48,42	21,25	10,07	0,675	0,1	56000	28000	28000	2400	3	91	0	9	3,58	74,2	14,3
	4259	9,05	48,42	21,25	10,07	0,675	0,15	56000	28000	28000	2400	3	91	0	9	3,58	137,4	15,9
	4259	9,05	48,42	21,25	10,07	0,675	0	56000	28000	28000	2400	3	91	0	9	3,58	116,7	9
**C9-0.35-WRA25**	4259	9,05	48,42	21,25	10,07	0,675	0,1	56000	28000	28000	2400	3	91	0	9	3,58	51,1	7,8
	4259	9,05	48,42	21,25	10,07	0,675	0,15	56000	28000	28000	2400	3	91	0	9	3,58	41,4	6
	3600	9,1	64,5	-	9,3	0,765	0	56000	26200	26200	2400	3	100	0	0	3,97	81,9	7
**CEM I-0.32-WRA1**	3600	9,1	64,5	-	9,3	0,765	0,1	56000	26200	26200	2400	3	100	0	0	3,97	43,1	6,3
	3600	9,1	64,5	-	9,3	0,765	0,15	56000	26200	26200	2400	3	100	0	0	3,97	22,7	4,1
	3600	9,1	64,5	-	9,3	0,765	0	56000	26200	26200	2400	3	100	0	0	3,97	57,2	4,1
**CEM I-0.35-WRA1**	3600	9,1	64,5	-	9,3	0,765	0,1	56000	26200	26200	2400	3	100	0	0	3,97	16,7	2,9
	3600	9,1	64,5	-	9,3	0,765	0,15	56000	26200	26200	2400	3	100	0	0	3,97	8,1	2,1
	3600	9,1	64,5	-	9,3	0,765	0	27000	11740	11740	2400	3	100	0	0	2,87	81,9	7
**CEM I-0.32-WRA2**	3600	9,1	64,5	-	9,3	0,765	0,1	27000	11740	11740	2400	3	100	0	0	2,87	49,8	6,7
	3600	9,1	64,5	-	9,3	0,765	0,15	27000	11740	11740	2400	3	100	0	0	2,87	43,4	6,7
	3600	9,1	64,5	-	9,3	0,765	0	27000	11740	11740	2400	3	100	0	0	2,87	57,2	4,1
**CEM I-0.35-WRA2**	3600	9,1	64,5	-	9,3	0,765	0,1	27000	11740	11740	2400	3	100	0	0	2,87	19,5	4,7
	3600	9,1	64,5	-	9,3	0,765	0,15	27000	11740	11740	2400	3	100	0	0	2,87	17,3	2,6
	3600	9,1	64,5	-	9,3	0,765	0	78000	37140	37140	2400	3	100	0	0	3,26	81,9	7
**CEM I-0.32-WRA3**	3600	9,1	64,5	-	9,3	0,765	0,1	78000	37140	37140	2400	3	100	0	0	3,26	56,0	7,9
	3600	9,1	64,5	-	9,3	0,765	0,15	78000	37140	37140	2400	3	100	0	0	3,26	35,4	5,7
**CEM I-0.35-WRA3**	3600	9,1	64,5	-	9,3	0,765	0	78000	37140	37140	2400	3	100	0	0	3,26	57,2	4,1
	3600	9,1	64,5	-	9,3	0,765	0,1	78000	37140	37140	2400	3	100	0	0	3,26	25,5	3,5

	3600	9,1	64,5	-	9,3	0,765	0,15	78000	37140	37140	2400	3	100	0	0	3,26	16,2	2,8
**CEM I-0.32-WRA4**	3600	9,1	64,5	-	9,3	0,765	0	26000	11300	11300	1000	3	100	0	0	3,16	81,9	7
	3600	9,1	64,5	-	9,3	0,765	0,1	26000	11300	11300	1000	3	100	0	0	3,16	47,6	6,5
	3600	9,1	64,5	-	9,3	0,765	0,15	26000	11300	11300	1000	3	100	0	0	3,16	29,1	4,5
**CEM I-0.35-WRA4**	3600	9,1	64,5	-	9,3	0,765	0	26000	11300	11300	1000	3	100	0	0	3,16	57,2	4,1
	3600	9,1	64,5	-	9,3	0,765	0,1	26000	11300	11300	1000	3	100	0	0	3,16	16,5	3,2
	3600	9,1	64,5	-	9,3	0,765	0,15	26000	11300	11300	1000	3	100	0	0	3,16	10,9	2,8
**CEM I-0.32-WRA5**	3600	9,1	64,5	-	9,3	0,765	0	69000	34500	34500	3000	3	100	0	0	3,44	81,9	7
	3600	9,1	64,5	-	9,3	0,765	0,1	69000	34500	34500	3000	3	100	0	0	3,44	50,2	6,7
	3600	9,1	64,5	-	9,3	0,765	0,15	69000	34500	34500	3000	3	100	0	0	3,44	25,0	4,5
**CEM I-0.35-WRA5**	3600	9,1	64,5	-	9,3	0,765	0	69000	34500	34500	3000	3	100	0	0	3,44	57,2	4,1
	3600	9,1	64,5	-	9,3	0,765	0,1	69000	34500	34500	3000	3	100	0	0	3,44	18,2	3,8
	3600	9,1	64,5	-	9,3	0,765	0,15	69000	34500	34500	3000	3	100	0	0	3,44	9,9	2,4
**CEM I-0.32-WRA6**	3600	9,1	64,5	-	9,3	0,765	0	56000	24340	24340	1000	3	100	0	0	3,27	81,9	7
	3600	9,1	64,5	-	9,3	0,765	0,1	56000	24340	24340	1000	3	100	0	0	3,27	34,2	5,8
	3600	9,1	64,5	-	9,3	0,765	0,15	56000	24340	24340	1000	3	100	0	0	3,27	24,4	4,8
**CEM I-0.35-WRA6**	3600	9,1	64,5	-	9,3	0,765	0	56000	24340	24340	1000	3	100	0	0	3,27	57,2	4,1
	3600	9,1	64,5	-	9,3	0,765	0,1	56000	24340	24340	1000	3	100	0	0	3,27	17,3	3,8
	3600	9,1	64,5	-	9,3	0,765	0,15	56000	24340	24340	1000	3	100	0	0	3,27	8,2	2,7
**CEM I-0.32-WRA7**	3600	9,1	64,5	-	9,3	0,765	0	57000	27140	27140	3000	3	100	0	0	3,27	81,9	7
	3600	9,1	64,5	-	9,3	0,765	0,1	57000	27140	27140	3000	3	100	0	0	3,27	44,0	6,7
	3600	9,1	64,5	-	9,3	0,765	0,15	57000	27140	27140	3000	3	100	0	0	3,27	18,3	4,4
**CEM I-0.35-WRA7**	3600	9,1	64,5	-	9,3	0,765	0	57000	27140	27140	3000	3	100	0	0	3,27	57,2	4,1
	3600	9,1	64,5	-	9,3	0,765	0,1	57000	27140	27140	3000	3	100	0	0	3,27	16,8	3,9
	3600	9,1	64,5	-	9,3	0,765	0,15	57000	27140	27140	3000	3	100	0	0	3,27	4,3	1,3
**CEM I-0.32-WRA8**	3600	9,1	64,5	-	9,3	0,765	0	58000	26300	26300	2400	3	99	1	0	2,91	81,9	7
	3600	9,1	64,5	-	9,3	0,765	0,1	58000	26300	26300	2400	3	99	1	0	2,91	40,3	5,9
	3600	9,1	64,5	-	9,3	0,765	0,15	58000	26300	26300	2400	3	99	1	0	2,91	14,7	3,3
**CEM I-0.35-WRA8**	3600	9,1	64,5	-	9,3	0,765	0	58000	26300	26300	2400	3	99	1	0	2,91	57,2	4,1
	3600	9,1	64,5	-	9,3	0,765	0,1	58000	26300	26300	2400	3	99	1	0	2,91	15,5	2,9
	3600	9,1	64,5	-	9,3	0,765	0,15	58000	26300	26300	2400	3	99	1	0	2,91	10,5	0,9
**CEM I-0.32-WRA9**	3600	9,1	64,5	-	9,3	0,765	0	65000	27000	27000	2400	3	97	3	0	2,93	81,9	7
	3600	9,1	64,5	-	9,3	0,765	0,1	65000	27000	27000	2400	3	97	3	0	2,93	39,5	6,2

	3600	9,1	64,5	-	9,3	0,765	0,15	65000	27000	27000	2400	3	97	3	0	2,93	15,4	3,5
**CEM I-0.35-WRA9**	3600	9,1	64,5	-	9,3	0,765	0	65000	27000	27000	2400	3	97	3	0	2,93	57,2	4,1
	3600	9,1	64,5	-	9,3	0,765	0,1	65000	27000	27000	2400	3	97	3	0	2,93	15,3	2,9
	3600	9,1	64,5	-	9,3	0,765	0,15	65000	27000	27000	2400	3	97	3	0	2,93	11,3	0,9
**CEM I-0.32-WRA10**	3600	9,1	64,5	-	9,3	0,765	0	61000	26500	26500	2400	3	95	5	0	2,93	81,9	7
	3600	9,1	64,5	-	9,3	0,765	0,1	61000	26500	26500	2400	3	95	5	0	2,93	35,3	5,6
	3600	9,1	64,5	-	9,3	0,765	0,15	61000	26500	26500	2400	3	95	5	0	2,93	12,6	2,9
**CEM I-0.35-WRA10**	3600	9,1	64,5	-	9,3	0,765	0	61000	26500	26500	2400	3	95	5	0	2,93	57,2	4,1
	3600	9,1	64,5	-	9,3	0,765	0,1	61000	26500	26500	2400	3	95	5	0	2,93	11,1	2,8
	3600	9,1	64,5	-	9,3	0,765	0,15	61000	26500	26500	2400	3	95	5	0	2,93	10,5	0,8
**CEM I-0.32-WRA11**	3600	9,1	64,5	-	9,3	0,765	0	56100	26600	26600	2400	3	93	7	0	2,65	81,9	7
	3600	9,1	64,5	-	9,3	0,765	0,1	56100	26600	26600	2400	3	93	7	0	2,65	32,5	5,1
	3600	9,1	64,5	-	9,3	0,765	0,15	56100	26600	26600	2400	3	93	7	0	2,65	12,1	2,9
**CEM I-0.35-WRA11**	3600	9,1	64,5	-	9,3	0,765	0	56100	26600	26600	2400	3	93	7	0	2,65	57,2	4,1
	3600	9,1	64,5	-	9,3	0,765	0,1	56100	26600	26600	2400	3	93	7	0	2,65	15,7	2,7
	3600	9,1	64,5	-	9,3	0,765	0,15	56100	26600	26600	2400	3	93	7	0	2,65	13,5	1,1
**CEM I-0.32-WRA12**	3600	9,1	64,5	-	9,3	0,765	0	53000	23000	23000	2400	3	91	9	0	2,54	81,9	7
	3600	9,1	64,5	-	9,3	0,765	0,1	53000	23000	23000	2400	3	91	9	0	2,54	29,4	4,9
	3600	9,1	64,5	-	9,3	0,765	0,15	53000	23000	23000	2400	3	91	9	0	2,54	20,9	3,1
**CEM I-0.35-WRA12**	3600	9,1	64,5	-	9,3	0,765	0	53000	23000	23000	2400	3	91	9	0	2,54	57,2	4,1
	3600	9,1	64,5	-	9,3	0,765	0,1	53000	23000	23000	2400	3	91	9	0	2,54	11,5	2,5
	3600	9,1	64,5	-	9,3	0,765	0,15	53000	23000	23000	2400	3	91	9	0	2,54	8,9	1,2
**CEM I-0.32-WRA13**	3600	9,1	64,5	-	9,3	0,765	0	52000	22600	22600	2400	3	80	20	0	2,61	81,9	7
	3600	9,1	64,5	-	9,3	0,765	0,1	52000	22600	22600	2400	3	80	20	0	2,61	36,1	5,6
	3600	9,1	64,5	-	9,3	0,765	0,15	52000	22600	22600	2400	3	80	20	0	2,61	25,5	4,2
**CEM I-0.35-WRA13**	3600	9,1	64,5	-	9,3	0,765	0	52000	22600	22600	2400	3	80	20	0	2,61	57,2	4,1
	3600	9,1	64,5	-	9,3	0,765	0,1	52000	22600	22600	2400	3	80	20	0	2,61	15,0	2,7
	3600	9,1	64,5	-	9,3	0,765	0,15	52000	22600	22600	2400	3	80	20	0	2,61	16,0	1,5
**CEM I-0.32-WRA14**	3600	9,1	64,5	-	9,3	0,765	0	62100	29500	29500	2400	3	99	0	1	1,08	81,9	7
	3600	9,1	64,5	-	9,3	0,765	0,1	62100	29500	29500	2400	3	99	0	1	1,08	28,0	5,1
	3600	9,1	64,5	-	9,3	0,765	0,15	62100	29500	29500	2400	3	99	0	1	1,08	13,1	3,2
**CEM I-0.35-WRA14**	3600	9,1	64,5	-	9,3	0,765	0	62100	29500	29500	2400	3	99	0	1	1,08	57,2	4,1
	3600	9,1	64,5	-	9,3	0,765	0,1	62100	29500	29500	2400	3	99	0	1	1,08	11,7	2,4

	3600	9,1	64,5	-	9,3	0,765	0,15	62100	29500	29500	2400	3	99	0	1	1,08	8,8	1,1
**CEM I-0.32-WRA15**	3600	9,1	64,5	-	9,3	0,765	0	63000	28600	28600	2400	3	97	0	3	1,08	81,9	7
	3600	9,1	64,5	-	9,3	0,765	0,1	63000	28600	28600	2400	3	97	0	3	1,08	26,0	5,1
	3600	9,1	64,5	-	9,3	0,765	0,15	63000	28600	28600	2400	3	97	0	3	1,08	13,7	3,2
**CEM I-0.35-WRA15**	3600	9,1	64,5	-	9,3	0,765	0	63000	28600	28600	2400	3	97	0	3	1,08	57,2	4,1
	3600	9,1	64,5	-	9,3	0,765	0,1	63000	28600	28600	2400	3	97	0	3	1,08	9,0	2,5
	3600	9,1	64,5	-	9,3	0,765	0,15	63000	28600	28600	2400	3	97	0	3	1,08	9,1	1,1
**CEM I-0.32-WRA16**	3600	9,1	64,5	-	9,3	0,765	0	58000	29000	29000	2400	3	95	0	5	1,08	81,9	7
	3600	9,1	64,5	-	9,3	0,765	0,1	58000	29000	29000	2400	3	95	0	5	1,08	28,5	5
	3600	9,1	64,5	-	9,3	0,765	0,15	58000	29000	29000	2400	3	95	0	5	1,08	10,8	3
**CEM I-0.35-WRA16**	3600	9,1	64,5	-	9,3	0,765	0	58000	29000	29000	2400	3	95	0	5	1,08	57,2	4,1
	3600	9,1	64,5	-	9,3	0,765	0,1	58000	29000	29000	2400	3	95	0	5	1,08	13,2	3,5
	3600	9,1	64,5	-	9,3	0,765	0,15	58000	29000	29000	2400	3	95	0	5	1,08	9,5	1,2
**CEM I-0.32-WRA17**	3600	9,1	64,5	-	9,3	0,765	0	63000	28600	28600	2400	3	93	0	7	1,08	81,9	7
	3600	9,1	64,5	-	9,3	0,765	0,1	63000	28600	28600	2400	3	93	0	7	1,08	27,5	5,1
	3600	9,1	64,5	-	9,3	0,765	0,15	63000	28600	28600	2400	3	93	0	7	1,08	12,7	2,8
**CEM I-0.35-WRA17**	3600	9,1	64,5	-	9,3	0,765	0	63000	28600	28600	2400	3	93	0	7	1,08	57,2	4,1
	3600	9,1	64,5	-	9,3	0,765	0,1	63000	28600	28600	2400	3	93	0	7	1,08	12,0	2,5
	3600	9,1	64,5	-	9,3	0,765	0,15	63000	28600	28600	2400	3	93	0	7	1,08	9,0	1
**CEM I-0.32-WRA18**	3600	9,1	64,5	-	9,3	0,765	0	60000	25000	25000	2400	3	91	0	9	1,08	81,9	7
	3600	9,1	64,5	-	9,3	0,765	0,1	60000	25000	25000	2400	3	91	0	9	1,08	33,0	5,1
	3600	9,1	64,5	-	9,3	0,765	0,15	60000	25000	25000	2400	3	91	0	9	1,08	15,1	3,3
**CEM I-0.35-WRA18**	3600	9,1	64,5	-	9,3	0,765	0	60000	25000	25000	2400	3	91	0	9	1,08	57,2	4,1
	3600	9,1	64,5	-	9,3	0,765	0,1	60000	25000	25000	2400	3	91	0	9	1,08	13,4	2
	3600	9,1	64,5	-	9,3	0,765	0,15	60000	25000	25000	2400	3	91	0	9	1,08	11,5	0,9
**CEM I-0.32-WRA19**	3600	9,1	64,5	-	9,3	0,765	0	61000	29000	29000	2400	3	80	0	20	1,08	81,9	7
	3600	9,1	64,5	-	9,3	0,765	0,1	61000	29000	29000	2400	3	80	0	20	1,08	35,5	5,5
	3600	9,1	64,5	-	9,3	0,765	0,15	61000	29000	29000	2400	3	80	0	20	1,08	23,9	4,2
**CEM I-0.35-WRA19**	3600	9,1	64,5	-	9,3	0,765	0	61000	29000	29000	2400	3	80	0	20	1,08	57,2	4,1
	3600	9,1	64,5	-	9,3	0,765	0,1	61000	29000	29000	2400	3	80	0	20	1,08	16,7	3,2
	3600	9,1	64,5	-	9,3	0,765	0,15	61000	29000	29000	2400	3	80	0	20	1,08	13,2	1,2
**CEM I-0.32-WRA20**	3600	9,1	64,5	-	9,3	0,765	0	63000	30000	30000	2400	3	100	0	0	3,54	81,9	7
	3600	9,1	64,5	-	9,3	0,765	0,1	63000	30000	30000	2400	3	100	0	0	3,54	36,3	5,9

	3600	9,1	64,5	-	9,3	0,765	0,15	63000	30000	30000	2400	3	100	0	0	3,54	46,1	3,8
**CEM I-0.35-WRA20**	3600	9,1	64,5	-	9,3	0,765	0	63000	30000	30000	2400	3	100	0	0	3,54	57,2	4,1
	3600	9,1	64,5	-	9,3	0,765	0,1	63000	30000	30000	2400	3	100	0	0	3,54	13,4	2,9
	3600	9,1	64,5	-	9,3	0,765	0,15	63000	30000	30000	2400	3	100	0	0	3,54	8,8	1,3
**CEM I-0.32-WRA21**	3600	9,1	64,5	-	9,3	0,765	0	56000	26500	26500	2400	3	100	0	0	2,94	81,9	7
	3600	9,1	64,5	-	9,3	0,765	0,1	56000	26500	26500	2400	3	100	0	0	2,94	21,2	4,8
	3600	9,1	64,5	-	9,3	0,765	0,15	56000	26500	26500	2400	3	100	0	0	2,94	13,8	3,5
**CEM I-0.35-WRA21**	3600	9,1	64,5	-	9,3	0,765	0	56000	26500	26500	2400	3	100	0	0	2,94	57,2	4,1
	3600	9,1	64,5	-	9,3	0,765	0,1	56000	26500	26500	2400	3	100	0	0	2,94	11,6	2,6
	3600	9,1	64,5	-	9,3	0,765	0,15	56000	26500	26500	2400	3	100	0	0	2,94	3,1	1,1
**CEM I-0.32-WRA22**	3600	9,1	64,5	-	9,3	0,765	0	60000	26000	26000	2400	3	91	9	0	2,97	81,9	7
	3600	9,1	64,5	-	9,3	0,765	0,1	60000	26000	26000	2400	3	91	9	0	2,97	27,1	7,2
	3600	9,1	64,5	-	9,3	0,765	0,15	60000	26000	26000	2400	3	91	9	0	2,97	38,1	5,3
**CEM I-0.35-WRA22**	3600	9,1	64,5	-	9,3	0,765	0	60000	26000	26000	2400	3	91	9	0	2,97	57,2	4,1
	3600	9,1	64,5	-	9,3	0,765	0,1	60000	26000	26000	2400	3	91	9	0	2,97	14,6	3,4
	3600	9,1	64,5	-	9,3	0,765	0,15	60000	26000	26000	2400	3	91	9	0	2,97	8,9	1,7
**CEM I-0.32-WRA23**	3600	9,1	64,5	-	9,3	0,765	0	54000	22500	22500	2400	3	91	9	0	3,21	81,9	7
	3600	9,1	64,5	-	9,3	0,765	0,1	54000	22500	22500	2400	3	91	9	0	3,21	40,6	6,5
	3600	9,1	64,5	-	9,3	0,765	0,15	54000	22500	22500	2400	3	91	9	0	3,21	20,7	4
**CEM I-0.35-WRA23**	3600	9,1	64,5	-	9,3	0,765	0	54000	22500	22500	2400	3	91	9	0	3,21	57,2	4,1
	3600	9,1	64,5	-	9,3	0,765	0,1	54000	22500	22500	2400	3	91	9	0	3,21	16,2	3
	3600	9,1	64,5	-	9,3	0,765	0,15	54000	22500	22500	2400	3	91	9	0	3,21	9,5	1,8
**CEM I-0.32-WRA24**	3600	9,1	64,5	-	9,3	0,765	0	62000	29000	29000	2400	3	91	0	9	3,24	81,9	7
	3600	9,1	64,5	-	9,3	0,765	0,1	62000	29000	29000	2400	3	91	0	9	3,24	34,9	5,6
	3600	9,1	64,5	-	9,3	0,765	0,15	62000	29000	29000	2400	3	91	0	9	3,24	27,9	4,5
**CEM I-0.35-WRA24**	3600	9,1	64,5	-	9,3	0,765	0	62000	29000	29000	2400	3	91	0	9	3,24	57,2	4,1
	3600	9,1	64,5	-	9,3	0,765	0,1	62000	29000	29000	2400	3	91	0	9	3,24	13,5	3,3
	3600	9,1	64,5	-	9,3	0,765	0,15	62000	29000	29000	2400	3	91	0	9	3,24	13,9	1,5
**CEM I-0.32-WRA25**	3600	9,1	64,5	-	9,3	0,765	0	56000	28000	28000	2400	3	91	0	9	3,58	81,9	7
	3600	9,1	64,5	-	9,3	0,765	0,1	56000	28000	28000	2400	3	91	0	9	3,58	26,4	5,2
	3600	9,1	64,5	-	9,3	0,765	0,15	56000	28000	28000	2400	3	91	0	9	3,58	13,1	3,2
**CEM I-0.35-WRA25**	3600	9,1	64,5	-	9,3	0,765	0	56000	28000	28000	2400	3	91	0	9	3,58	57,2	4,1
	3600	9,1	64,5	-	9,3	0,765	0,1	56000	28000	28000	2400	3	91	0	9	3,58	11,9	3,2

	3600	9,1	64,5	-	9,3	0,765	0,15	56000	28000	28000	2400	3	91	0	9	3,58	3,0	1
